# Stereoselective photoredox ring-opening polymerization of *O*-carboxyanhydrides

**DOI:** 10.1038/s41467-018-03879-5

**Published:** 2018-04-19

**Authors:** Quanyou Feng, Lei Yang, Yongliang Zhong, Dong Guo, Guoliang Liu, Linghai Xie, Wei Huang, Rong Tong

**Affiliations:** 1 Institute of Optoelectronic Materials, Key Laboratory for Organic Electronics and Information Displays, Jiangsu Key Laboratory for Biosensors, Institute of Advanced Materials, Jiangsu National Synergetic Innovation Center for Advanced Materials, Nanjing University of Posts and Telecommunications, 9 Wenyuan Road, Nanjing, 210023 China; 2 Department of Chemical Engineering, Virginia Polytechnic Institute and State University, 635 Prices Fork Road, Blacksburg, Virginia 24061 United States; 3 Department of Chemistry, Virginia Polytechnic Institute and State University, 800 West Campus Drive, Blacksburg, Virginia 24061 United States

## Abstract

Biodegradable polyesters with various tacticities have been synthesized by means of stereoselective ring-opening polymerization of racemic lactide and β-lactones but with limited side-chain groups. However, stereoselective synthesis of functional polyesters remains challenging from *O*-carboxyanhydrides that have abundant pendant side-chain functional groups. Herein we report a powerful strategy to synthesize stereoblock polyesters by stereoselective ring-opening polymerization of racemic *O*-carboxyanhydrides with the use of photoredox Ni/Ir catalysts and a selected Zn complex with an achiral ligand. The obtained stereoblock copolymers are highly isotactic with high molecular weights ( > 70 kDa) and narrow molecular weight distributions (*M*_w_/*M*_n_ < 1.1), and they display distinct melting temperatures that are similar to their stereocomplex counterparts. Furthermore, in one-pot photoredox copolymerization of two different *O*-carboxyanhydrides, the use of such Zn complex mediates kinetic resolution of the comonomers during enchainment and shows a chirality preference that allows for the synthesis of gradient copolymers.

## Abstract (web summary)

The stereoselective synthesis of polyesters has been achieved via various methods; however, all routes show limited side-chain group functionality. Here the authors synthesize stereoblock polyesters by photoredox ring-opening polymerization of racemic *O*-carboxyanhydrides that have abundant side-chain functionality.

## Introduction

**Fig. 1:**
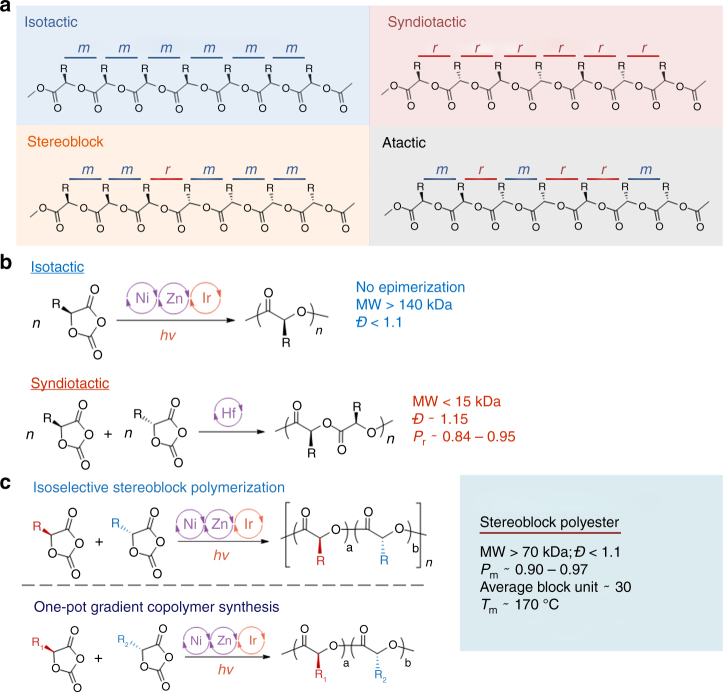
Stereoselective ring-opening polymerization of *O*-carboxyanhydrides (OCAs). **a** Common tacticities of poly(α-hydroxy acid)s. **b** Previously reported strategies for controlling the stereochemistry of OCA polymerization. **c** Stereoselective photoredox ring-opening copolymerization of OCAs reported in this paper

One of the most critical determinants of the physical and mechanical properties of a polymer is its tacticity^1–3^, that is, the regularity of the configurations of adjacent stereocenters along the polymer chain^4^. Two basic ordered structures can occur: isotactic structures, in which the configurations of neighboring stereocenters are the same, and syndiotactic structures, in which neighboring stereocenters have alternating configurations (Fig. 1a). Polymers with isotactic or syndiotactic structures, referred to as stereoregular polymers, usually are crystalline and have improved thermal and mechanical properties relative to those of atactic polymers. For example, isotactic polypropylene is a semicrystalline material with a melting temperature (*T*_m_) of 130–175 °C, whereas atactic (amorphous) polypropylene is viscous and does not have a specific *T*_m_^5^. Stereoregular polymers can be synthesized by the use of homogeneous single-site catalysts to control the chain-end reactivity and stereoselectivity^5–12^.

Polyesters, including polylactide, are biocompatible, biodegradable materials with a wide variety of applications ranging from clothing and packaging to agricultural and biomedical uses^13–16^. Polyesters with various microstructures have been synthesized by means of stereoselective ring-opening polymerization (ROP) of racemic lactide and β-lactones^17–33^, including the use of newly developed switchable catalysts^34–36^. However, stereoselective synthesis of polyesters with pendant side-chain functional groups remains challenging, owing in part to the lack of side-chain functionality in the lactone monomers^37, 38^. In 2006, *O*-carboxyanhydrides (OCAs) emerged as alternatives to lactones;^39^ OCAs can be prepared with a rich variety of side-chain functionalities and are highly active monomers for ROP^38–40^. We recently reported a protocol for controlled photoredox ROP of enantiomerically pure OCAs that affords isotactic polyesters with expected molecular weights (MWs, > 140 kDa) and narrow MW distributions (*Đ* < 1.1, Fig. 1b)^41^. Nevertheless, only low-MW ( < 20 kDa) syndiotactic polyesters synthesized from racemic OCAs has been achieved very recently, presumably via the chain-end-control mechanism (Fig. 1b)^42^.

In this study, we extend our previously reported photoredox ROP protocol to accomplish stereoselective ROP of OCAs, and we present a new approach to the synthesis of stereoblock polyesters, as well as the formation of gradient copolymers (Fig. 1c).

## Results

### Identification of Zn complexes with tridentate ligands for photoredox ROP

**Fig. 2:**
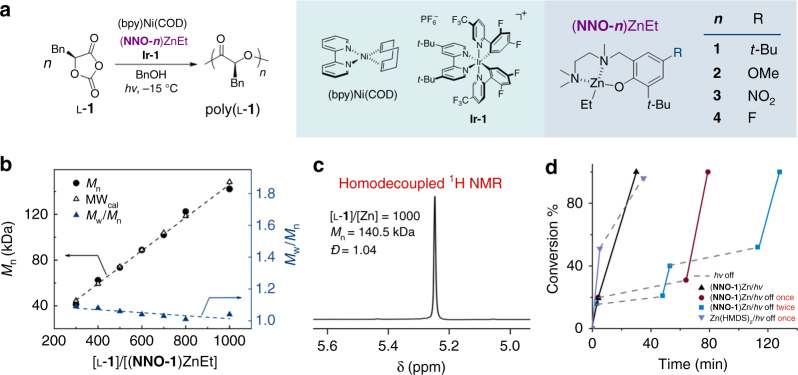
Controlled photoredox ring-opening polymerizations (ROPs) mediated by (**NNO-1**)ZnEt. **a** Photoredox ROP of l-**1** mediated by various tridentate (**NNO-*n***)ZnEt complexes. **b** Plots of *M*_n_ and molecular weight distribution (*M*_w_/*M*_n_) of poly(l-**1**) versus [l-**1**]/[(**NNO-1**)ZnEt] ratio ([(bpy)Ni(COD)]/[(**NNO-1**)ZnEt]/[BnOH]/[**Ir-1**] = 1/1/1/0.1). **c** Homodecoupled ^1^H NMR spectrum of the α-methine region of poly(l-**1**) with an [l-**1**]/[Zn] ratio of 1000. **d** Dependence of the rate of l-**1** conversion on the presence or absence of light. All reactions were performed at a [l-**1**]/[Ni]/[Zn]/[BnOH]/[**Ir-1**] ratio of 600/1/1/1/0.1 at −15 °C. The dashed lines indicate periods during which the reaction mixture was held in the dark at −30 °C, and solid lines indicate period during which the reaction mixture was irradiated at −15 °C

The sterically bulky β-diiminate Zn complexes do not initiate controlled polymerization of l-**1** at a [l-**1**]/[Zn] ratio of 300, whereas a less bulky tridentate Schiff base ligand (**NNO-1**) does (Fig. 2a)^41^. We then examined various NNO ligand substituents, expecting that the electronic nature of the ligand would influence the Lewis acidity of the Zn atom and thereby affect the polymerization. We synthesized three additional (**NNO-*n***)ZnEt complexes, one with an electron-donating substituent (OMe) and two with an electron-withdrawing substituent (F or NO_2_) at the 5-position of the salicylidene moiety (Fig. 2a). All of the complexes were then tested in the photoredox ROP of l-**1** at −15 °C at a [l-**1**]/[Zn] ratio of 500 under irradiation with blue LEDs (400–500 nm) in the presence of a combination of (bpy)Ni(COD) and Ir[dF(CF_3_)ppy]_2_(dtbbpy)PF_6_ (**Ir-1**) with benzyl alcohol (BnOH) as the initiator ([l-**1**]/[Ni]/[Zn]/[BnOH]/[**Ir-1**] = 500/1/1/1/0.1, Fig. 2a), as described previously^41^. Reactions carried out with the complexes bearing electron-donating ligands, **NNO-1** and **NNO-2**, afforded polymers with number-average MW (*M*_n_) values close to the expected value (74.1 kDa) and with narrow *Đ* values (*Đ* *=* *M*_w_/*M*_n_) of approximately 1.05, and monomer conversion was quantitative (Table 1, entries 1 and 2). Uncontrolled polymerization occurred when using Zn complexes bearing electron-withdrawing groups, **NNO-3** and **NNO-4** (entries 3 and 4). When (**NNO-1**)ZnEt was used for photoredox polymerization, *M*_n_ of the poly(l-**1**) product increased linearly with initial [l-**1**]/[Ni]/[Zn]/[**Ir-1**] ratio up to 1000/1/1/0.1, and the *Ð* values of all of the obtained polymers were < 1.1 (Fig. 2b; Supplementary Fig. 1), which suggest that chain-breaking reactions did not occur during the ROP. Notably, no epimerization of the α-methine hydrogen was observed in the homodecoupled ^1^H NMR spectra, even for high-MW poly(l-**1**) (Table 1, entry 5; *M*_n_ = 140.5 kDa; Fig. 2c), indicating that the Zn complexes did not affect the chirality of l-**1** during the ROP. We also used the (**NNO-1**)ZnEt complex for photoredox ROP reactions of other OCA monomers (l-**2**, l-**3**, and **l-4**; entries 6–8). In all cases, polymerization control was excellent, similar to that for the formation of poly(l-**1**); the *M*_n_ values of all the obtained polymers were close to the calculated MWs, all the *Ð* values were < 1.1, and the α-methine hydrogen did not epimerize (Supplementary Figs. 2–5).

**Table 1:** Screening of Zn complex ligands for photoredox ring-opening polymerization of l-**1**


Entry	Zn complex	Monomer	FR^a^	Conv. (%)^b^	*M*_n_ (kDa)^c^	MW_cal_ (kDa)	*Ð* ^c^
1	(**NNO-1**)ZnEt	L-**1**	500	100	73.5	74.1	1.06
2	(**NNO-2**)ZnEt	L-**1**	500	100	70.4	74.1	1.05
3	(**NNO-3**)ZnEt	L-**1**	500	100	48.1	74.1	1.03
4	(**NNO-4**)ZnEt	L-**1**	500	100	56.1	74.1	1.04
5	(**NNO-1**)ZnEt	L-**1**	1000	100	140.5	148.1	1.04
6	(**NNO-1**)ZnEt	L-**2**	200	100	27.4	35.7	1.01
7	(**NNO-1**)ZnEt	L-**3**	200	100	32.8	44.1	1.03
8	(**NNO-1**)ZnEt	L-**4**	200	100	12.0	14.5	1.05

FR, the feeding ratio of [OCA]/[Zn]; Conv., monomer conversion; Temp., temperature; *M*_n_, number-average molecular weight; MW_cal_, molecular weight calculated from the ratio of the amount of monomer to the amount of catalyst; *Ð*, molecular weight distribution

^*a*^ FR is the ratio of the amount of monomer to the amount of Zn catalyst

^*b*^ Determined from the intensity of the Fourier transform infrared peak at 1805 cm^−1^, which corresponds to the anhydride group of the OCA

^*c*^ Determined by gel-permeation chromatography

Notably, during our polymerization studies, we found that the presence of **NNO-1** ligand in the Zn complex allowed us to modify the polymerization kinetics by turning the light on or off. Specifically, in the presence of (**NNO-1**)ZnEt at a [l-**1**]/[Zn] ratio of 600, irradiation of the reaction solution for 4 min resulted in 22% conversion of l-**1**. If the reaction mixture was then placed in the dark at −30 °C for 60 min, conversion of l-**1** increased only slightly, to 31%; but once irradiation was resumed, l-**1** was rapidly consumed, within 15 min (Fig. 2d, red line). In contrast, in a similar reaction mediated by zinc *bis*[*bis*(trimethylsilyl)amide] (Zn(HMDS)_2_), consumption of l-**1** continued even after the light was turned off at −30 °C (Fig. 2d, green line). In the presence of (**NNO-1**)ZnEt, the photoredox ROP could be slowed down and speeded up multiple times: each time the light was turned off, the ROP almost stopped, and it again proceeded rapidly when irradiation was resumed (Fig. 2d, blue line). In theory, this phenomenon would allow us to monitor the reaction kinetics and analyze intermediates using NMR by temporarily quenching polymerization in the dark at low temperature.

### Stereocontrolled photoredox polymerization of *rac*-OCAs

**Fig. 3:**
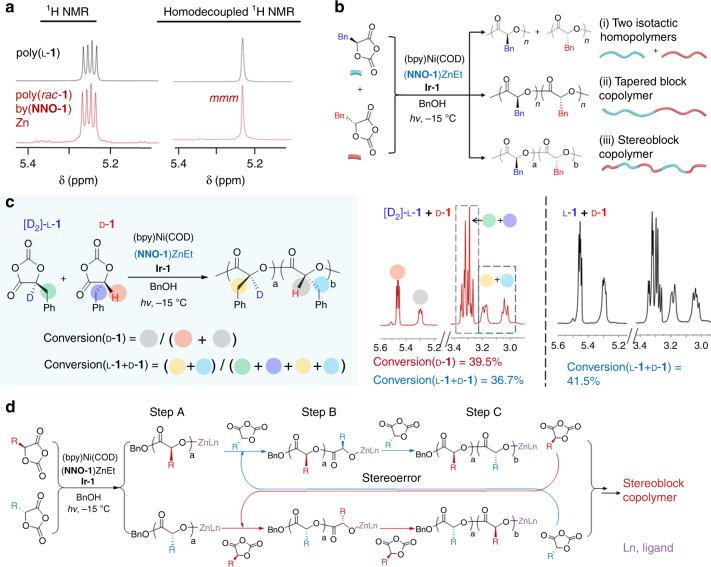
Synthesis and microstructure analysis of the stereoblock copolymer synthesized from *rac*-**1**. **a** Comparison of ^1^H and homodecoupled ^1^H NMR spectra of highly isotactic poly(*rac*-**1**) and poly(l-**1**). **b** Possible microstructures of highly isotactic poly(*rac*-**1**). **c** Use of [D_2_]-l-**1** to study the kinetics of *rac*-**1** polymerization. The conversions of d-**1** and *rac*-**1** could be determined via analysis of the α-methine region (~5.4 ppm) and the methyl region (~3.2 ppm), respectively, of the ^1^H NMR spectra. Both spectra were recorded at 18 min under the same reaction conditions: [l-**1**]/[d-**1**]/[Ni]/[Zn]/[BnOH]/[**Ir-1**] = 150/150/1/1/1/0.1, −20 °C, [d-**1**] = 76.98 mM. **d** Proposed mechanism of the formation of a stereoblock copolymer by means of photoredox ring-opening polymerization of racemic *O*-carboxyanhydrides

We investigated Zn complexes with the **NNO-1** and **NNO-2** ligands in the photoredox polymerization of *rac*-**1** by mixing l-**1** and d-**1** at a 1/1 ratio. We discovered that within 4 h of irradiation at –15 °C, photoredox ROP mediated by (**NNO-1**)ZnEt ([l-**1**]/[d-**1**]/[Zn]/[Ni]/[BnOH]/[**Ir-1**] = 150/150/1/1/1/0.1) resulted in a product with a *M*_n_ of 45.7 kDa, which was close to the expected MW (44.5 kDa), and a narrow *Đ* (1.06) (Table 2, entry 1). In contrast, a similar reaction mediated by (**NNO-2**)ZnEt showed less well controlled polymerization (compare entries 1 and 2). Homonuclear decoupled ^1^H NMR analysis of the microstructure of the polymer formed in the (**NNO-1**)ZnEt-mediated reaction revealed a high degree of isotacticity, as evidenced by the large *mmm* tetrad peak (*m*-dyad represents two identically oriented units [*meso*]) in the methine region (δ ≈ 5.2 ppm; Fig. 3a and Supplementary Fig. 6b). The highest probability of *meso* dyad formation (*P*_m_, i.e., isotactic enchainment)^43, 44^ was 0.97, as calculated by analysis of the methine region in the homonuclear decoupled ^1^H NMR spectrum (Fig. 3a and Supplementary Fig. 6). This distinct preference for isotactic enchainment was confirmed by analysis of the ^13^C NMR spectrum of the methine region (δ ≈ 73.4 ppm; Supplementary Fig. 6c).

**Table 2:** Stereoselective controlled photoredox ring-opening polymerization of racemic *O*-carboxyanhydrides

Entry	Zn complex	Monomer	FR^a^	Time	Conv. (%)^b^	*M*_n_ (kDa)^c^	MW_cal_ (kDa)	*Ð* ^c^	*P*_m_^d^
1	(**NNO-1**)ZnEt	l-**1** / d-**1**	150/150	4	100	45.7	44.5	1.06	0.97
2	(**NNO-2**)ZnEt	l-**1** / d-**1**	150/150	4	100	35.5	44.5	1.02	0.93
3	(**NNO-1**)ZnEt	l-**2** / d-**2**	100/100	7	100	29.1	35.7	1.05	0.88
4	(**NNO-1**)ZnEt	l-**3** / d-**3**	100/100	6	100	32.6	44.2	1.01	0.94
5	(**NNO-1**)ZnEt	l-**4** / d-**4**	100/100	4	100	12.4	14.5	1.04	0.92
6	Zn(HMDS)_2_	l-**1** / d-**1**	150/150	4	100	78.2	44.5	1.16	0.66
7	(**NNO-1**)ZnEt	l-**1** / d-**1**	200/100	4	100	40.6	44.5	1.02	0.93
8 ^e^	(**NNO-1**)ZnEt	l-**1** / d-**1**	300/300	7	100	78.0	88.9	1.04	0.93

FR, the feeding ratio of [OCA]/[Zn]; Conv., monomer conversion; Temp., temperature; *M*_n_, number-average molecular weight; MW_cal_, molecular weight calculated from the ratio of the amount of monomer to the amount of catalyst; *Ð*, molecular weight distribution; *P*_m_, maximum probability of meso dyads

^a^ FR is the ratio of the amount of monomer to the amount of catalyst

^b^ Determined from the intensity of the Fourier transform infrared peak at 1805 cm^−1^, which corresponds to the anhydride group of the OCA

^c^ Determined by gel-permeation chromatography

^d^ Determined by homodecoupled ^1^H NMR and ^13^C NMR spectroscopy. See Supplementary Figs. 6–9 and Supplementary Table 1

^e^ Two equivalents of (bpy)Ni(COD) was used ([l-**1**]/[d-**1**]/[(bpy)Ni(COD)]/[(**NNO-1**)ZnEt]/[BnOH]/[**Ir-1**] = 300/300/2/1/1/0.1)

To evaluate the utility of (**NNO-1**)ZnEt for stereoselective ROP, we carried out photoredox ROP reactions of three other racemic OCA monomers (**2–4**; Table 2, entries 3–5). In all cases, polymerization control was excellent, similar to that observed for the formation of poly(*rac*-**1**); the *M*_n_ values of all the polymers were close to the expected MWs, and the *Ð* values were < 1.1. Microstructural analysis showed isotactic enchainment control with *P*_m_ values ranging from 0.89 to 0.97 (Supplementary Figs. 7–9; Supplementary Table 1). In contrast, Zn(HMDS)_2_-mediated photoredox polymerization of *rac*-**1** afforded an atactic polymer (Supplementary Fig. 10; Table 2, entry 6), confirming that the **NNO-1** ligand influenced the stereoselectivity of OCA polymerization. Because the (**NNO-1**)ZnEt catalyst does not have isomer^45^ and polymerization rate constants were similar for both enantiomers (Supplementary Fig. 11a–c), our results suggest a chain-end-control mechanism in which the chirality of the propagating chain end bound to the catalyst determines the chirality of the next monomer to be enchained^18, 33, 46, 47^.

### Microstructure and kinetics analysis

Three different microstructures may have contributed to the high isotacticity of poly(*rac*-**1**) obtained from the reaction mediated by (**NNO-1**)ZnEt: two isotactic polymers (poly(l-**1**) and poly(d-**1**)), a tapered block copolymer, and a stereoblock copolymer with alternating blocks of l-**1** and d-**1** (Fig. 3b). To gain insight into the microstructure of the obtained poly(*rac*-**1**), we first examined the kinetics of the photoredox ROPs of l-**1** and d-**1** separately by varying the concentration of (**NNO-1**)ZnEt and monitoring monomer conversion by Fourier transform infrared spectroscopy^7, 18^ .First-order reaction kinetics were observed for both monomers, and the polymerization rate constants were similar (at [**1**]/[Zn] = 500, the constants were 0.128 min^−1^ for l-**1** and 0.117 min^−1^ for d-**1**; Supplementary Fig. 11a–c). Notably, the reaction order with respect to (**NNO-1**)ZnEt was 1.13 ± 0.03 (Supplementary Fig. 11d-e), suggesting that (**NNO-1**)Zn resides in a monomeric form during photoredox ROP, different from that of Zn(HMDS)_2_ reported in our previous study^41^. Similarly, the photoredox polymerization of *rac*-**1** ([l-**1**]/[d-**1**] = 1/1) exhibits first-order reaction kinetics (Supplementary Fig. 11f), whereas the reaction order with respect to (**NNO-1**)ZnEt was 1.04 ± 0.08, indicating a monomeric form (Supplementary Fig. 11g-h).

Considering the difficulty of distinguishing l-**1** from d-**1** by either Fourier transform infrared spectroscopy or NMR spectroscopy, we prepared deuterated [D_2_]-l-**1** from [D_2_]-l-phenylalanine because the methine deuterium in [D_2_]-l-**1** does not show up in the ^1^H NMR spectrum (Supplementary Fig. 12). We then studied the kinetics of the photoredox ROP of poly(*rac*-**1**) by mixing [D_2_]-l-**1** and d-**1** at a 1/1 molar ratio ([[D_2_]-l-**1**]/[d-**1**]/[Ni]/[Zn]/[BnOH]/[**Ir-1**] = 150/150/1/1/1/0.1, [d-**1**] = 76.98 mM) at −20 °C. After reaction for 18 min, 39.5% of the d-**1** had been converted, as determined by integration of the methine region (~5.2 ppm) in the ^1^H NMR spectrum; whereas total monomers’ conversion ([D_2_]-l-**1** and d-**1**) was 36.7%, as determined by integration of the methylene region (~3.2 ppm) in the ^1^H NMR spectrum (Fig. 3c). In comparison, at the same time point, 41.5% of both enantiomers had been converted (as indicated by ^1^H NMR spectroscopy) in a reaction in which [D_2_]-l-**1** was replaced with l-**1** (Fig. 3c). These results suggest that the polymer chain end did not have a kinetic preference for a specific enantiomer in the (**NNO-1**)ZnEt-mediated photoredox copolymerization of *rac*-**1** and, therefore, that the possibility of a tapered block copolymer microstructure can be excluded (Fig. 3b(ii)).

In addition, because the copolymerization of *rac*-**1** was much slower than the copolymerization of either enantiomer separately at the same [**1**]/[Zn] ratio under the same reaction conditions (at a [**1**]/[Zn] ratio of 300, the polymerization rate constant of l-**1** was 54.9-times higher than that of *rac*-**1**; Supplementary Fig. 11i), it is unlikely that two isotactic polymers, poly(l-**1**) and poly(d-**1**), formed separately (Fig. 3b(i)), which also excludes the possibility of enantiomorphic-site-control mechanism for this isospecific polymerization^19, 46, 48, 49^. Note that changing the [l-**1**]/[d-**1**] ratio from 1/1 to 2/1 did not markedly affect the MW, *Ð*, or *P*_m_ (Table 2, entry 7), a result that suggests that the two enantiomers were copolymerized into one polymer. Taken together, the results of this series of experiments are completely consistent with the formation of a stereoblock copolymer (Fig. 3b(iii)). This stereoblock copolymer should have -*RRRRRRSSSSSS*- sequences in the main chain; in the Bovey designation this type of sequence is designated as -*mmmmrmmmm*-, with *mmr*, *mrm*, and *rmm* tetrads in a predicted ratio of 1/1/1 (Supplementary Fig. 6; Supplementary Table 1). Randall’s equation for calculating the average sequence length (*n*)^50, 51^,

*n* = (*I*_*rrr*_ + 1/2*I*_*rrm*_ + 2*I*_*rmr*_ + 3/2*I*_*mmr*_ + *I*_*mmm*_)/(*I*_*rrr*_ + 1/2*I*_*rrm*_ + *I*_*rmr*_ + 1/2*I*_*mmr*_)

where *I*_*mmr*_, *I*_*mmm*_, and so on are the respective NMR areas, and indicate that the stereoblock copolymer contained an average of 30 units (*n*) of enantiomerically pure **1**.

### Melting temperatures of stereoblock copolymers

Stereochemistry can affect the physicochemical properties of polymers. Interestingly, poly(*sb*-**1**) (Supplementary Table 2, entry 1; *sb*, stereoblock) had a melting temperature (*T*_m_) of 172 °C and a glass transition temperature (*T*_g_) of 50 °C; whereas both isotactic poly(l-**1**) and poly(*rac*-**1**) only had *T*_g_s around 50 °C and no observable *T*_m_s (entries 2–3), which was also the case for the block copolymer poly(l-**1-***b***-d-1**) (entry 4; Supplementary Fig. 13). Note that a 1/1 stereocomplex of poly(l-**1**) and poly(d-**1**) exhibited a *T*_m_ of 178 °C as a result of co-crystallinity of the polymers (entry 5; Supplementary Fig. 13). The fact that poly(*sb*-**1**) showed a *T*_m_ was likely due to its increased crystallinity compared to poly(l-**1**), as has been shown to be the case for stereocomplexed polylactides^52^. Similarly, poly(*sb*-**4**) (entry 6) had a *T*_m_ of 162 °C and a *T*_g_ of 52 °C, and poly(l-**4**) had a *T*_m_ of 169 °C without observable *T*_g_ (entry 7; Supplementary Fig. 13); these values are consistent with the values reported in the literature^19, 52, 53^.

### Enhancement of the kinetics of photoredox ROP of *rac*-OCAs by Ni complexes

As Zn alkoxide mediates chain propagation during stereoselective polymerization, we investigated how Ni complexes affected photoredox ROP of *rac*-**1**. We discovered that the use of Ni(0) complexes with bulky ligands such as tricyclohexylphosphine did not result in controlled photoredox ROP of *rac*-**1**, unlike the use of bpy complexes (Supplementary Table 3). We also found that the presence of a Ni complex and the use of photoredox conditions could markedly improve the reaction kinetics. ROP of *rac*-**1** at low [**1**]/[Zn] ratios in the dark with only (**NNO-1**)ZnEt and BnOH ([l-**1**]/[d-**1**]/[Zn]/[BnOH] = 50/50/1/1) showed incomplete monomer conversion (32.4% at 160 min; compare Supplementary Figs. 14(a) and 11). By replacing l-**1** with [D_2_]-l-**1** in *rac*-**1**, we determined conversion of d-**1** to be 34.2% at 160 min (Supplementary Fig. 14(b)). This result suggests that there was no kinetic preference of a specific enantiomer in this reaction, a result that is similar to the result observed for the polymerization in the presence of Ni under photoredox conditions (Fig. 3c). Notably, the polymer obtained in the absence of Ni displayed a reasonable degree of isotacticity, with a *P*_m_ of 0.85, as determined from the homonuclear decoupled ^1^H NMR spectrum (Supplementary Fig. 14(d)). The improvement in the kinetics resulting from the use of the Ni complex and photoredox conditions likely contributed to the increased isotacticity (*P*_m_ of 0.97 in Table 2, entry 1).

Additionally, increasing the amount of (bpy)Ni from 1 to 2 equivalent markedly enhanced the kinetics of the photoredox ROP of *rac*-**1** at high [**1**]/[Zn] ratios ([l-**1**]/[d-**1**]/[Ni]/[Zn]/[BnOH]/[**Ir-1**] = 300/300/2/1/1/0.1), without having any obvious effect on MW or *P*_m_ (Table 2, entry 8; Supplementary Fig. 15). Increasing the Ni ratio thus allowed for the preparation of high-MW stereoblock copolymers: *M*_n_ increased linearly with initial [l-**1**]/[d-**1**]/[(**NNO-1**)Zn] ratio up to 300/300/1, and all the *Ð* values were < 1.1 (Supplementary Table 4, Supplementary Fig. 16). These results suggest that the use of Ni under photoredox conditions accelerated the polymerization rates but had no effect on the chain-end stereochemistry preference.

### Mechanism of stereoblock copolymer formation

**Fig. 4:**
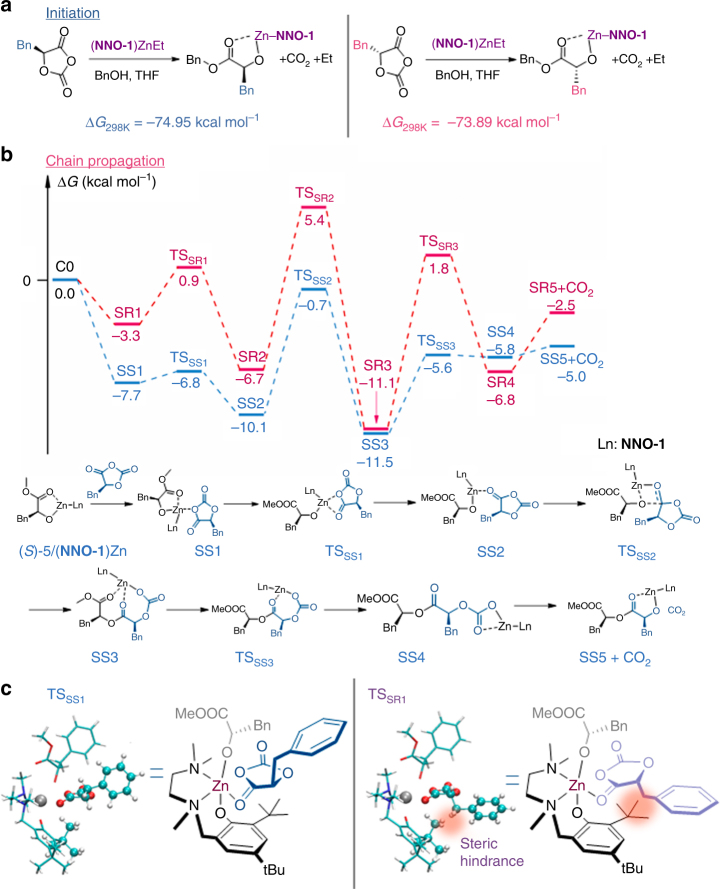
Quantum chemical simulations of isoselective stereoblock copolymerization of *rac*-**1**. **a** Computation of the free energies of ring-opening reaction of **1** mediated by (**NNO-1**)ZnEt/BnOH, which mimics the initiation of the polymerization. **b** Energy profiles of the proposed ring-opening reaction mechanism of *rac*-**1** mediated by (**NNO-1**)Zn/(*S*)-**5**, which mimics the isoselective chain-propagation step in the stereoselective ROP. **c** Stereochemical model for transition states TS_SS1_ and TS_SR1_ for ring-opening reactions in **b**. The steric interaction between the *t*-Bu group in **NNO-1** and (*R*)-**1** (both presented in ball-stick model) that leads to the higher energy, unfavorable TS_SR1_ state is highlighted in orange

To better understand the mechanism of (**NNO-1**)Zn-mediated stereoselective polymerization of *rac*-**1**, we performed density function theory simulations to determine structures, reaction paths and transition states for the initiation and chain-growth steps. Free energies for all reported structures were obtained using the B3LYP hybrid density functional (see Computational Methods in Supplementary Information)^54, 55^. Note that (bpy)Ni/**Ir-1** were not included in simulations because the use of (bpy)Ni/**Ir-1** improved the polymerization kinetics but not affected the stereoselectivity (Supplementary Fig. 14). These atomistic simulation results provide a structural explanation for the observed stereoselectivity of catalysis (Fig. 4). To mimic the initiation step in ROP mediated by (**NNO-1**)ZnEt, we computed (**NNO-1**)ZnEt/BnOH–mediated ring-opening reaction of (*R*)-**1** or (*S*)-**1** ((*S*)-**1** **=** l-**1**; Fig. 4a). The free energy of the (**NNO-1**)ZnEt-mediated ring-opening reaction of (*S*)-**1** was 1.06 kcal mol^−1^ lower than that of (*R*)-**1** (Fig. 4a). That insignificant free energy difference indicates that there is no stereoselectivity in the initiation step (for comparison, the rotational energy barrier in ethane is around 2.8 kcal mol^−1^)^56–58^, which agrees with the kinetic studies of the photoredox ROPs of the two enantiomer (Supplementary Fig. 11c). We then computed the (**NNO-1**)Zn/methyl (*S*)-phenyllactate ((*S*)-**5**)–mediated ring-opening reaction of (*R*)-**1** or (*S*)-**1** that mimics the propagation step in ROP mediated by (**NNO-1**)ZnEt (Fig. 4b; XYZ coordinates and Gibbs free energy of all structures in Supplementary Data 1). Interestingly, (*R*)-**1** and (*S*)-**1** take different orientations for their benzyl groups in the energy transition states TS_SS1_ and TS_SR1_ (Fig. 4c). The benzyl group of (*R*)-**1** sterically clashes with the proximate *tert*-butyl group on **NNO-1** ligand, which causes a shift to a higher energy of 7.70 kcal mol^−1^ and less preferred transition state geometry in TS_SR1_ compared to (*S*)-**1** insertion. Note that the chirality of methyl phenyllactate (**5**)—that mimics the chain end in polymerization—can affect the orientation of **1** approaching the Zn center (Supplementary Fig. 17). In the computed ring opening reaction of **1** mediated by (**NNO-1**)Zn/(*R*)-**5**, (*S*)-**1** sterically interacts with the *tert*-butyl group on **NNO-1** ligand that results in a less favored geometry (Supplementary Fig. 17). Such steric effects may thus be a key factor responsible for the observed isoselecitivity in (**NNO-1**)Zn–mediated ROP of *rac*-**1**, in accord with the chain-end-controlled mechanism.

On the basis of the above-described results, we propose a stereocontrol mechanism that accounts for the formation of the stereoblock microstructure (Fig. 3d). The ring-opening event is the reaction of (**NNO-1**)Zn/BnOH complex separately with l-**1** and d-**1** at similar reaction rates. The isospecific chain propagation occurs owing to the modestly higher activation energy of reaction with the disfavored OCA stereoisomer based on the computation results (step A). A stereoerror occurs and the other enantiomer is incorporated (step B) and enchained (step C). Because the rate of stereoerror occurrence is slower than the rate of chain propagation, the reaction generates a stereoblock microstructure with alternating blocks of enantiomers. Notably, because the two enantiomers have similar polymerization rate constants (Supplementary Fig. 11c) and because the obtained polymer has a narrow *Đ*, the formation of dormant chains during polymerization, which is often observed in the chain-shuttling mechanism, is unlikely to have occurred^48, 59–61^.

The synthesis of stereoblock copolymers, including polyolefins, polylactides, and polycarbonates, has been reported^5, 19, 23, 30, 32, 53, 59, 61–66^. Notably, stereoblock polylactide has been synthesized via enantiomorphic site control by using racemic chiral aluminum catalysts; the average block length is 11 lactide monomer units^19^,which is lower than the block length reported in the current study. Racemic bimetallic cobalt and chromium catalysts have been used for the synthesis of stereoblock polycarbonate via a chain-shuttling mechanism^48, 61^. To our knowledge, ours is the first study to show that an achiral catalyst can mediate the synthesis of high-MW stereoblock polyesters ( > 70 kDa) via the chain-end-control mechanism.

### Synthesis of gradient copolymers

**Fig. 5:**
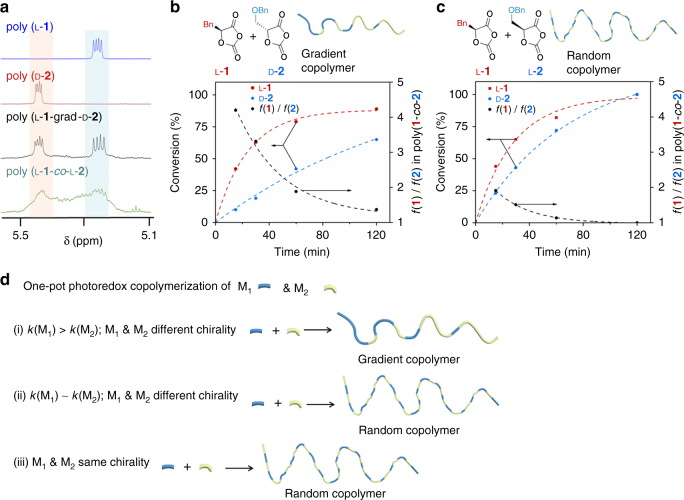
One-pot synthesis of gradient copolymers by copolymerization. **a** Comparison of the ^1^H NMR spectra of the α-methine regions of poly(l-**1**-*grad*-d-**2**) and poly(l-**1**-*co*-l-**2**) with those of poly(l-**1**) and poly(d-**2**). All photoredox polymerizations were mediated by (**NNO-1**)ZnEt. **b** Kinetics of l-**1** (red) and d-**2** (blue) copolymerization to form a gradient copolymer (Table 3, entry 1). **c** Kinetics of l-**1** (red) and l-**2** (blue) copolymerization to form a random copolymer (Table 3, entry 2). **d** Summary of the relationship between copolymer microstructure, reaction kinetics, and OCA monomer chirality

Our successful synthesis of stereoblock polyester by means of (**NNO-1**)ZnEt-mediated photoredox ROP prompted us to determine whether this technique could be used to control the microstructure of copolymers. We began by investigating the one-pot photoredox ROP of l-**1** and d-**2** mediated by (**NNO-1**)ZnEt and Ni/Ir catalysts in the presence of BnOH ([l-**1**]/[d-**2**]/[Ni]/[Zn]/[BnOH]/[**Ir-1**] = 100/100/1/1/1/0.1). The monomers were consumed completely within 4 h, and a product with a *M*_n_ of 26.1 kDa (which is close to the expected MW) and a narrow *Đ* (1.03) was obtained (Table 3, entry 1).^1^H NMR spectroscopy proved that this polymer was highly isotactic, as evidence by the dominant *mmm* methine peaks in the spectra of both l-**1** (5.2 ppm) and d-**2** (5.4 ppm), which were assigned on the basis of comparison with the peaks for the corresponding homopolymers (Fig. 5a and Supplementary Fig. 18). The small peaks around these two peaks likely corresponded to mixed tetrad sequences of l-**1** and d-**2** (e.g., [l-**1**]-[l-**1**]-[d-**2**]-[d-**2**] and [d-**2**]-[l-**1**]-[d-**2**]-[d-**2]**; Fig. 5a).^1^H NMR spectroscopic analysis of the kinetics of copolymerization showed that l-**1** conversion was faster than d-**2** conversion. For instance, at 15 min, conversion of l-**1** was 42%, whereas that of d-**2** was only 10% ([l-**1**]/[d-**2**]/[Ni]/[Zn]/[BnOH]/[**Ir-1**] = 100/100/1/1/1/0.1; Fig. 5b). Additionally, the l-**1**/d-**2** ratio in the copolymer dramatically decreased from 4.2 at 15 min to 1.3 at 2 h, which indicates the formation of a gradient copolymer (Fig. 5b). These results suggest that l-**1** polymerized first and that a gradient copolymer was formed.

**Table 3:** Stereoselective controlled photoredox ring-opening polymerization of racemic *O*-carboxyanhydrides

Entry	Monomers	FR^a^	Time	Conv. (%)^b^	*M*_n_ (kDa)^c^	MW_cal_ (kDa)	*Ð* ^c^	Microstructure^d^
1	l-**1** / d-**2**	100/100	4	100	26.1	32.7	1.03	Gradient
2	l-**1** / l-**2**	100/100	4	100	23.5	32.7	1.02	Random
3	d-**1** / l-**2**	100/100	4	100	30.6	32.7	1.01	Gradient
4	l-**1** / d-**3**	100/100	6	100	25.3	36.9	1.04	Gradient
5	d-**1** / l-**3**	100/100	4	100	27.8	36.9	1.02	Gradient
6	l-**1** / d-**4**	200/200	4	100	43.3	44.1	1.03	Gradient
7	l-**2** / d-**3**	100/100	4	100	29.7	39.9	1.02	Gradient
8	l-**3** / d-**4**	200/200	4	100	37.4	58.5	1.03	Random
9	l-**1** / l-**3**	100/100	4	100	36.3	36.9	1.02	Random

FR, feeding ratio of [OCA]/[Zn]; Conv., monomer conversion; Temp., temperature; *M*_n_, number-average molecular weight; MW_cal_, molecular weight calculated from the ratio of the amount of monomer to the amount of catalyst; *Ð*, molecular weight distribution

^a^ FR is the ratio of the amount of monomer to the amount of catalyst

^b^ Determined from the intensity of the Fourier transform infrared peak at 1805 cm^−1^, which corresponds to the anhydride group of the OCA

^c^ Determined by gel-permeation chromatography

^d^ Determined by ^1^H and ^13^C NMR spectroscopy. See Supplementary Figs. 18-20, and Supplementary Figs. 22–27

In contrast, replacing d-**2** with l-**2** resulted in the formation of a random copolymer (Table 3, entry 2). The two methine peaks could not be distinguished in the ^1^H NMR spectrum (Fig. 5a and Supplementary Fig. 19). Analysis of the polymerization kinetics showed that the l-**1**/l-**2** ratio decreased from 1.9 at 15 min to 1.1 at 60 min, suggesting the formation of a random copolymer (Fig. 5c). Note that the l-**2** polymerization rate was almost identical to that of d-**2** (Supplementary Fig. 11c), indicating that the difference between the kinetics of the reactions of **1** and **2** did not determine whether poly(l-**1**-*grad*-d-**2**) (*grad*, gradient) or random poly(l-**1**-*co*-l-**2**) formed. The preference for enchainment of l-**2** over d-**2** at the l-**1** chain end was presumably due to chain-end control mediated by the (**NNO-1**)Zn–alkoxide complex. Note that reversing the chirality of the monomers, that is, using d-**1** and l-**2** instead of l-**1** and d-**2**, did not affect either the polymerization results or the microstructure of the obtained polymer (Table 3, entry 3; Supplementary Fig. 20).

The results of the copolymerization studies described herein indicate that comonomers with the same chirality can be readily incorporated at the chain end—that is, using monomers with the same chirality is more likely to result in stereoerrors than using monomers with opposite chirality, which results in copolymers with different microstructures. However, the reactivities of different OCA monomers could also affect the selectivity of the copolymerization. To elucidate the microstructures formed by copolymerization, we first evaluated the reactivities of OCA monomers **1–4** by means of kinetic studies (Supplementary Figs. 11a-c and 21). We found that their polymerization rates decreased in the order *k*(**1**) **>** *k*(**4**) ≈ *k*(**3**) **>** *k*(**2**) (Supplementary Figs. 11c and 21f; for example, at a [OCA]/[Zn] ratio of 300, *k*(l-**1**)/*k*(l-**4**)/*k*(l-**3**)/*k*(l-**2**) = 223.9/5.7/2.7/1), and that there was no obvious difference between the reaction rates of the two enantiomers of a given monomer (Supplementary Fig. 21b).

We then carried out one-pot copolymerizations of various pairs of enantiomers of **1**–**4**. Copolymerizations of l-**1** and d-**3**, d-**1** and l-**3**, l-**1** and d-**4**, and l-**2** and d-**3** resulted in the formation of gradient copolymers with *M*_n_ values close to the calculated MWs and *Ð* values of < 1.1, as evidenced by the distinct and well-split peaks in their ^1^H and ^13^C NMR spectra (Table 3, entries 4–7; Supplementary Figs. 22–25). However, copolymerization of l-**3** and d-**4** afforded a random copolymer, as indicated by the broad peaks in the ^1^H NMR spectrum (entry 8; Supplementary Fig. 26 and Fig. 5d (ii)). Notably, polymerization of l-**1** and l-**3**, which have the same chirality, also resulted in a random copolymer (see entry 9, as well as entry 2; Supplementary Fig. 27 and Fig. 5d (iii)). These results support our observation that the use of comonomers with opposite chirality was required for one-pot synthesis of gradient copolymers mediated by the (**NNO-1**)Zn complex (Fig. 5d).

## Discussion

We discovered that a (**NNO-1**)Zn complex can be used for photoredox ROP to prepare high-MW stereoblock copolymers from racemic OCAs in a rapid, controlled manner, as indicated by kinetic studies using deuterated monomer and by NMR spectroscopy. The obtained stereoblock copolymers, for example, poly(*sb*-**1**), were highly isotactic with unusually long sequence numbers, and they displayed distinct *T*_m_ values around 170 °C. Quantum chemical simulations suggest that the stereoselective step of the polymerization can be explained by steric interactions between the chiral OCA monomer and salicylidene moiety of the Zn catalyst, suggesting a ligand-assisted chain-end-control mechanism. Furthermore, in our one-pot photoredox copolymerization of two different OCA monomers, the use of (**NNO-1**)Zn-mediated kinetic resolution of the comonomers during enchainment and showed a chirality preference that allowed for the synthesis of gradient copolymers. These results are significant because they offer new routes to polyesters with pendant side-chain functionalities and various microstructures. To our knowledge, no photoredox stereoselective polymerization has been reported before. The synergistic use of photoredox living polymerization and stereoselective catalysts can be suggestive to prepare many other stereoregular polymers. Future work will focus on the design of polymerization catalysts, as well as exploration of their mechanistic complexity, with the goal of achieving previously unattainable microstructural control of polyesters, which could in turn result in the preparation of polymers with new physiochemical properties suitable for a wide range of applications.

## Methods

### General

All manipulations involving air- and water-sensitive compounds were carried out in a glove box. Bis(1,5-cyclooctadiene) nickel(0) (Ni(COD)_2_), 2,2′-bipyridine (bpy) and **Ir-1** were purchased from Strem Chemicals (Newburyport, MA, U.S.). Anhydrous tetrahydrofuran (THF) was dried by alumina columns and stored with 4 Å molecular sieve in the glove box. Anhydrous THF-*d*_8_, BnOH, hexane, diethyl ether, and diisopropyl ether were purchased from Sigma-Aldrich (St. Louis, MO, U.S.) dried by 4 Å molecular sieves and stored in the glove box. All OCA monomers were synthesized following the literature^39, 41, 67–69^, recrystallized three times and stored in a −30 °C freezer in the glove box. (**NNO-*n***)ZnEt complexes were prepared following the literature^45, 70^ and stored in a −30 °C freezer in the glove box. All other chemicals were purchased from Sigma-Aldrich or noted in Supplementary Methods.

### Photoredox polymerization of *rac*-1 to synthesize stereoblock copolymer poly(*sb*-1)

In a glove box, prior to polymerization, all reagents were cooled at −20–30 ^o^C in the cold trap equipped with a thermometer. The cold trap was cooled by liquid nitrogen and ethanol in a dewar outside the glove box^41^. l-**1** (10 mg, 100 μL of 100 mg/mL, 0.052 mmol, 150 equiv.) and d-**1** (10 mg, 100 μL of 100 mg/mL, 0.052 mmol, 150 equiv.) was mixed with (bpy)Ni(COD) (25.8 μL THF solution, 0.347 μmol, 1 equiv.), (**NNO-1**)ZnEt (38.8 μL of 3.7 mg/mL in THF, 0.347 μmol, 1 equiv.), BnOH (19.8 μL of 1.9 mg/mL in THF, 0.347 μmol, 1 equiv.) and **Ir-1** (40.3 μL of 0.97 mg/mL in THF, 0.0347 μmol, 0.1 equiv.) in a 7-mL glass vial equipped with a magnetic stir bar at −20 ^o^C. The solution was stirred and irradiated with a 34 W blue LED lamp (Kessil KSH150B LED Grow Light 150) at −15 ± 5 °C (with a cooling fan to keep the reaction temperature) over 4–6 h. The OCA monomer conversion was monitored by Fourier-transformed infrared spectrometry. The resulting polymer’s MW and *Ð* were directly measured by gel-permeation chromatography after the polymerization. The characterization of the obtained polymers and kinetic studies are documented in Supplementary Methods.

### Data availability

The authors declare that all data supporting the current findings of this study are available in the main manuscript or in the Supplementary information. Other data are available from the corresponding author on reasonable request.

## Electronic supplementary material

Supplementary Information

Description of Additional Supplementary Files

Supplementary Data 1
