# Supplementary material for: Stereoselective photoredox ring-opening polymerization of O-carboxyanhydrides
Source: Nat Commun. 2018 Apr 19;9:1559. doi: 10.1038/s41467-018-03879-5 (PMC5908805; doi:10.1038/s41467-018-03879-5)
Supplement: Supplementary file 1 — Supplementary Information [file 41467_2018_3879_MOESM1_ESM.pdf]

Stereoselective Photoredox Ring-Opening Polymerization of *O*-Carboxyanhydrides

Tong et al.

Supplementary Information

Supplementary Methods

Materials

General

Bis(1,5-cyclooctadiene) nickel(0) ($Ni(COD)_2$), 2,2'-bipyridine (bpy) and bis[2-(2,4-difluorophenyl)-5-(trifluoromethyl)pyridine] iridium(III) hexafluorophosphate (**Ir-1**) were purchased from Strem Chemicals (Newburyport, MA). *O*-benzyl-L-serine, *O*-benzyl-D-serine, L-glutamic acid-γ-benzyl ester and D-glutamic acid-γ-benzyl ester were purchased from Chem-Impex (Wood Dale, IL). L-phenylalanine and D-phenylalanine was purchased from Alfa Aesar (Haverhill, MA). Anhydrous tetrahydrofuran (THF) was dried by alumina columns and stored with 4Å molecular sieve in the dark bottle in the glove box. Anhydrous THF-d_8, benzyl alcohol, hexane, diethyl ether, diisopropyl ether and dichloromethane were dried by 4Å molecular sieves and stored in the glove box. All other chemicals were purchased from Sigma-Aldrich (St. Louis, MO) unless otherwise noted.

OCA monomers

L-PheOCA (L-**1**) and D-PheOCA (D-**1**),[1] L-Ser(Bn)OCA (L-**2**) and D-Ser(Bn)OCA (D-**2**),[2] L-Glu(Cbz)OCA (L-**3**) and D-Glu(Cbz)OCA (D-**3**),[3] and L-LacOCA (L-**4**) and D-LacOCA (D-**4**)[4] were synthesized and recrystallized according to the literature. All OCA monomers were recrystallized three times and stored in -30 °C freezer in the glove box.

Zn catalysts

(**NNO-1**)ZnEt, (**NNO-2**)ZnEt and (**NNO-3**)ZnEt were prepared following the literatures.[5, 6] $Zn(HMDS)_2$ was prepared and distilled according to the literature.[7] NNO-4 ligand and (NNO-4)ZnEt was synthesized using modified procedures.[5] All Zn complex were stored in the glove box freezer (-30 °C).

Synthesis of (NNO-4)ZnEt

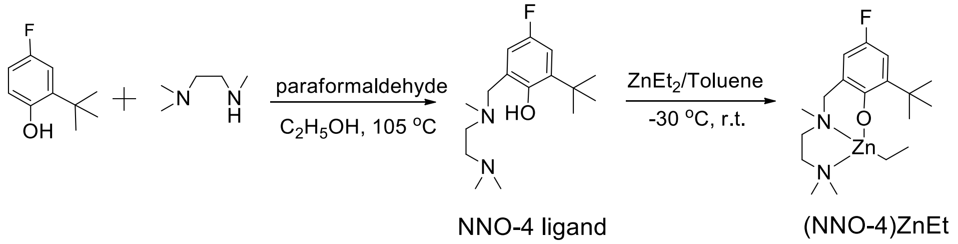

NNO-4 ligand: A solution of *N*,*N*,*N'*-trimethylethylenediamine (1.13 g, 11.0 mmol), paraformaldehyde (1.0 g, 33.6 mmol), and 2-(*tert*-butyl)-4-fluorophenol[8] (1.69 g, 10.0 mmol) in ethanol (12 mL) was heated at reflux under nitrogen for 14 h. The solution was cooled and then HBr (3.0 mL, 54.7 mmol) was added, and the solution was neutralized with saturated $NaHCO_3$ solution. The solution was washed with CH_2Cl_2 (3 × 50 mL) and dried over Na_2SO_4, the solvent was removed under vacuum, and the resulting material was purified using column chromatography (20:1, CH_2Cl_2: MeOH) to yield a light yellow oil, NNO-4 ligand (78% yield). ^{1}H NMR (400 MHz, $CDCl_3$): δ 6.89 (dd, J_1 = 3.12 Hz, J_2 = 11.04 Hz, 1H), 6.55 (dd, J_1 = 3.12 Hz, J_2 = 8.04 Hz, 1H), 3.62 (s, 2H), 2.57 (m, 2H), 2.46 (m, 2H), 2.30 (s, 3H), 2.20 (s, 6H), 1.38 (s, 9H). ^{13}C NMR (100 MHz, $CDCl_3$): δ 156.69, 154.36, 152.81, 152.80, 138.34, 138.28, 123.13, 123.05, 112.87, 112.84, 112.82, 112.80, 112.76, 112.65, 112.58, 112.56, 112.53, 61.50, 61.35, 61.22, 57.18, 57.03, 54.29, 45.92, 45.82, 45.72, 45.62, 41.96, 41.84, 34.93, 29.52, 29.41, 29.31, 29.20.

(NNO-4)ZnEt: NNO-4 ligand (957mg, 3.39 mmol) was dissolved in toulene (3 mL), and the solution was cooled to -30 °C. Diethyl zinc toluene solution (3.660 mL, 4.07 mmol) was added to this solution slowly, and the mixture was stirred overnight at room temperature. The solvent was removed under vacuum. The solid was washed with hexane (3 × 3 mL), recrystallization from toluene, and dried under vacuum (54% yield). ^{1}H NMR (400 MHz, C_6D_6): δ 7.30 (dd, J_1 = 3.36 Hz, J_2 = 11.24 Hz, 1H), 6.60 (dd, J_1 = 3.32 Hz, J_2 = 8.16 Hz, 1H), 3.09 (d, 1H, J = 12 Hz), 2.92 (d, 1H, J = 12 Hz), 1.96 (m, 1H), 1.82 (s, 3H), 1.73 (m, 16H), 1.58 (t, 3H, J = 8.12 Hz), 1.51 (m, 2H), 0.41 (m, 2H). ^{13}C NMR (100 MHz, C_6D_6): δ 163.65, 163.64, 153.94, 151.67, 140.16, 140.11, 122.58, 122.51, 114.76, 114.56, 114.55, 114.33, 61.20, 61.18, 56.89, 52.38, 46.62, 45.57, 44.82, 35.70, 35.69, 29.83, 13.92, -3.99.

Ni catalysts

The Ni complex solution was prepared freshly prior to the reaction. In a glove box, $Ni(COD)_2$ (5.5 mg, 0.02 mmol) was mixed with bipyridyl ligand (e.g., bpy-1, 3.1 mg, 0.02 mmol) or or tricyclophosphine (11.2 mg, 0.04 mmol) in THF solution (300 μL) at room temperature and stirred for 1-2 hour to ensure all $Ni(COD)_2$ dissolved. The Ni complex solution could be stored in the glove box freezer (-30 °C) no longer than seven days.

Instrument and Characterization

NMR

All room temperature NMR and homodecoupling ^1H NMR spectra were recorded on Agilent U4-DD2 (400 MHz) or Bruker Avance II (500 MHz). Low temperature ^1H and ^{13}C NMR spectra were measured on Bruker Avance III (600 MHz) after the inner temperature of the NMR machine reached -20 ^oC for 20 min. The samples were kept at -20 ^oC ± 5 ^oC with a dry ice/ethylene glycol bath before the low-temperature NMR acquisition.

FTIR

Fourier-transform infrared spectra were recorded on an Agilent Cary 630 FT-IR spectrometer (Agilent Technologies Inc., Santa Clara, CA, USA) equipped with Diamond ATR and transmission sampling accessory.

Monomer conversion measurement:

A small aliquot of polymer solution (20 µL) was removed out of the glove box and quenched with 5% acetic acid / THF solution (20 µL). The mixture (~10 µL) was immediately dropped onto the FTIR-ATR diamond sampler and formed a film within 10-20 seconds for the spectra measurement. The peak at 1800 cm^{-1} is assigned as the anhydride bond stretch in OCA; the peak at 1760 cm^{-1} corresponds to the formation of the ester bond in the polymer. The monomer conversion was determined by the intensity ratio between 1760 cm^{-1} and 1800 cm^{-1}: conversion% = $I_{1760} / (I_{1760} + I_{1800})$.[1, 9]

Gel permeation chromatography (GPC)

GPC experiments were performed on a system equipped with an isocratic pump with degasser (Agilent 1260 series, Agilent Technologies, Santa Clara, CA, USA), Wyatt DAWN HELEOS multiangle laser light scattering (MALS) detector (GaAs 30 mW laser at λ=690nm), and an Wyatt Optilab rEX differential refractive index (DRI) detector with a 690 nm light source (Wyatt Technology, Santa Barbara, CA, USA). Separations were performed using serially connected size exclusion columns (100 Å, 500 Å, 10^3 Å, and 10^4 Å Phenogel columns, 5 µm, 300 × 4.6 mm, Phenomenex, Torrance, CA, USA) at 35 °C using THF as the mobile phase with a flow rate of 0.35 mL/min. The polymer molecular weight (MW) and molecular weight distribution ($Đ$) were determined using Zimm model fit of MALS-DRI data by ASTRA software (Version 6.1, Wyatt Technology).

The refractive index increment dn/dc value was determined by the Wyatt Optilab rEX refractive index detector using ASTRA software dn/dc template (Version 6.1, Wyatt Technology). Five polymer / THF solutions with different concentrations were sequentially injected into the refractive index detector and the refractive index values were plotted versus concentration in ASTRA software. The slope of the linear fitting data is the dn/dc value.

The dn/dc values: poly(L-**1**), 0.1805; poly(L-**2**), 0.1377; poly(L-**3**), 0.1057; poly(L-**4**), 0.042.

TGA and DSC

Differential scanning calorimetry (DSC) measurements were performed on TA Instruments DSC Q2000 instrument equipped with photocalorimeter accessory and RCS90 cooling system. Polymer samples in crimped aluminum pans were analyzed under nitrogen at a heating rate of 10 °C/min from 20 to 200 °C. Glass transition temperature (T_g) and melting temperature (T_m) were obtained and reported from the second heating run.

Polymerization Procedures

The photoredox polymerization of L-1 initiated by (bpy)Ni(COD) / (NNO-1)ZnEt / BnOH / Ir-1

In a glove box, prior to the polymerization, all reagents were cooled in the cold trap equipped with a thermometer at -20-30 ^oC, which was cooled by the liquid nitrogen and ethanol in dewar. The monomer **1** (233 μL of 100 mg/mL in THF, 0.121 mmol, 300 equiv.) was mixed with (bpy)Ni(COD) (30.1 μL THF solution, 0.405 μmol, 1 equiv.), (**NNO-1**)ZnEt (45.3 μL of 3.7 mg/mL in THF, 0.405 μmol, 1 equiv.), BnOH (23.0 μL of 1.9 mg/mL in THF, 0.405 μmol, 1 equiv.) and **Ir-1** (46.9 μL of 0.97 mg/mL in THF, 0.0405 μmol, 0.1 equiv.) in a 7-mL glass vial equipped with a magnetic stir bar. The solution was stirred and irradiated with a 34 W blue LED lamp (Kessil KSH150B LED Grow Light 150) at -15 ^oC ± 5 ^oC (with a cooling fan to keep the reaction temperature) over 4 hours. The OCA monomer conversion was monitored by FTIR. The resulted polymer's MW and *Đ* were directly measured by GPC after the polymerization.

The photoredox polymerization of racemic L-1 and D-1 initiated by (bpy)Ni(COD) / (NNO-1)ZnEt / BnOH / Ir-1

In a glove box, prior to the polymerization, all reagents were cooled in the cold trap equipped with a thermometer at -20-30 ^{o}C, which was cooled by the liquid nitrogen and ethanol in dewar. L-**1** (10 mg, 100 μL of 100 mg/mL, 0.052 mmol, 150 equiv.) and D-**1** (10 mg, 100 μL of 100 mg/mL, 0.052 mmol, 150 equiv.) was mixed with (bpy)Ni(COD) (25.8 μL THF solution, 0.347 μmol, 1 equiv.), (**NNO-1**)ZnEt (38.8 μL of 3.7 mg/mL in THF, 0.347 μmol, 1 equiv.), BnOH (19.8 μL of 1.9 mg/mL in THF, 0.347 μmol, 1 equiv.) and **Ir-1** (40.3 μL of 0.97 mg/mL in THF, 0.0347 μmol, 0.1 equiv.) in a 7-mL glass vial equipped with a magnetic stir bar at -20 ^{o}C. The solution was stirred and irradiated with a 34 W blue LED lamp (Kessil KSH150B LED Grow Light 150) at -15 ^{o}C ± 5 ^{o}C (with a cooling fan to keep the reaction temperature) over 4-6 hours. The OCA monomer conversion was monitored by FTIR. The resulted polymer's MW and *Đ* were directly measured by GPC after the polymerization.

The photoredox copolymerization of L-1 and D-2 initiated by (bpy)Ni(COD) / (NNO-1)ZnEt / BnOH / Ir-1

In a glove box, prior to the polymerization, all reagents were cooled in the cold trap equipped with a thermometer at -20-30 ^{o}C, which was cooled by the liquid nitrogen and ethanol in the dewar. L-**1** (10 mg, 100 μL of 100 mg/mL, 0.052 mmol, 100 equiv.) and D-**2** (11.6 mg, 115.6 μL of 100 mg/mL, 0.052 mmol, 100 equiv.) was mixed with (bpy)Ni(COD) (38.7 μL THF solution, 0.521 μmol, 1 equiv.), (**NNO-1**)ZnEt (58.3 μL of 3.7 mg/mL in THF, 0.521 μmol, 1 equiv.), BnOH (29.6 μL of 1.9 mg/mL in THF, 0.521 μmol, 1 equiv.) and **Ir-1** (60.4 μL of 0.97 mg/mL in THF, 0.052 μmol, 0.1 equiv.) in a 7-mL glass vial equipped with a magnetic stir bar at -20 ^{o}C. The solution was stirred and irradiated with a 34 W blue LED lamp (Kessil KSH150B LED Grow Light 150) at -15 ^{o}C ± 5 ^{o}C (with a cooling fan to keep the reaction temperature) over 4-8 hours. The OCA monomer conversion was monitored by FTIR. The resulted polymer's MW and *Đ* were directly measured by GPC after the polymerization.

Other ring-opening polymerization techniques

The ring-opening polymerizations of OCAs initiated by DMAP/BnOH or (bpy)Ni(COD) / $Zn(HMDS)_2$ / BnOH / **Ir-1** were reported in our previous work.[9]

Kinetic study of the photoredox polymerization of OCA

In a glove box, the monomer L-**1** or D-**1** (300 μL of 33.3 mg/mL in THF, 52.1 μmol, 600 equiv.) was mixed with (bpy)Ni(COD) (15.4 μL THF solution, 86.8 nmol, 1 equiv.), (**NNO-1**)ZnEt

(19.4 μL of 1.85 mg/mL in THF, 86.8 nmol, 1 equiv.), BnOH (12.5 μL of 0.75 mg/mL in THF, 86.8 nmol, 1 equiv.) and **Ir-1** (10.1 μL of 0.967 mg/mL in THF, 8.68 nmol, 0.1 equiv.) in a 7-mL glass vial equipped with a magnetic stir bar. The mixture was stirred at -15 ^{o}C ± 5 ^{o}C with irradiation in a manner similar to that in **S3.2**. At the predetermined time point, 20 μL of the polymer solution was taken out and immediately analyzed by FTIR. All the catalysts' concentrations were varied, and the semi-logarithmic plots were drawn to calculate the kinetic constants and reaction orders according to the kinetic laws:

$-d[\mathbf{1}]/dt = k_{app} \cdot [\mathbf{1}]$ (eq.1)

where k_{app} is the apparent first-order rate constant. For eq.1 it equals to

$\ln[\mathbf{1}]_0/\ln[\mathbf{1}]_t = k_{app} \cdot t + C$ (eq.2)

thus $\ln[\mathbf{1}]_0/\ln[\mathbf{1}]_t$ versus t was plotted.

Supplementary Table 1. NMR analysis of microstructures of poly(*rac*-OCA).[a]

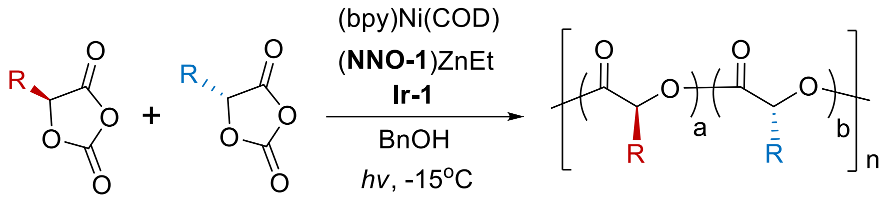

Monomers	Condition	Homodecoupling ^{1}H NMR methine						^{13}C NMR methine	
		mmm	*mmr*	*rmm*	*mrm*	*mrr/rrm*	P_m	*mmm*	P_m
L-**1**/D-**1**	as shown	0.91	0.03	0.02	0.02	0.01	0.97	0.92	0.97
L-**1**/D-**1**	(**NNO-2**)Zn replacing (**NNO-1**)Zn	0.79	0.09	0.06	0.04	0.01	0.93	0.83	0.94
L-**1**/D-**1**	[L-**1**]/[D-**1**] = 200/100	0.82	0.06	0.06	0.05	0.01	0.93	0.81	0.93
L-**1**/D-**1**	only (**NNO-1**)Zn/BnOH	0.61	0.10	0.10	0.25	0.04	0.85	0.72	0.89
L-**1**/D-**1**	[L-**1**]/[D-**1**]/[Ni]/[Zn] = 300/300/2/1	0.82	0.06	0.03	0.06	0.03	0.93	0.86	0.95
L-**2**/D-**2**	as shown	0.69	0.21	0	0.08	0.01	0.88	0.78 [b]	0.88
L-**3**/D-**3** [c]	as shown	-	-	-	-	-	-	0.83	0.94
L-**4**/D-**4** [d]	as shown	0.77	0.05	0.08	0.08	0	0.92	0.79	0.92

[a] The assignment of triads and tetrads follows the literature.[10]

[b] The assignment in ^{13}C NMR is triad *mm* instead of tetrad *mmm* (see Supplementary Figure 8).

[c] The α-methine peaks are overlapped with CH_2 peaks in Cbz groups in ^{1}H NMR spectrum, preventing from the homodecoupling analysis (see Supplementary Figure 9). The P_m of poly(*rac*-**3**) was determined by ^{13}C NMR spectrum.

[d] The integration of the tetrad *rmr* is 0.02 (see Supplementary Figure 10).

Supplementary Table 2. Thermal properties of polymers with various microstructures.[a]

Entry	Polymer	M_n (kDa) [b]	$Đ$ [b]	T_g (°C) [c]	T_m (°C) [c]
1	poly(*sb*-**1**)	78.0	1.04	50	172
2	poly(L-**1**)	47.2	1.01	50	-
3	poly(*rac*-**1**) [d]	78.2	1.16	51	-
4	poly(L-**1**-*b*-D-**1**)	45.7	1.05	49	-
5	poly(L-**1**) + poly(D-**1**)	35.5	1.02	50	178
6	poly(*sb*-**4**)	12.4	1.04	52	162
7	poly(L-**4**)	12.0	1.05	-	169
8	poly(*rac*-**4**) [e]	7.8	1.16	50	-

[a] Abbreviation: M_n, number-average molecular weight; $Đ$, molecular weight distribution; T_g, glass transition temperature; T_m, melting temperature. All polymers were synthesized by photoredox ROP using (**NNO-1**)ZnEt (Table 2) unless otherwise noted. All dynamic scanning calorimetry thermograms were shown in Supplementary Figure 13.

[b] Determined by gel-permeation chromatography.

[c] Determined by dynamic scanning calorimetry.

[d] The polymer was catalyzed by $Zn(HMDS)_2$ instead of (**NNO-1**)ZnEt (Table 2, entry 6).

[e] The polymer was catalyzed by DMAP/BnOH instead of (**NNO-1**)ZnEt /(bpy)Ni(COD)/BnOH/**Ir-1** with light.

Supplementary Table 3. Effects of Ni complex ligand on ring-opening polymerization of rac-**1**.[a]

Entry	Ni complex	Conv. (%) [b]	M_n (kDa) [c]	MW_{cal} (kDa)	$Đ$ [c]
1	(bpy)Ni(COD)	100	27.8	29.7	1.01
2	$(PPh_3)_2$Ni(COD)	100	32.2	29.7	1.02
3	$(PCy_3)_2$Ni(COD)	72	25.3	29.7	1.01

[a] Reactions were conducted at an [L-**1**]/[D-**1**]/[Ni complex]/[(**NNO-1**)ZnEt]/[BnOH]/[**Ir-1**] ratio of 100/100/1/1/1/0.1 at -15 °C with light irradiation for 4 h. Abbreviations: Conv., monomer conversion; M_n, number-average molecular weight; MW_{cal}, molecular weight calculated on the basis of feeding ratio; $Đ$, molecular weight distribution.

[b] Determined from the intensity of the Fourier transform infrared peak at 1805 cm^{-1}, which corresponds to the OCA anhydride group.

[c] Determined by gel-permeation chromatography.

Supplementary Table 4. Gel-permeation chromatography analysis of poly(*sb*-**1**) with different FRs.[a]

Entry	FR [b]	Time	Conv. (%) [c]	M_n (kDa) [d]	MW_{cal} (kDa)	$Đ$ [d]
1	100/100	4	100	27.8	29.7	1.01
2	150/150	4	100	45.7	44.5	1.02
3	200/200	4	100	63.0	59.3	1.04
4	250/250	4	100	70.2	74.1	1.02
5	300/300	7	100	78.0	88.9	1.04

[a] Reactions were conducted under a standard photoredox polymerization condition. Conv., monomer conversion; M_n, number-average molecular weight; MW_{cal}, molecular weight calculated on the basis of FR; $Đ$, molecular weight distribution.

[b] FR refers to the ratio of the amount of monomer to the amount of the Zn catalyst.

[c] Determined from the intensity of the Fourier transform infrared peak at 1805 cm^{-1}, which corresponds to the OCA anhydride group.

[d] Determined by gel-permeation chromatography.

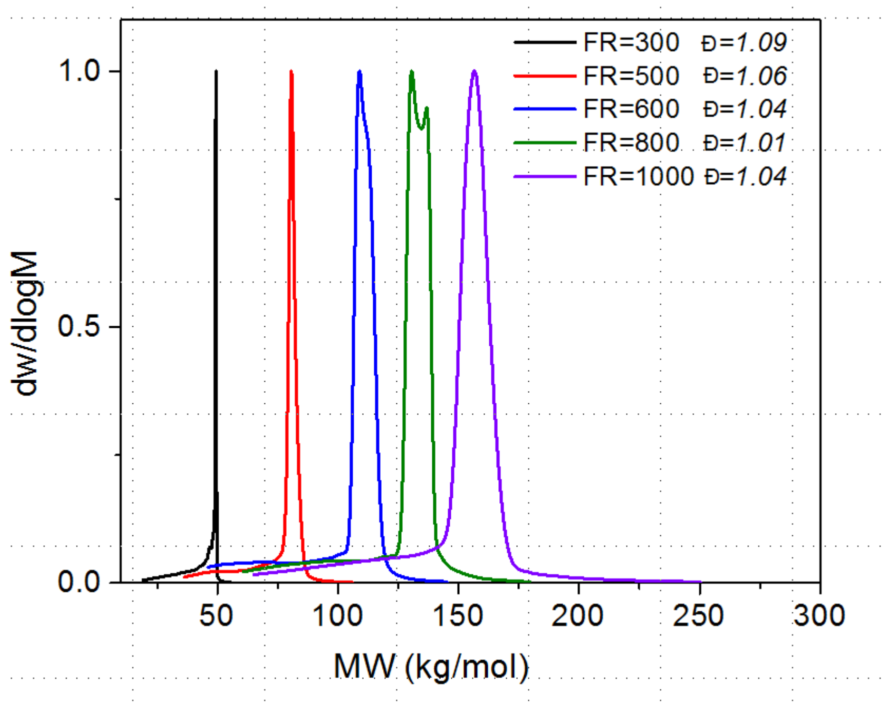

Supplementary Figure 1. GPC overlays of poly(L-**1**) at different FRs as shown in Figure 2b.

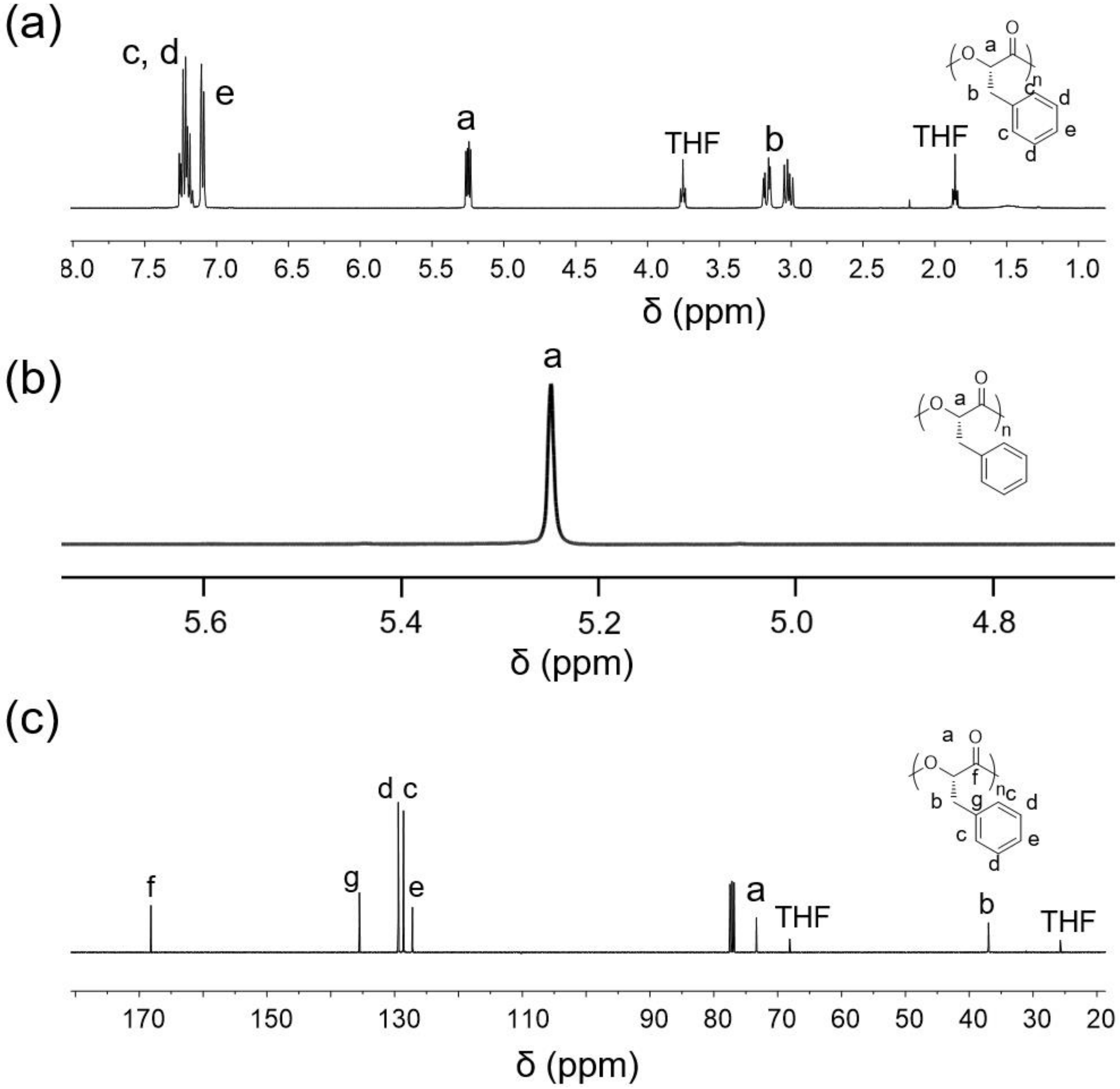

Supplementary Figure 2. NMR spectra of poly(L-**1**) in $CDCl_3$ (Table 1, entry 5). (a) ^{1}H NMR spectrum; (b) Homodecoupling ^{1}H NMR spectrum; (c) ^{13}C NMR spectrum.

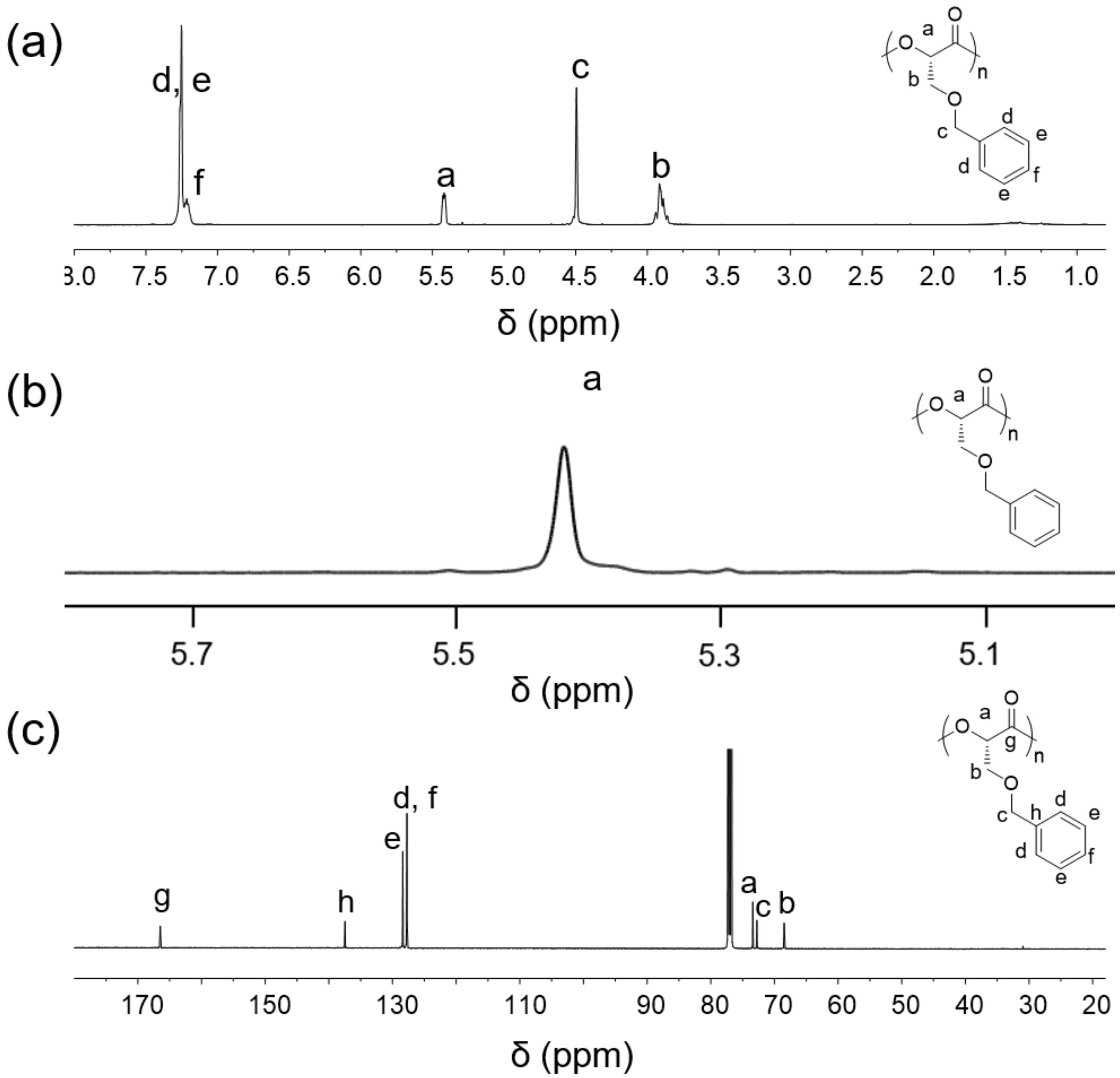

Supplementary Figure 3. NMR spectra of poly(L-**2**) in $CDCl_3$ (Table 1, entry 6). (a) ^1H NMR spectrum; (b) Homodecoupling ^1H NMR spectrum; (c) ^{13}C NMR spectrum.

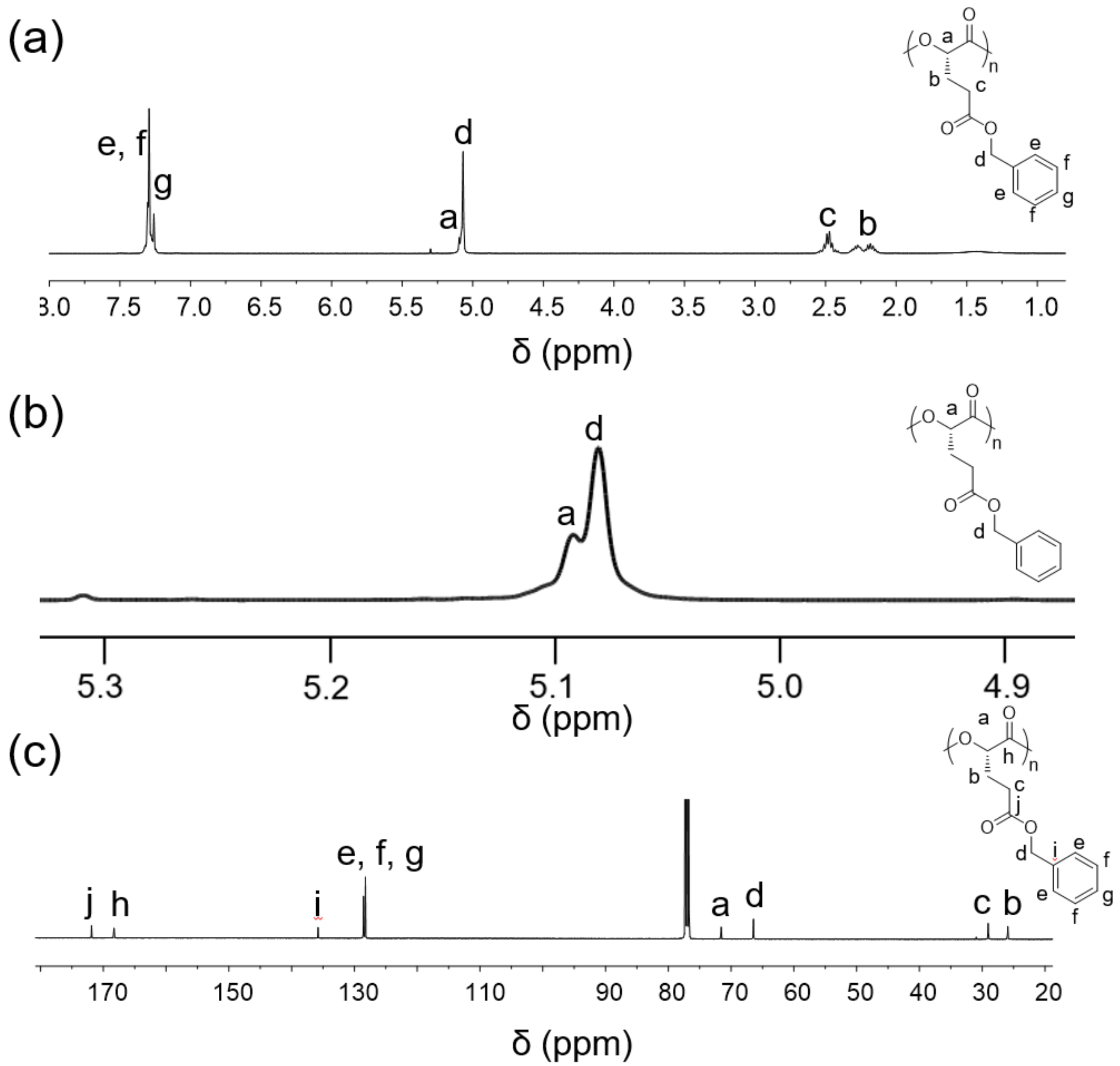

Supplementary Figure 4. NMR spectra of poly(L-**3**) in $CDCl_3$ (Table 1, entry 7). (a) ^1H NMR spectrum; (b) Homodecoupling ^1H NMR spectrum; (c) ^{13}C NMR spectrum.

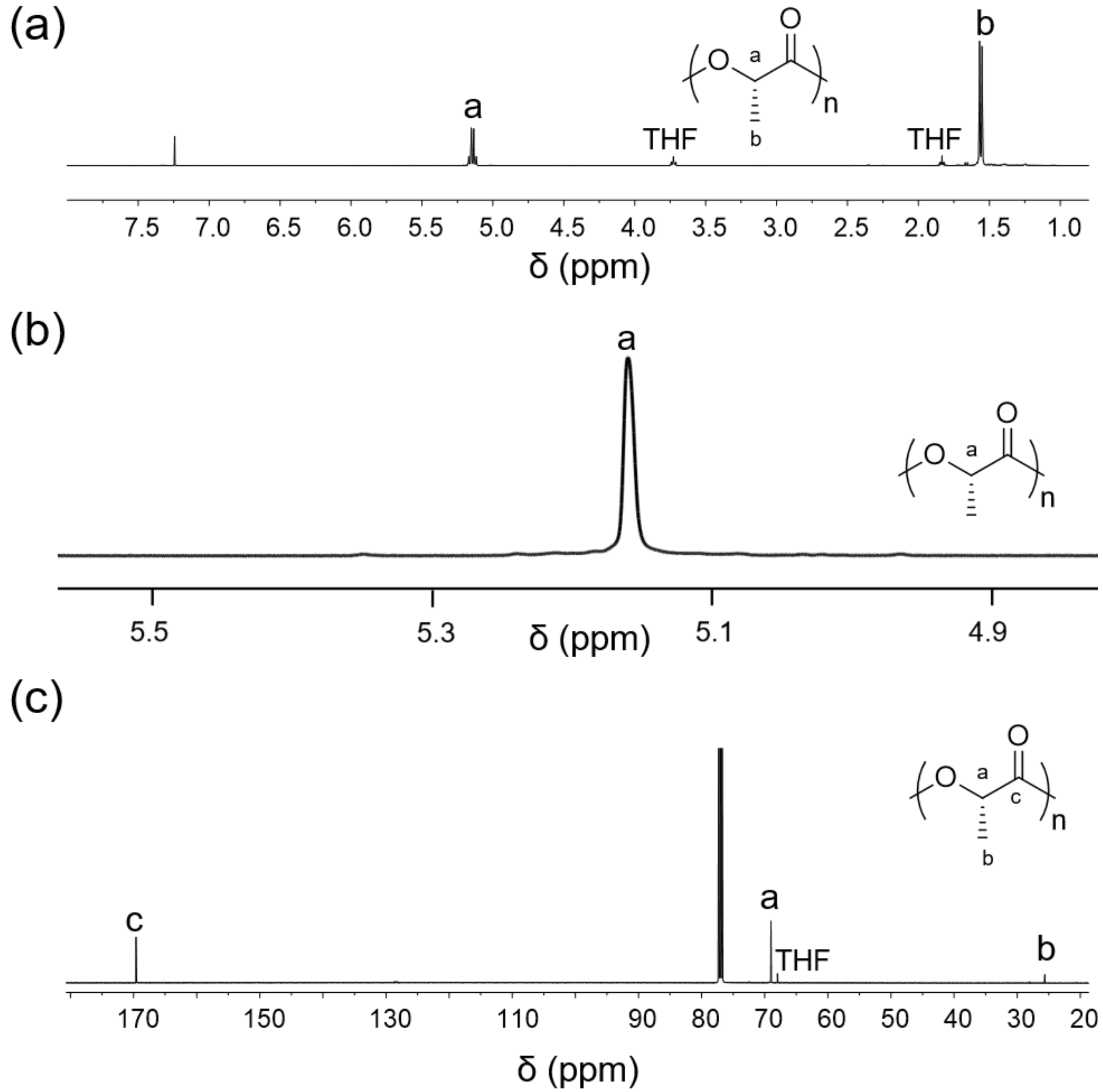

Supplementary Figure 5. NMR spectra of poly(L-**4**) in $CDCl_3$ (Table 1, entry 8). (a) ^1H NMR spectrum; (b) Homodecoupling ^1H NMR spectrum; (c) ^{13}C NMR spectrum.

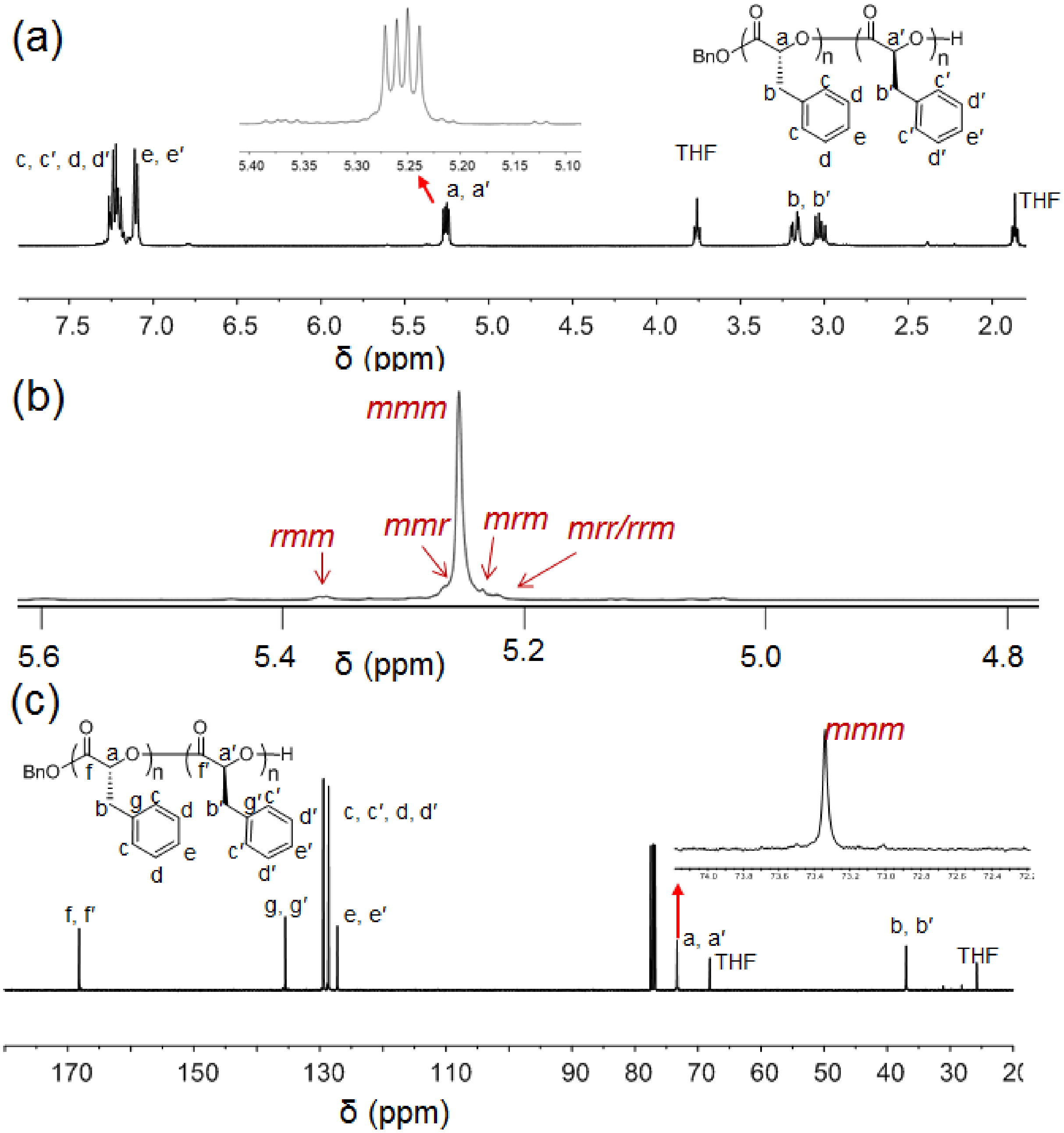

Supplementary Figure 6. NMR spectra of poly(*sb*-**1**) in $CDCl_3$ (Table 2, entry 1). (a) ^{1}H NMR spectrum; (b) ^{1}H homodecoupling NMR spectrum; (c) ^{13}C NMR spectrum. The assignment of homodecoupling ^{1}H NMR spectrum for all polymers was referred to the literature.[10]

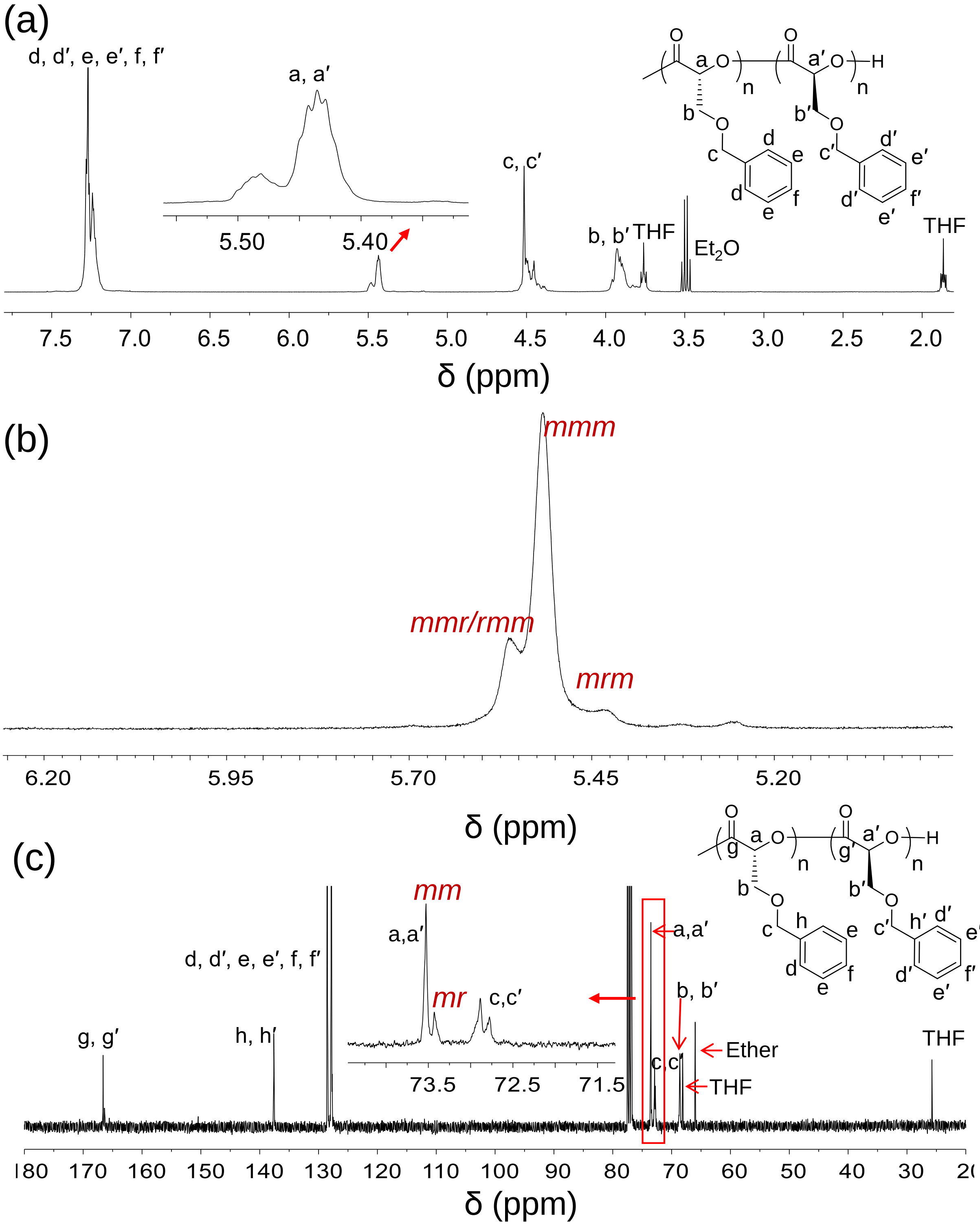

Supplementary Figure 7. NMR spectra of poly(*sb*-**2**) in $CDCl_3$ (Table 2, entry 3). (a) ^1H NMR spectrum; (b) ^1H homodecoupling NMR spectrum; (c) ^{13}C NMR spectrum.

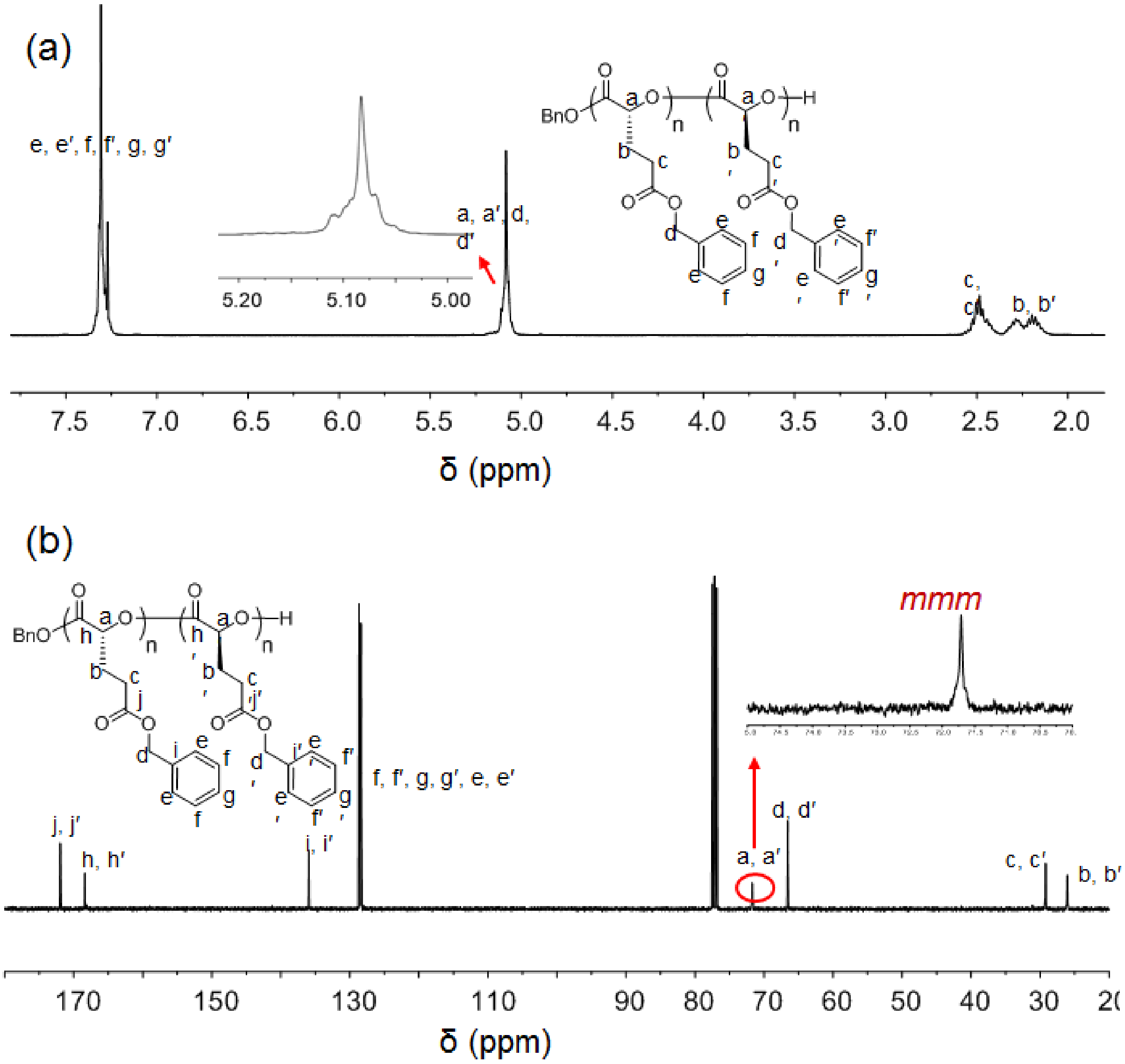

Supplementary Figure 8. NMR spectra of poly(*sb*-**3**) in $CDCl_3$ (Table 2, entry 4). (a) ^{1}H NMR spectrum; (b) ^{13}C NMR spectrum. Note the α-methine peaks *a* and *a'* are overlapped with peaks *d* and *d'* in ^{1}H NMR spectrum, preventing from the homodecoupling analysis. The P_m of poly(*rac*-**3**) was determined by ^{13}C NMR spectrum.

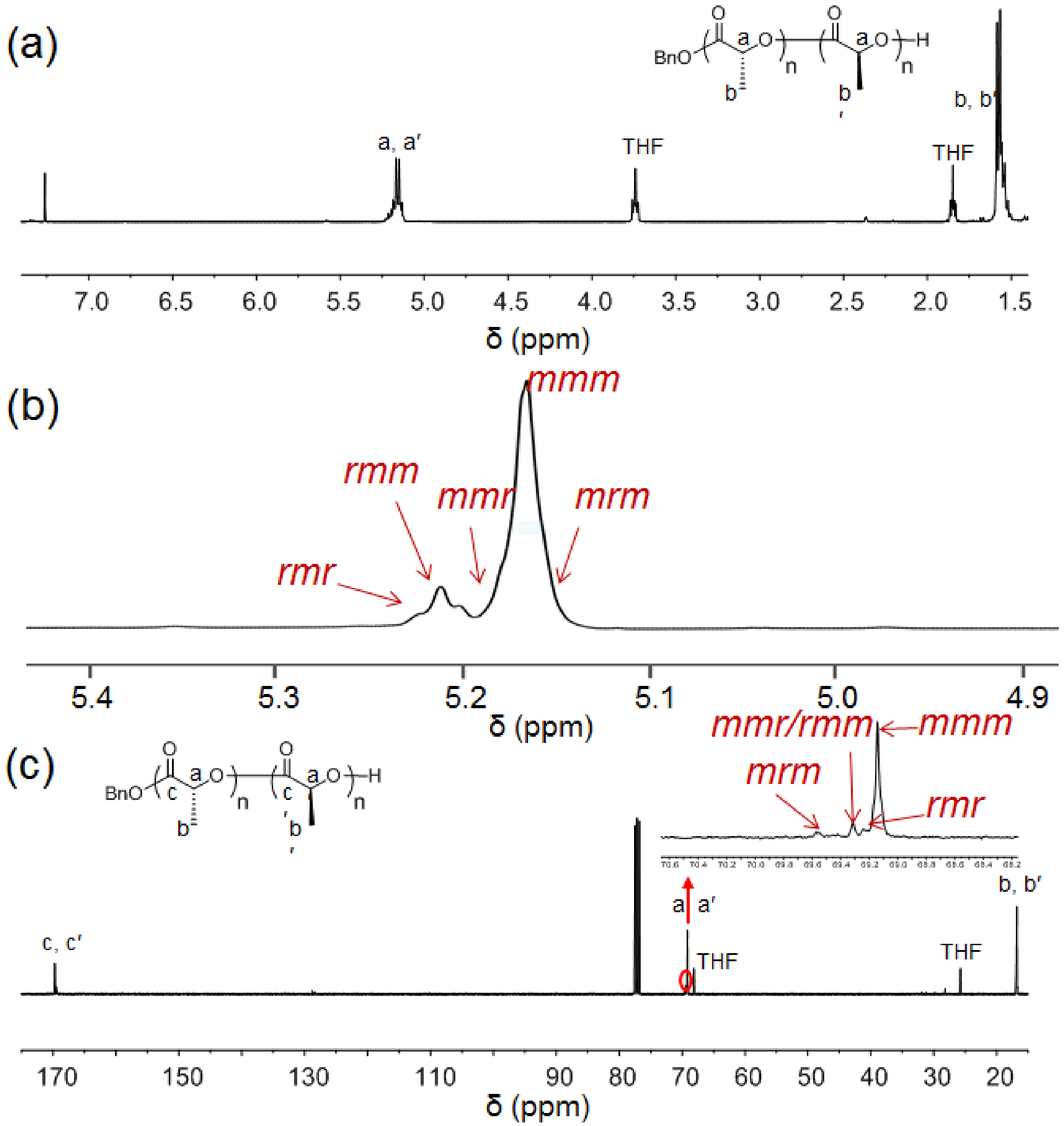

Supplementary Figure 9. NMR spectra of poly(*sb*-**4**) in $CDCl_3$ (Table 2, entry 5). (a) ^1H NMR spectrum; (b) Homodecoupling ^1H NMR spectrum; (c) ^{13}C NMR spectrum.

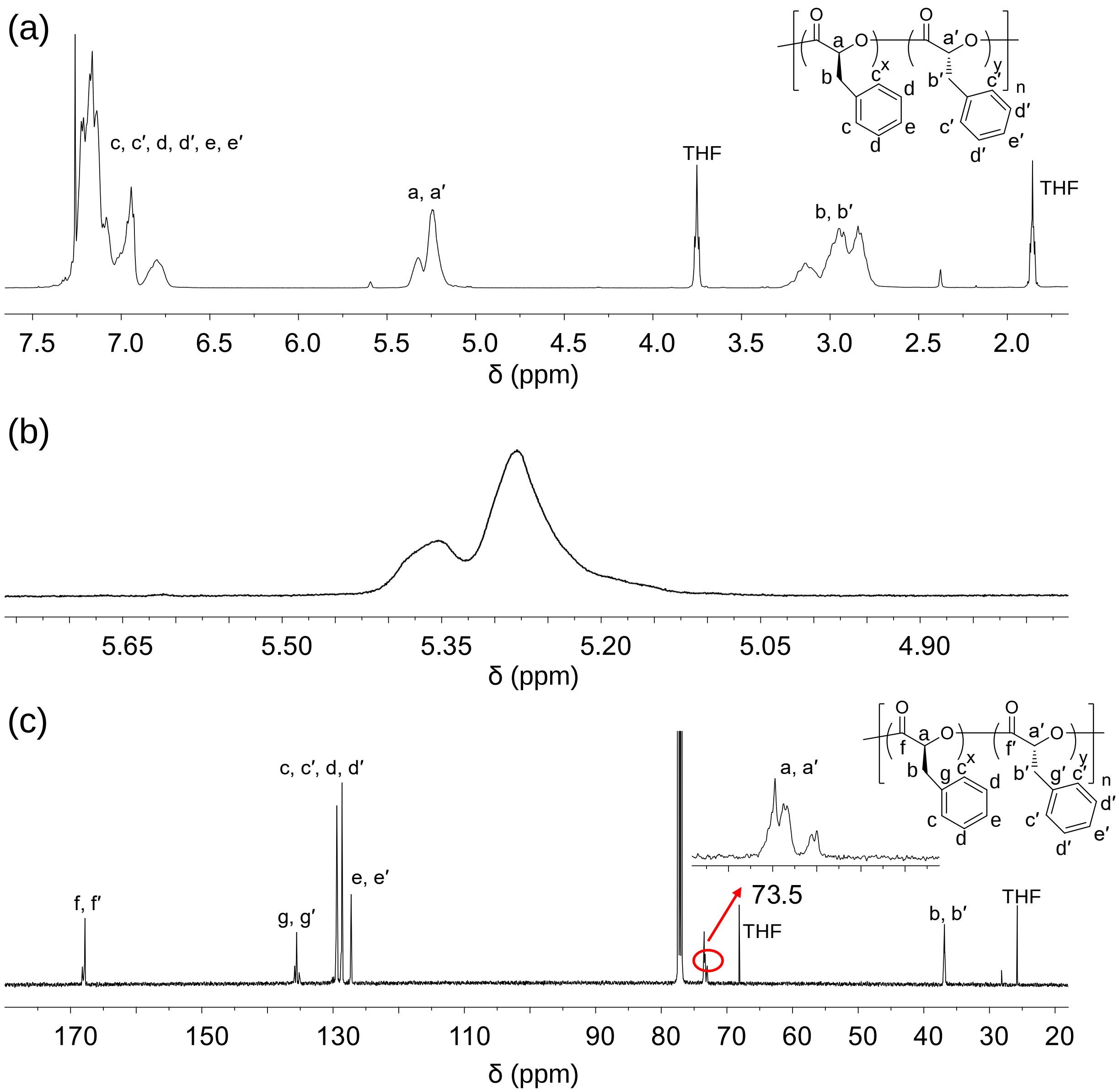

Supplementary Figure 10. NMR spectra of poly(L-**1**-*co*-D-**1**) in $CDCl_3$ (Table 2, entry 6; catalyzed by $Zn(HMDS)_2$). (a) ^1H NMR spectrum; (b) ^1H homodecoupling NMR spectrum; (c) ^{13}C NMR spectrum.

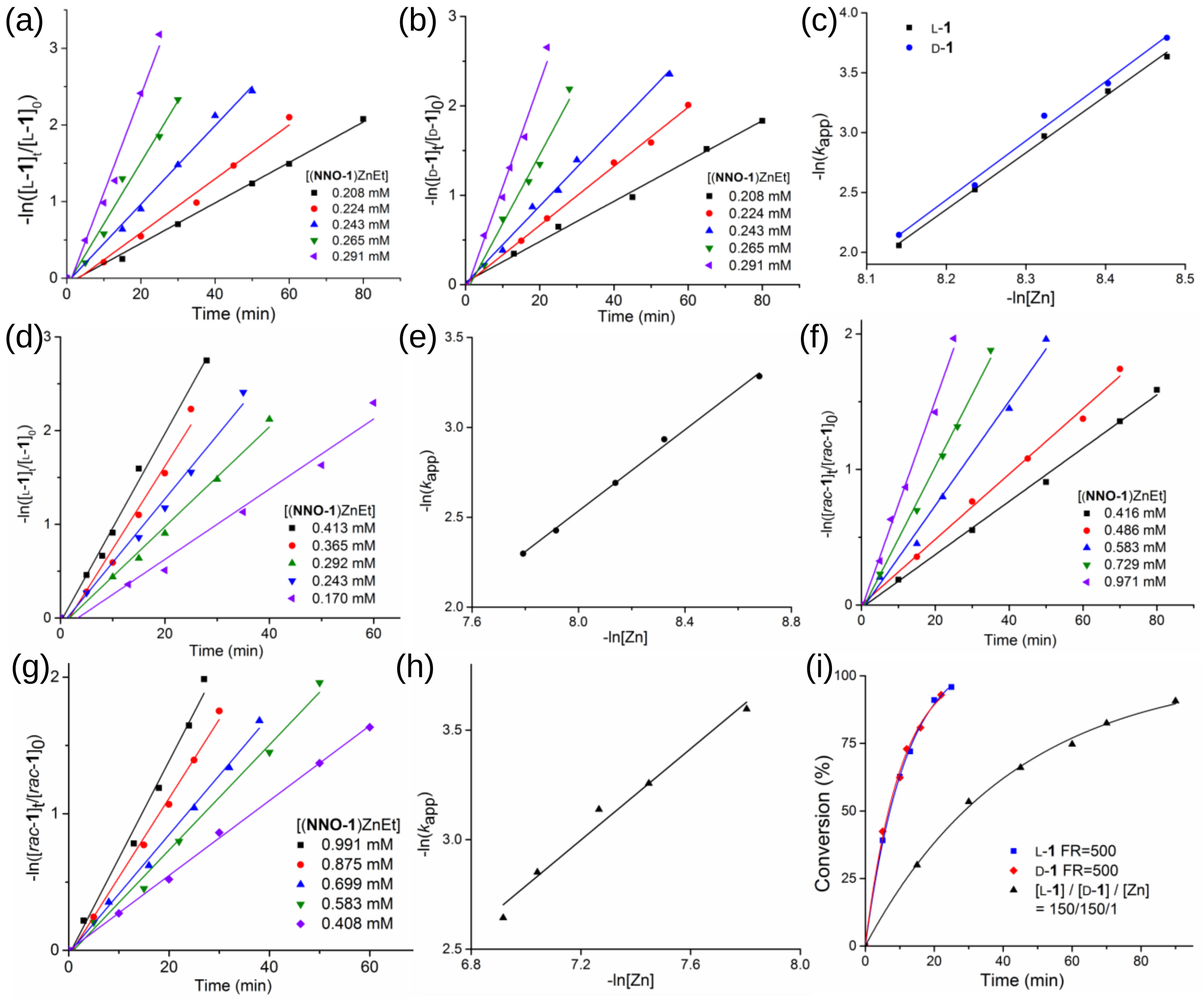

Supplementary Figure 11. Kinetic plots of the photoredox polymerization of (a) L-**1** or (b) D-**1** with variation of catalysts' concentrations at -15 °C. ([L-**1**] = 145.7 mM; [(bpy)Ni(COD)]/[(**NNO-1**)ZnEt]/[BnOH]/[**Ir-1**] =1/1/1/0.1). (c) Plot of -ln(k_{app}) versus –ln[Zn] for ROP of L-**1** and D-**1**. (d) Kinetic plots of the photoredox polymerization of L-**1** with only variation of [(**NNO-1**)ZnEt]. ([L-**1**] = 145.7 mM; [L-**1**]/[(bpy)Ni(COD)]/[BnOH]/[**Ir-1**] = 600/1/1/0.1). (e) Plot of -ln(k_{app}) versus –ln[Zn] for ROPs in (d). The slop, 1.13, is the reaction order of (**NNO-1**)ZnEt. (f) Kinetic plots of the photoredox polymerization of *rac*-**1** with variation of catalysts' concentrations at -15 °C. ([L-**1**] = [D-**1**] = 72.85 mM; [(bpy)Ni(COD)]/[(**NNO-1**)ZnEt]/[BnOH]/[**Ir-1**] = 1/1/1/0.1). (g) Kinetic plots of the photoredox polymerization of *rac*-**1** with only variation of [(**NNO-1**)ZnEt]. ([L-**1**] = [D-**1**] = 72.85 mM; [L-**1**]/[D-**1]**/[(bpy)Ni(COD)]/[BnOH]/[**Ir-1**] = 125/125/1/1/0.1). (h) Plot of -ln(k_{app}) versus –ln[Zn] for ROPs in (g). The slope, 1.04, is the reaction order of (**NNO-1**)ZnEt. (i) Plots of monomer conversion in polymerization reactions of L-**1** ([L-**1**]/[Zn] = 500), D-**1** ([D-**1**]/[Zn] = 500), and *rac*-**1** ([L-**1**]/[D-**1**]/[(bpy)Ni(COD)]/[(**NNO-1**)ZnEt]/[BnOH]/[**Ir-1**] = 150/150/1/1/1/0.1; [**1**] =

145.7 mM). The results suggest that the polymerization rate of *rac*-**1** is slower than that of either enantiomer.

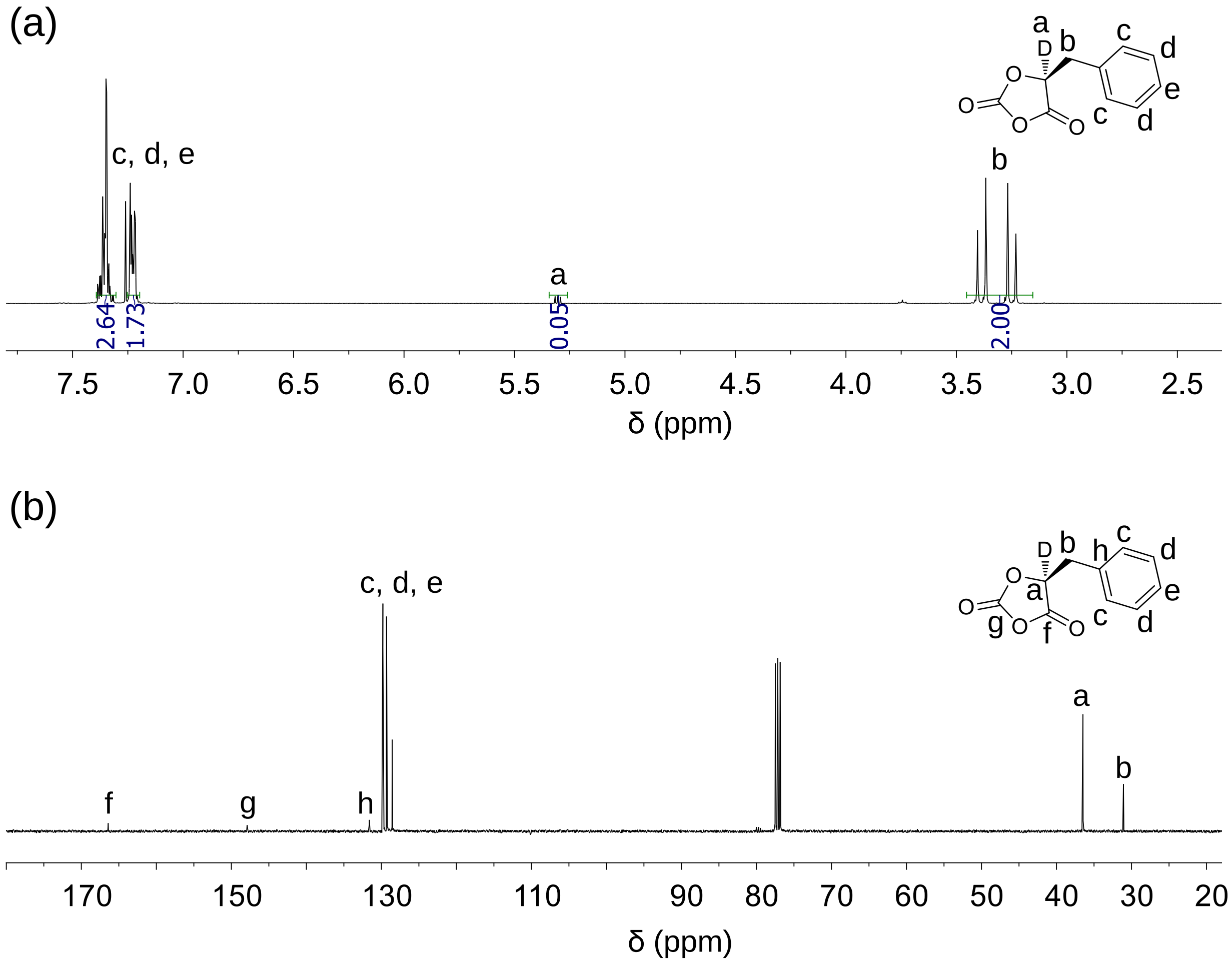

Supplementary Figure 12. ^{1}H and ^{13}C NMR spectra of deuterated monomer [D_2]-L-**1** in $CDCl_3$.

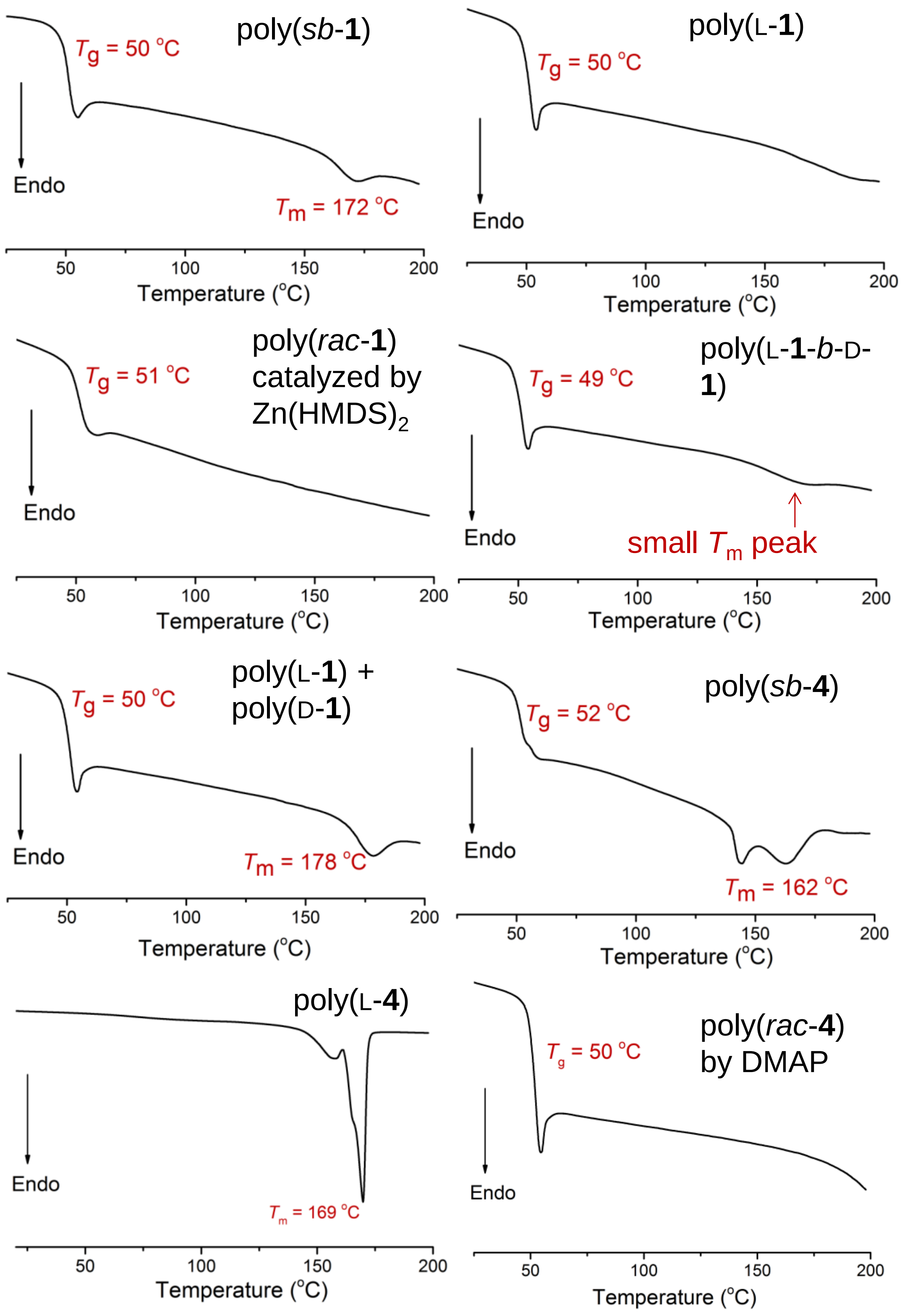

Supplementary Figure 13. DSC thermograms of polymers with various microstructures (see Supplementary Table 2).

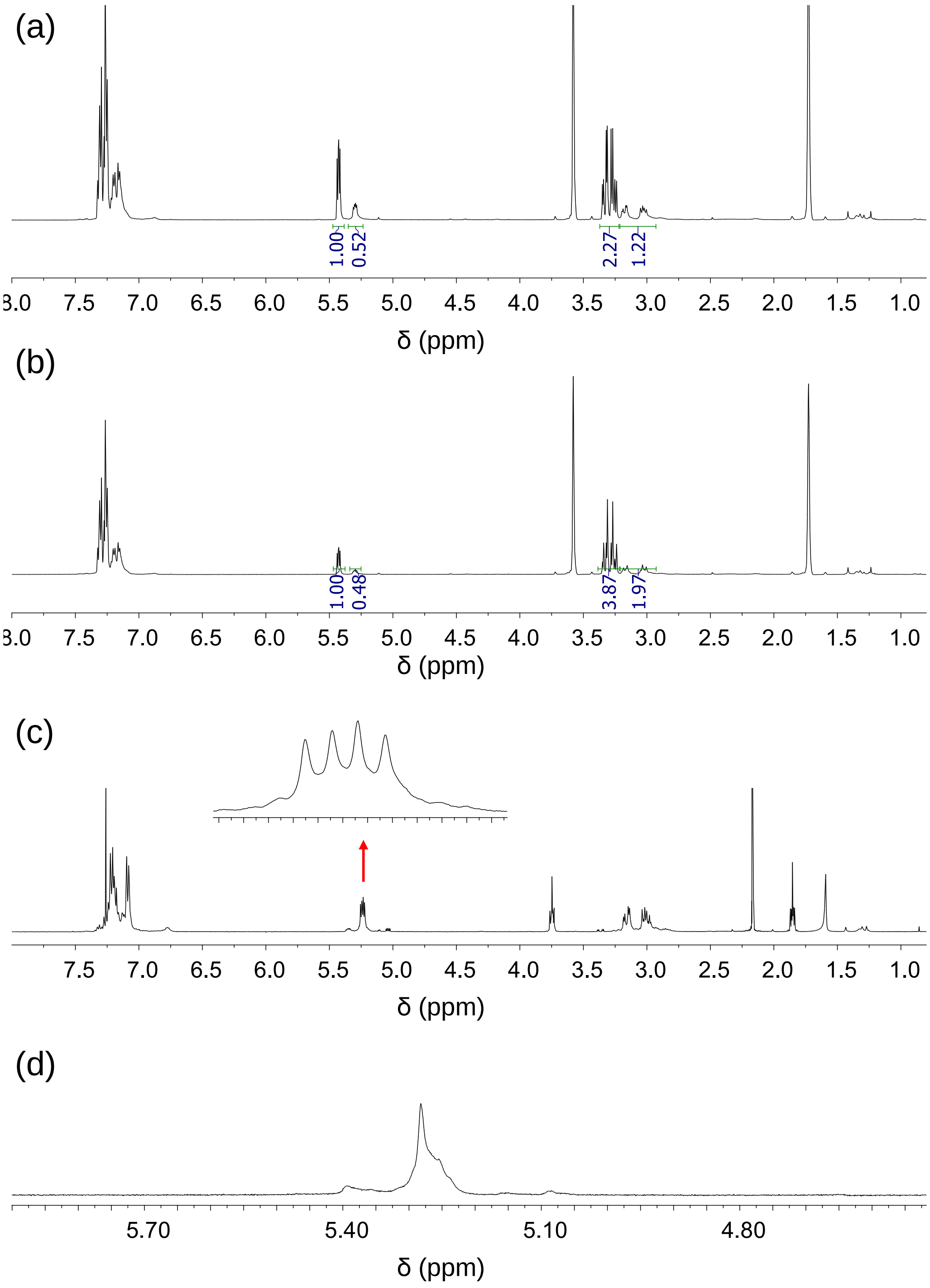

Supplementary Figure 14. ^{1}H NMR spectra of polymer prepared by the ROP condition at room temperature without Ni catalyst, **Ir-1** and light irradiation. (a) The polymerization of *rac*-**1** for 160 min in THF-d_8 ([L-**1**]/[D-**1**]/[(**NNO-1**)ZnEt]/[BnOH] = 50/50/1/1). (b) The polymerization of $[D_2]$-L-**1** and d-1 at the ratio of 50/50 for 160 min in THF-d_8 ([$[D_2]$-L-**1**]/[D-**1**]/[(**NNO-1**)ZnEt]/[BnOH] = 50/50/1/1). (c) The polymerization in (a) over 20 h with 100% conversion of *rac*-**1**. (d) homodecoupling ^{1}H NMR spectrum for poly(*rac*-**1**) in (c).

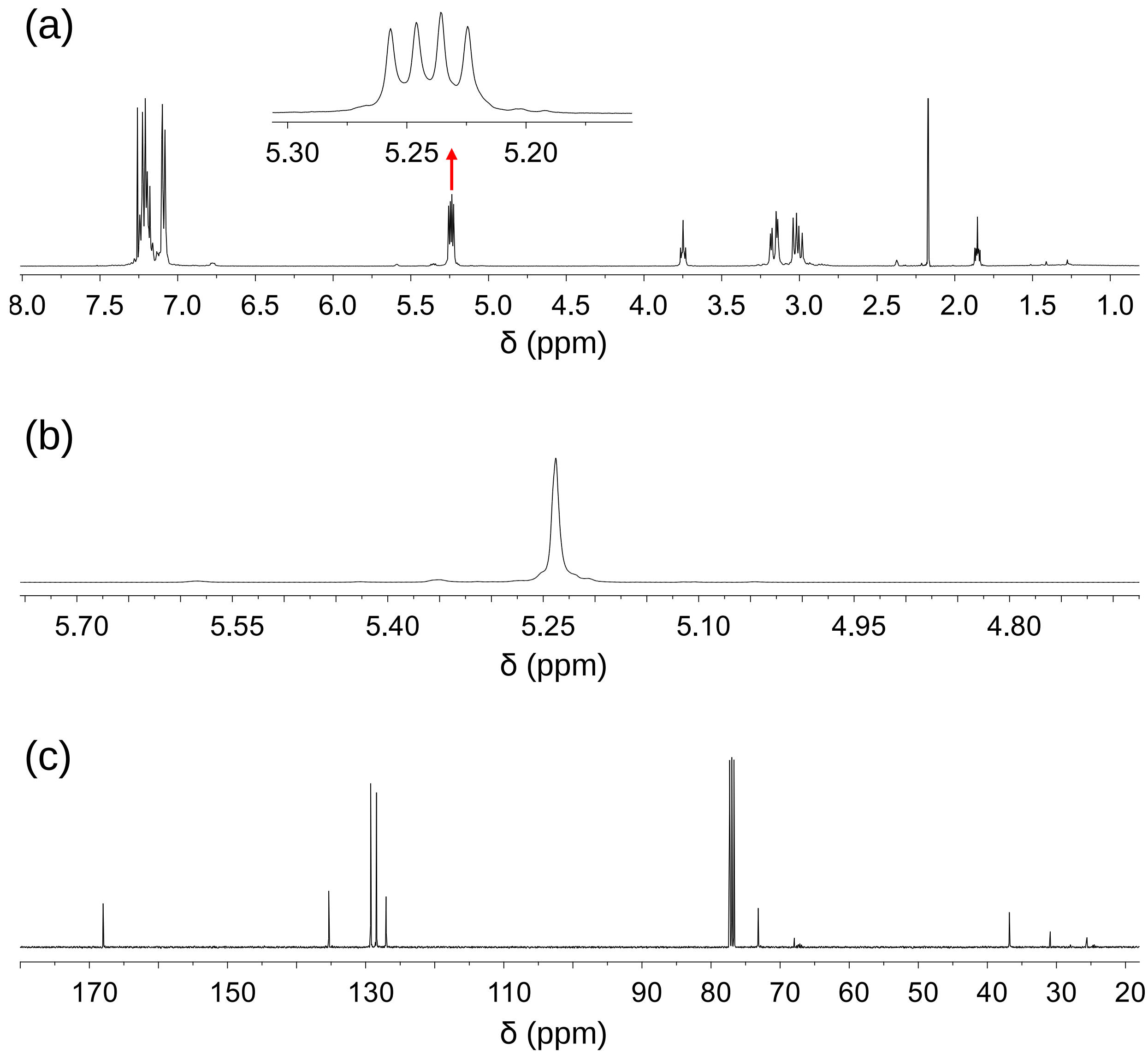

Supplementary Figure 15. NMR spectra of poly(*sb*-**1**) with a FR of 600 and 2 equivalent (bpy)Ni(COD) catalyst, prepared by the photoredox ROP ([L-**1**]/[D-**1**]/[(bpy)Ni(COD)]/[(**NNO-1**)ZnEt]/[BnOH]/[**Ir-1**] = 300/300/2/1/1/0.1; Table 2, entry 8). (a) ^{1}H NMR spectrum; (b) Homodecoupling ^{1}H NMR spectrum; (c) ^{13}C NMR spectrum (all spectra in $CDCl_3$).

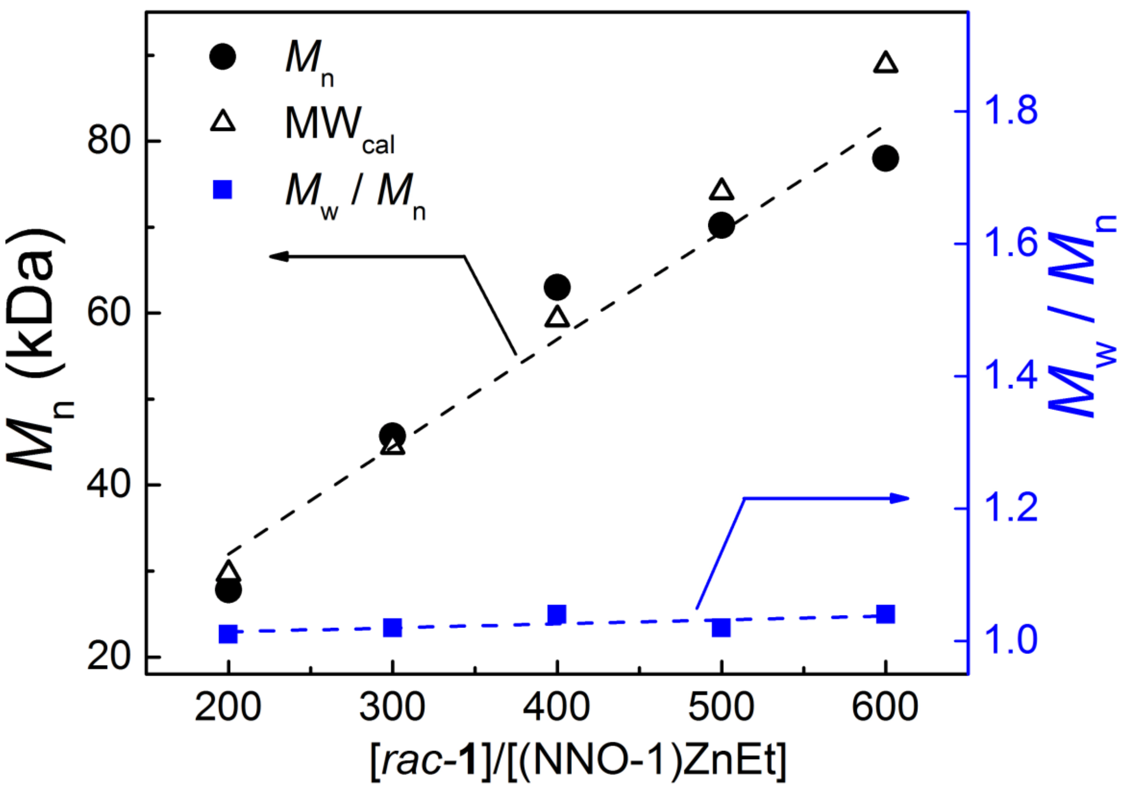

Supplementary Figure 16. Plots of M_n and molecular weight distribution (M_w/M_n) of poly(*sb*-**1**) versus [*rac*-**1**]/[(**NNO-1**)ZnEt] ratio ([L-**1**]/[D-**1**]=1/1, [(**NNO-1**)ZnEt] / [(bpy)Ni(COD)] /[BnOH] /[**Ir-1**] = 1/1/1/0.1).

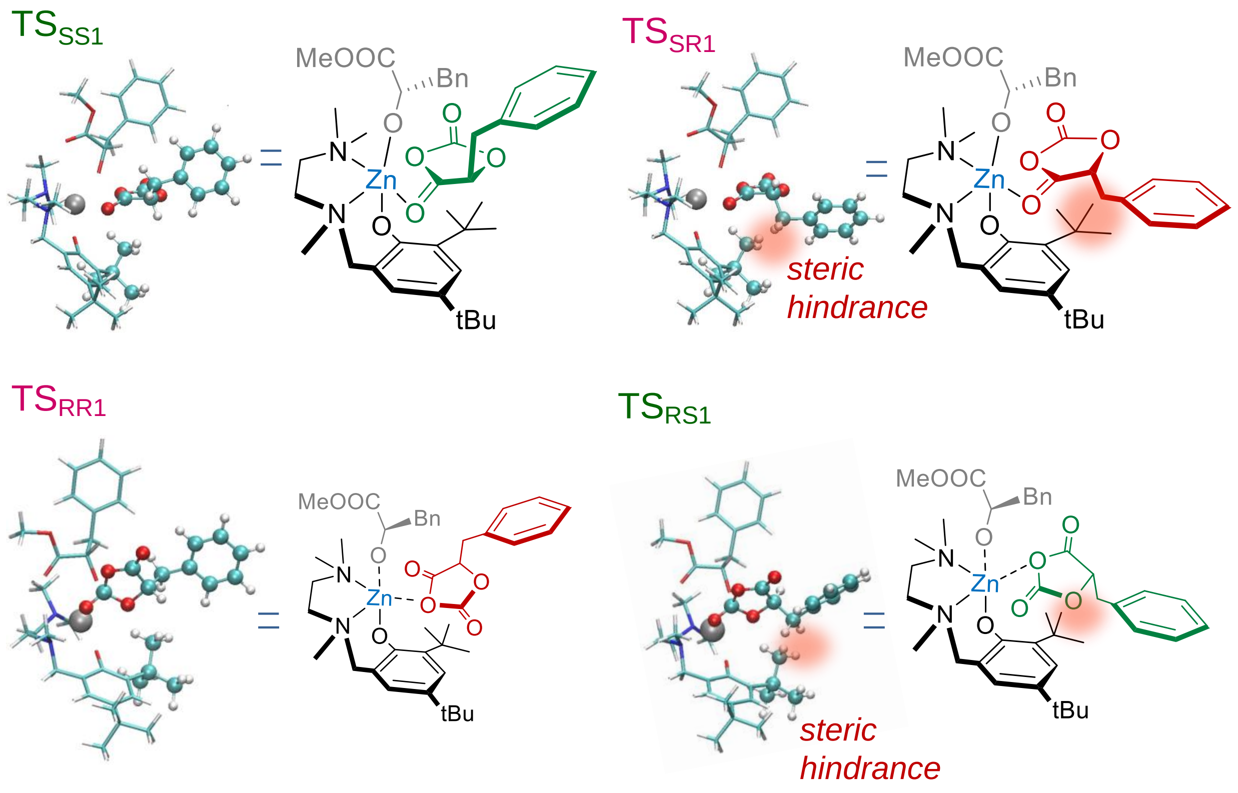

Supplementary Figure 17. Stereochemical models for transition states TS$_{SS1}$, TS$_{SR1}$, TS$_{SS1}$, and TS$_{RS1}$ for ring-opening reactions of **1** mediated by (NNO-1)Zn/**5**. Note that the chirality of **5** could affect the orientation of **1** that approaches the Zn center, by the spatial position of the benzyl group in **5**. The steric interactions in TS$_{SR1}$ and TS$_{RS1}$ result in less favored and high-energy state in the chain propagation.

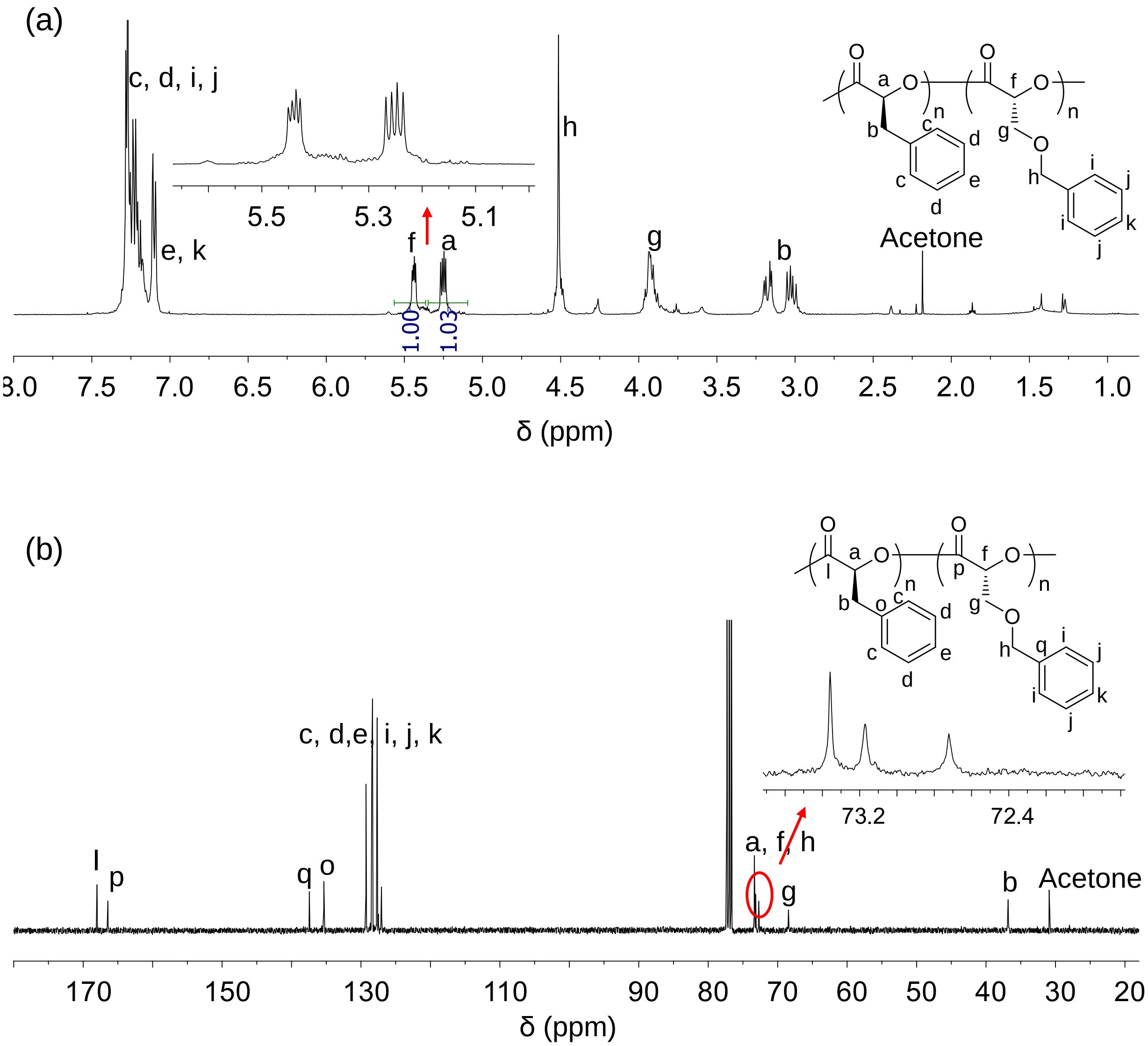

Supplementary Figure 18. NMR spectra of poly(L-**1**-*grad*-D-**2**) in $CDCl_3$ ([L-**1**]/[D-**2**]/[(bpy)Ni(COD)]/[(**NNO-1**)ZnEt]/[BnOH]/[**Ir-1**] = 100/100/1/1/1/0.1; Table 3, entry 1). (a) ^{1}H NMR spectrum; (b) ^{13}C NMR spectrum.

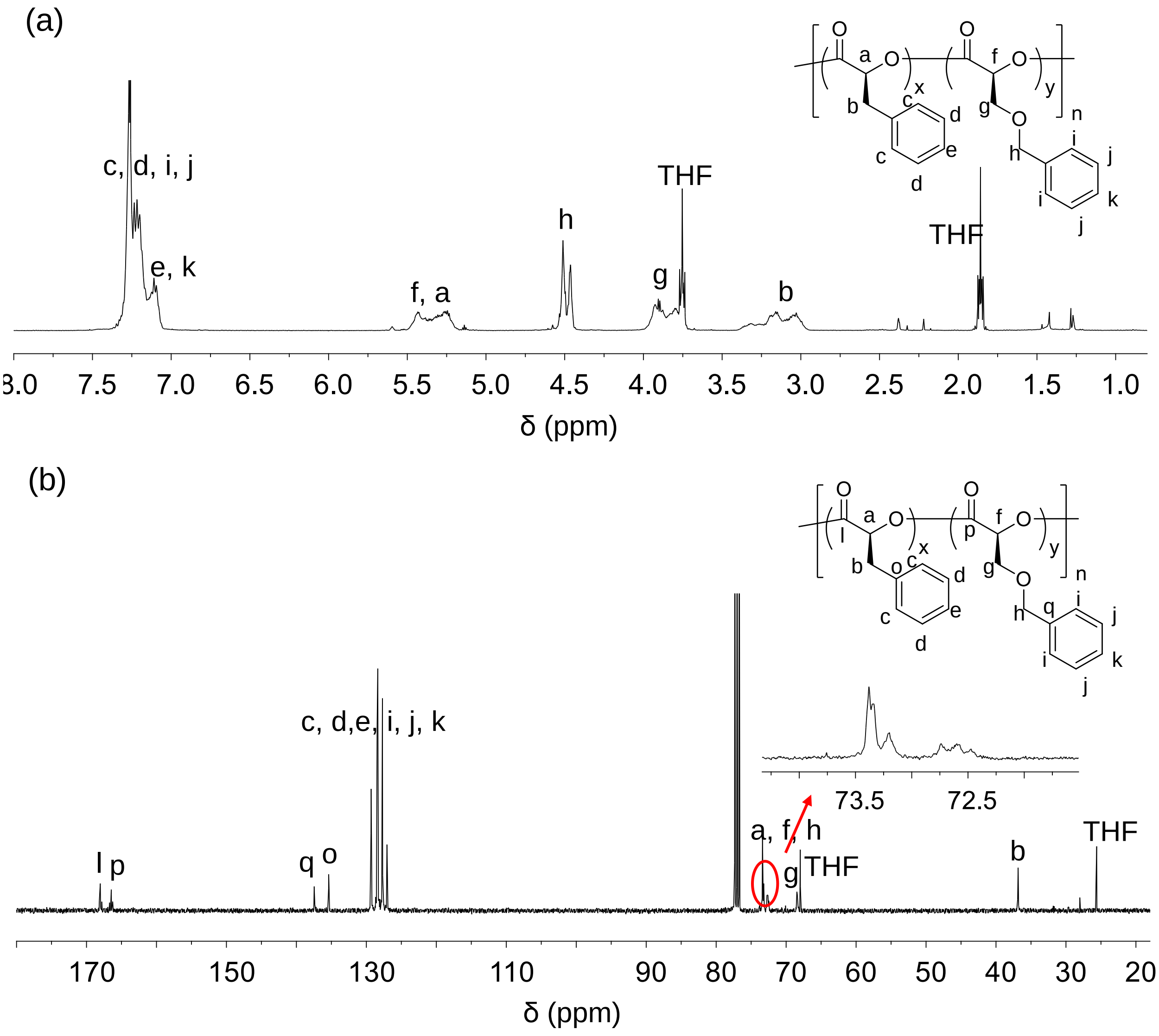

Supplementary Figure 19. NMR spectra of poly(L-**1**-*co*-L-**2**) in $CDCl_3$ ([L-**1**]/[L-**2**]/[(bpy)Ni(COD)]/[(**NNO-1**)ZnEt]/[BnOH]/[**Ir-1**] = 100/100/1/1/1/0.1; Table 3, entry 2). (a) ^{1}H NMR spectrum; (b) ^{13}C NMR spectrum. Note that comparing with ^{1}H NMR spectrum of poly(L-**1**-*grad*-D-**2**) (Supplementary Figure 16), peaks in (a) were broader without distinct splitting. In addition, multiple peaks have been observed in the α-methine region (~ 73 ppm, peaks *a* and *f*) in ^{13}C NMR spectrum, suggesting the random sequence in poly(L-**1**-*co*-L-**2**).

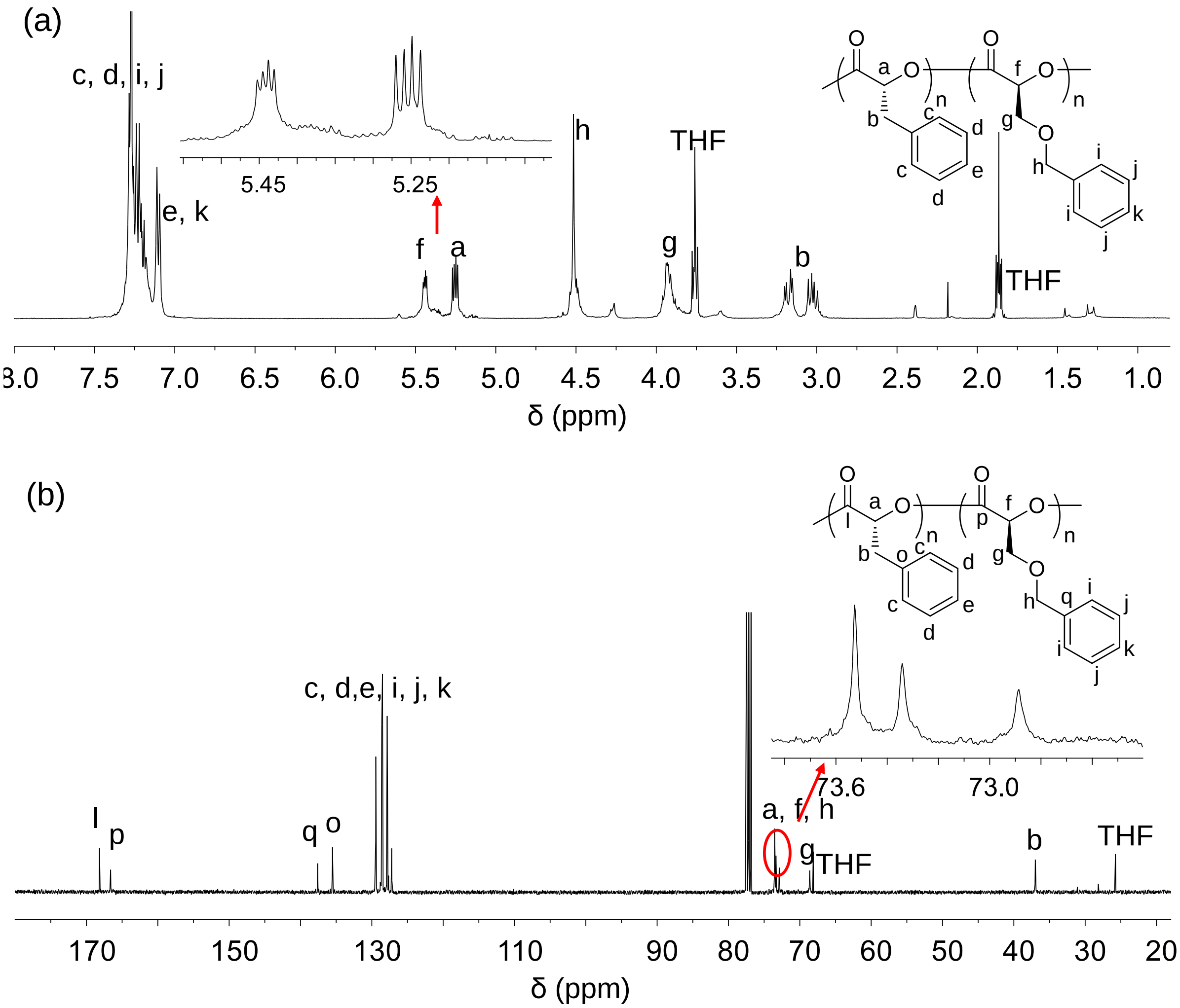

Supplementary Figure 20. NMR spectra of poly(D-**1**-*grad*-L-**2**) in $CDCl_3$ ([D-**1**]/[L-**2**]/[(bpy)Ni(COD)]/[(**NNO-1**)ZnEt]/[BnOH]/[**Ir-1**] = 100/100/1/1/1/0.1; Table 3, entry 3). (a) ^{1}H NMR spectrum; (b) ^{13}C NMR spectrum.

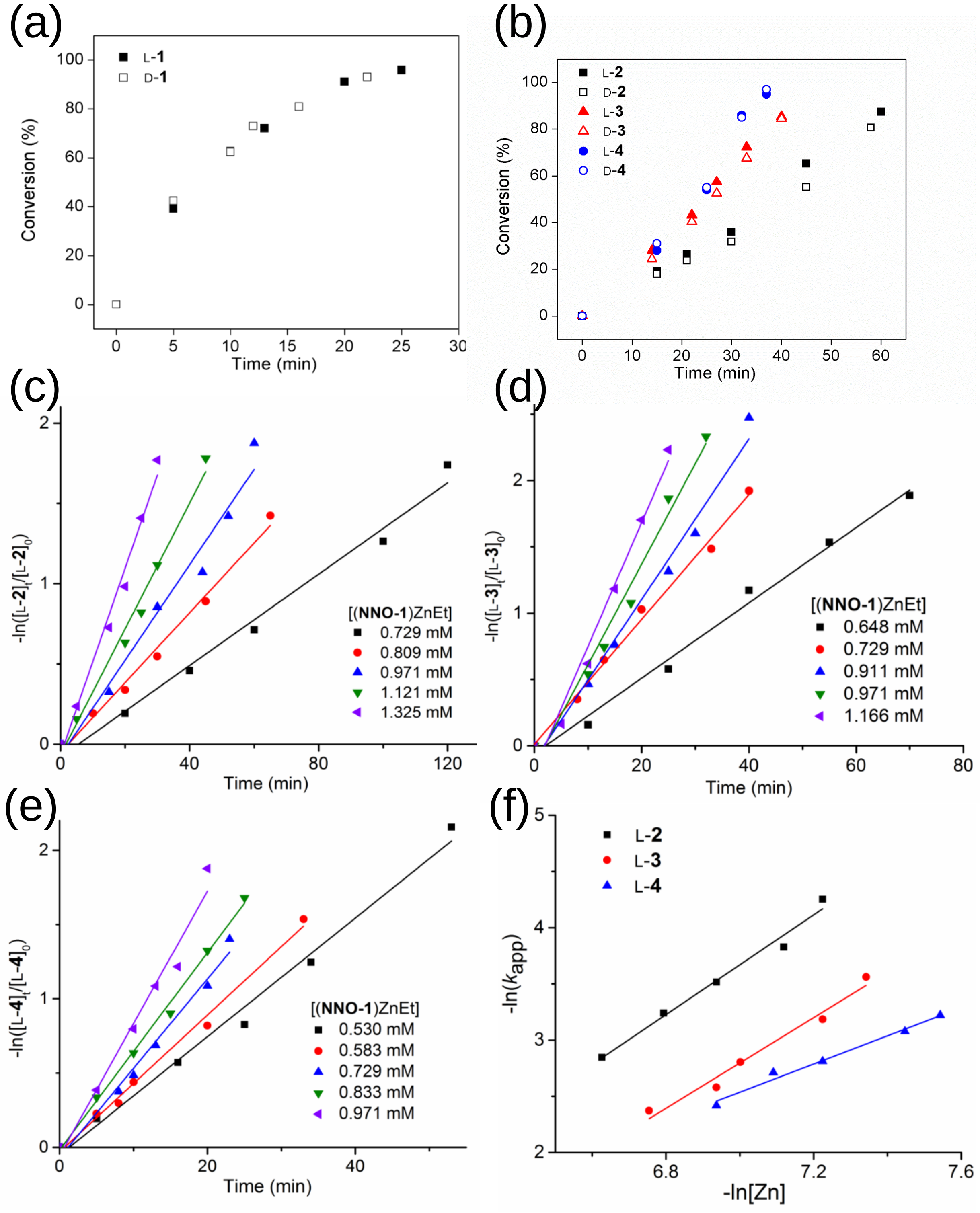

Supplementary Figure 21. Plot of OCA monomer conversion versus reaction time ([OCA] = 145.7 mM in all studies; [(bpy)Ni(COD)]/[(**NNO-1**)ZnEt]/[BnOH]/[**Ir-1**] =1/1/1/0.1). (a) FR = 500 for **1**; (b) FR = 200 for **2, 3, 4**. The use of high FR (500) for 1 is due to the rapid kinetic rates

of **1** (at FR=200, the photoredox polymerization of **1** finishes within 5 minutes and it was difficult to acquire enough data points). (c-e) Kinetic plots of the photoredox polymerization of (c) L-**2,** (d) L-**3** or (e) L-**4** with variation of catalysts' concentrations at -15 ^{o}C. ([L-OCA] = 145.7 mM; [(bpy)Ni(COD)]/[(**NNO-1**)ZnEt]/[BnOH]/[**Ir-1**] =1/1/1/0.1). (f) Plot of -ln(k_{app}) versus –ln[Zn] for ROPs in (c-e). Based on the kinetics plots, including that in Supplementary Figure 11c of **1** (note the increased FRs of **1** in Supplementary Figure 11c for similar kinetic rates), OCA monomers' polymerization rates decrease in the order k(**1**) > k(**3**) ≈ k(**4**) > k(**2**).

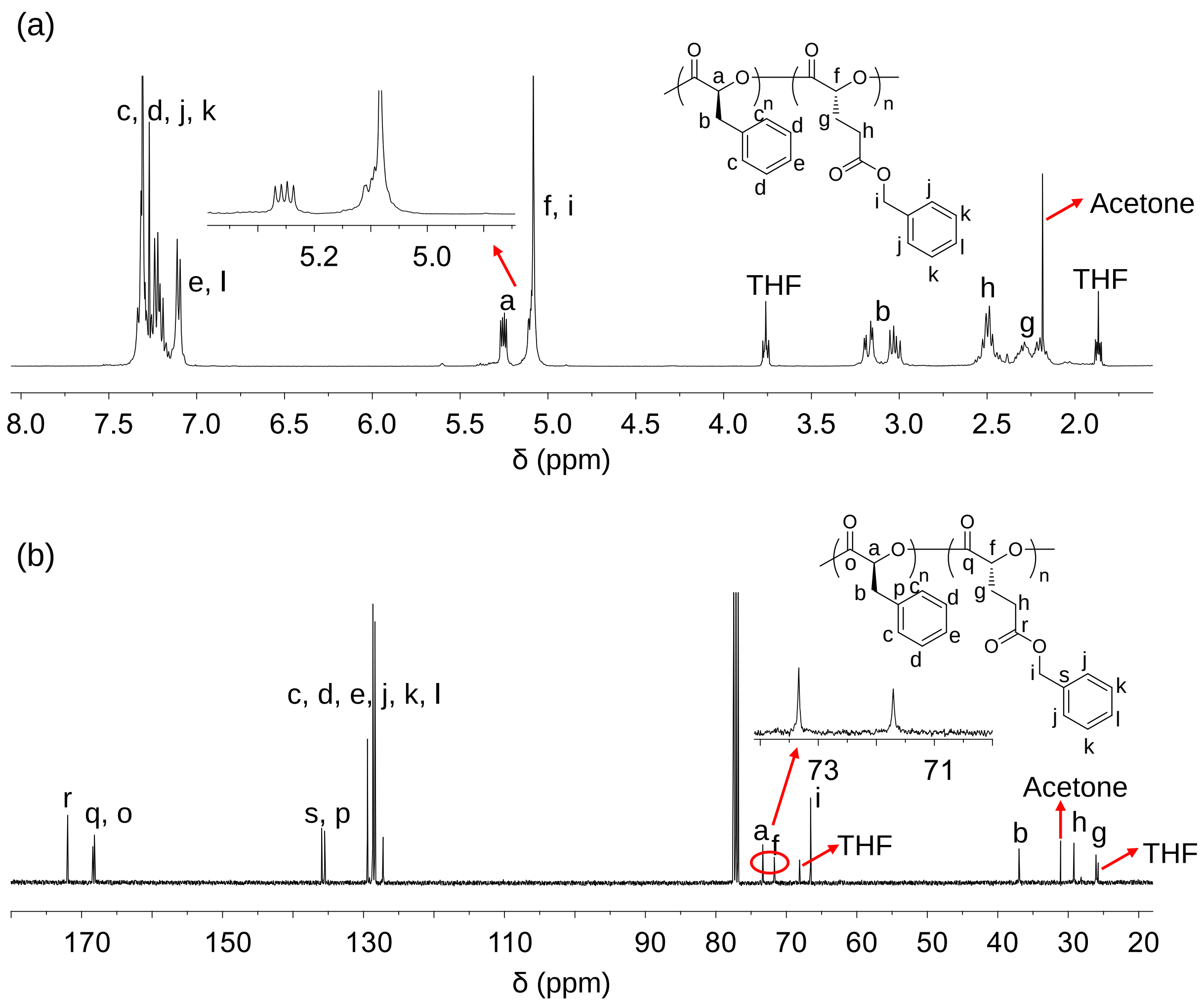

Supplementary Figure 22. NMR spectra of poly(L-**1**-*grad*-D-**3**) in $CDCl_3$ ([L-**1**]/[D-**3**]/[(bpy)Ni(COD)]/[(**NNO-1**)ZnEt]/[BnOH]/[**Ir-1**] = 100/100/1/1/1/0.1; Table 3, entry 4). (a) ^{1}H NMR spectrum; (b) ^{13}C NMR spectrum.

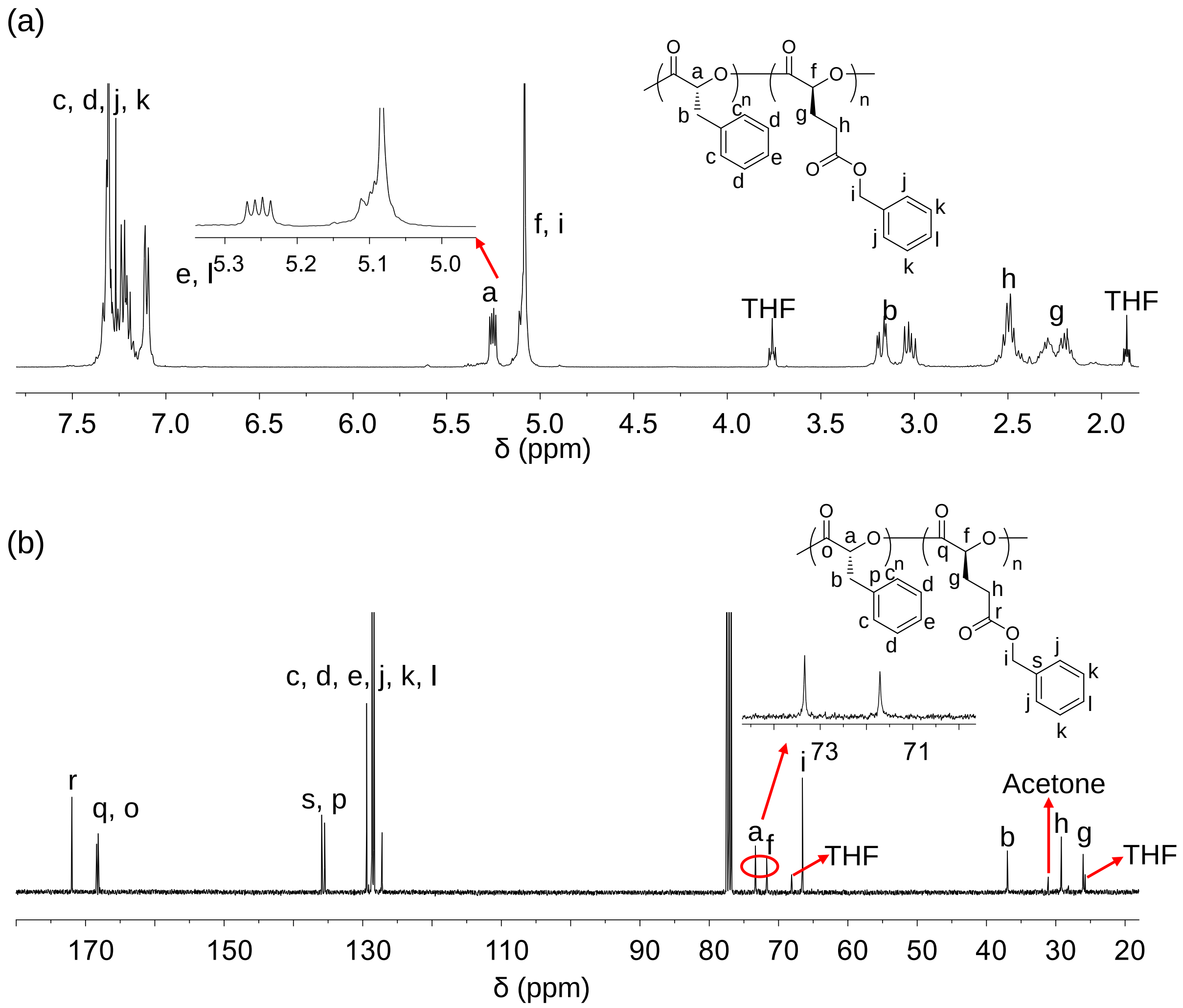

Supplementary Figure 23. NMR spectra of poly(D-**1**-*grad*-L-**3**) in $CDCl_3$ ([D-**1**]/[L-**3**]/[(bpy)Ni(COD)]/[(**NNO-1**)ZnEt]/[BnOH]/[**Ir-1**] = 100/100/1/1/1/0.1; Table 3, entry 5). (a) ^{1}H NMR spectrum; (b) ^{13}C NMR spectrum.

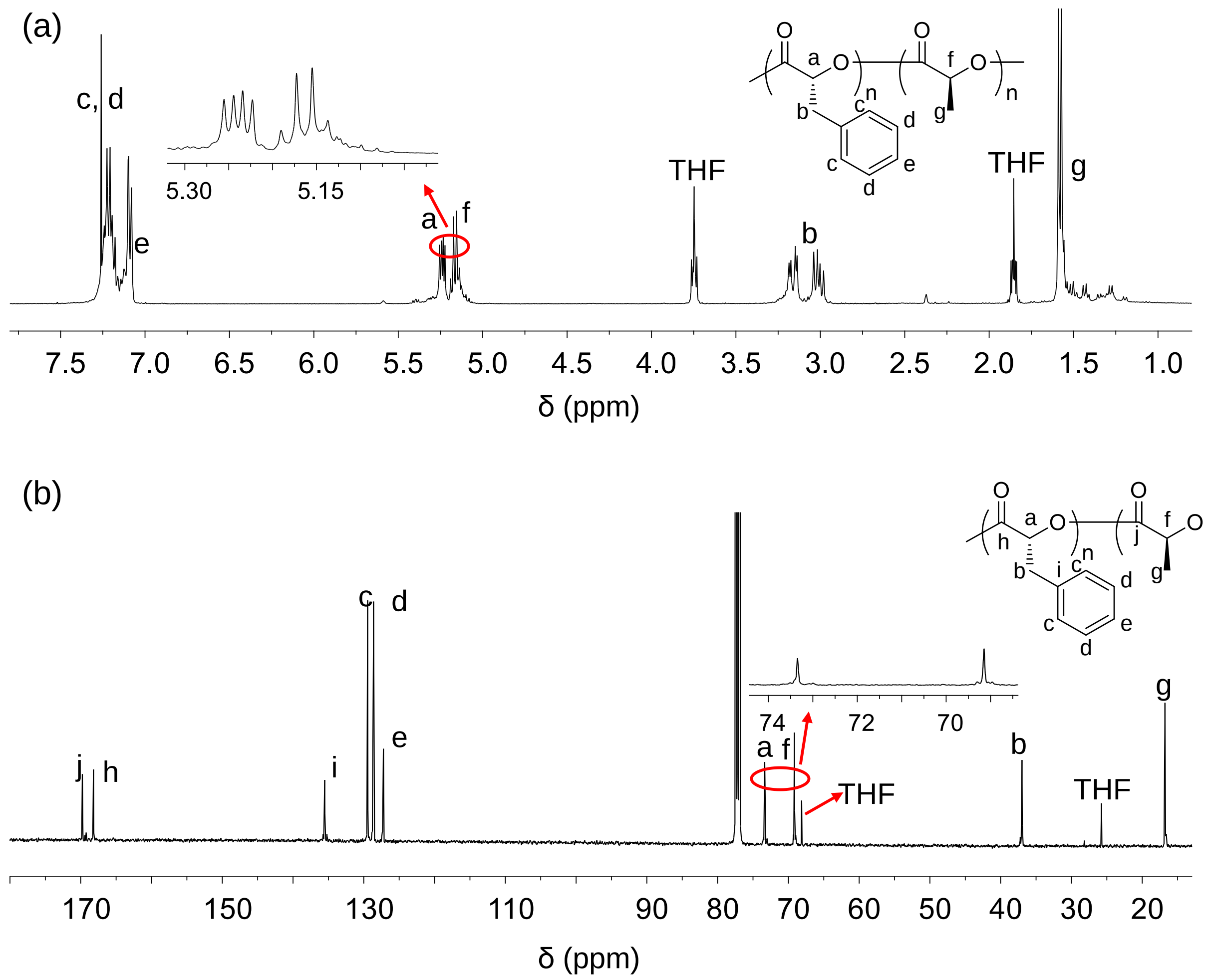

Supplementary Figure 24. NMR spectra of poly(L-**1**-*grad*-D-**4**) in $CDCl_3$ ([L-**1**]/[D-**4**]/[(bpy)Ni(COD)]/[(**NNO-1**)ZnEt]/[BnOH]/[**Ir-1**] = 200/200/1/1/1/0.1; Table 3, entry 6). (a) ^{1}H NMR spectrum; (b) ^{13}C NMR spectrum.

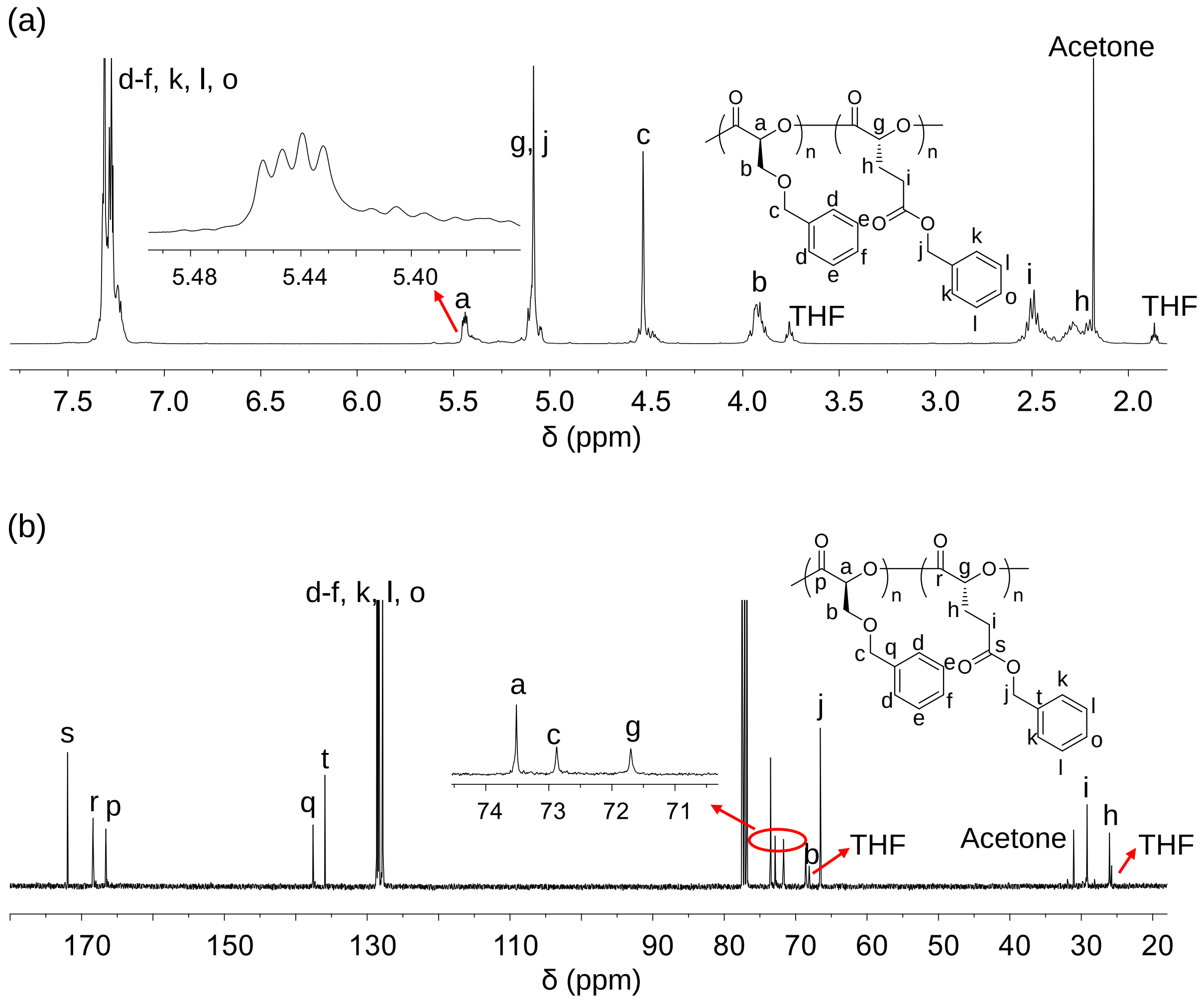

Supplementary Figure 25. NMR spectra of poly(L-**2**-*grad*-D-**3**) in $CDCl_3$ ([L-**2**]/[D-**3**]/[(bpy)Ni(COD)]/[(**NNO-1**)ZnEt]/[BnOH]/[**Ir-1**] = 100/100/1/1/1/0.1; Table 3, entry 7). (a) ^1H NMR spectrum; (b) ^{13}C NMR spectrum.

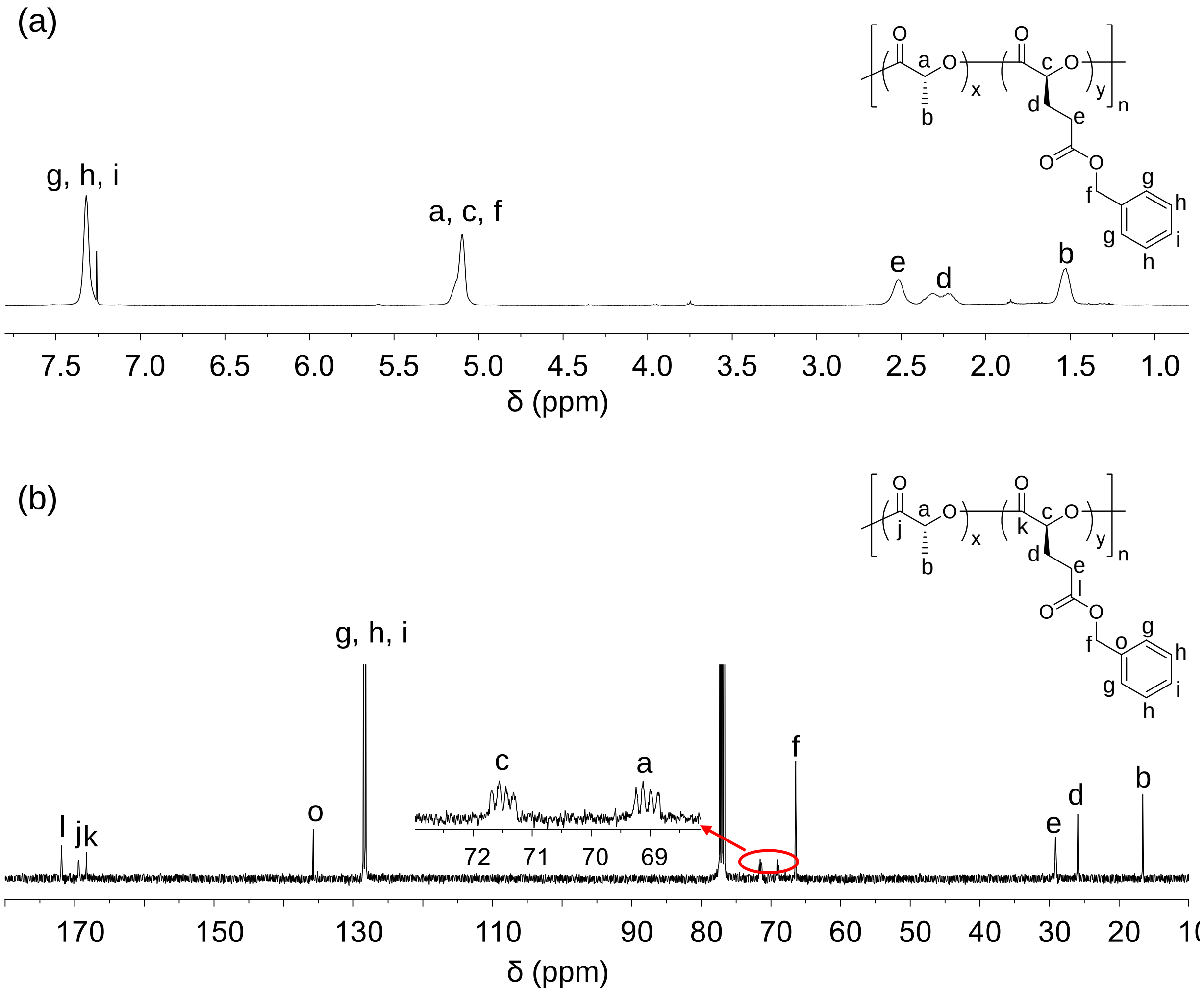

Supplementary Figure 26. NMR spectra of poly(L-**3**-*co*-D-**4**) in $CDCl_3$ ([L-**3**]/[D-**4**]/[(bpy)Ni(COD)]/[(**NNO-1**)ZnEt]/[BnOH]/[**Ir-1**] = 200/200/1/1/1/0.1; Table 3, entry 8). (a) ^{1}H NMR spectrum; (b) ^{13}C NMR spectrum. Note that multiple peaks with similar intensities have been observed in the α-methine region (~ 70 ppm, peaks *a* and *c*) in ^{13}C NMR spectrum, suggesting the random sequence in poly(L-**3**-*co*-D-**4**) (e.g., **3**-**3**-**3**; **3**-**3**-**4**; **4**-**3**-**3**; **4**-**3**-**4** for peaks *c* in L-**3**).

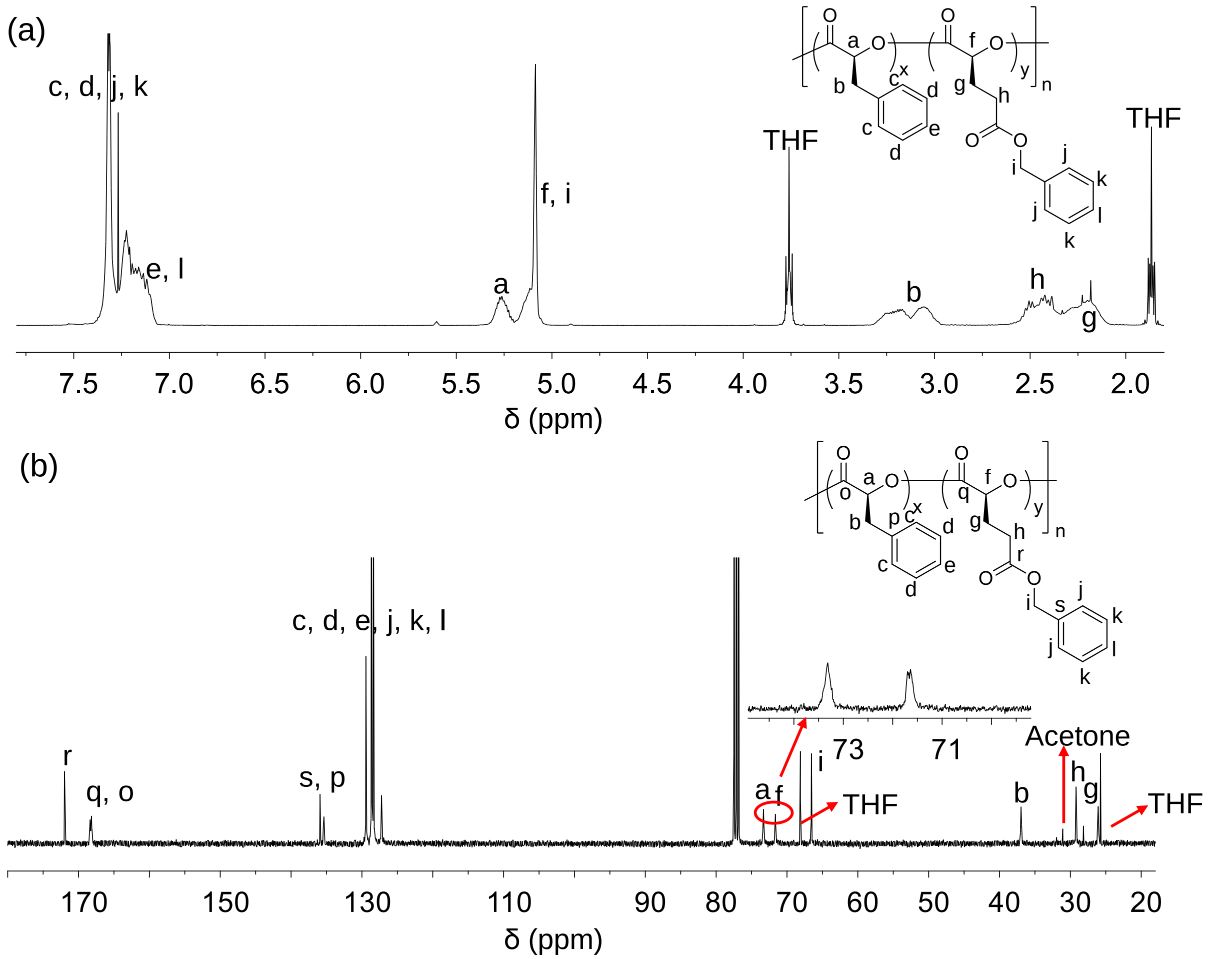

Supplementary Figure 27. NMR spectra of poly(L-**1**-*co*-L-**3**) in $CDCl_3$ ([L-**1**]/[L-**3**]/[(bpy)Ni(COD)]/[(**NNO-1**)ZnEt]/[BnOH]/[**Ir-1**] = 100/100/1/1/1/0.1; Table 3, entry 9). (a) ^1H NMR spectrum; (b) ^{13}C NMR spectrum. Note that comparing with ^1H NMR spectrum of poly(L-**1**-*grad*-D-**3**) (Figure S20), peaks in (a) were broader without distinct splitting, suggesting the random sequence in poly(L-**1**-*co*-L-**3**).

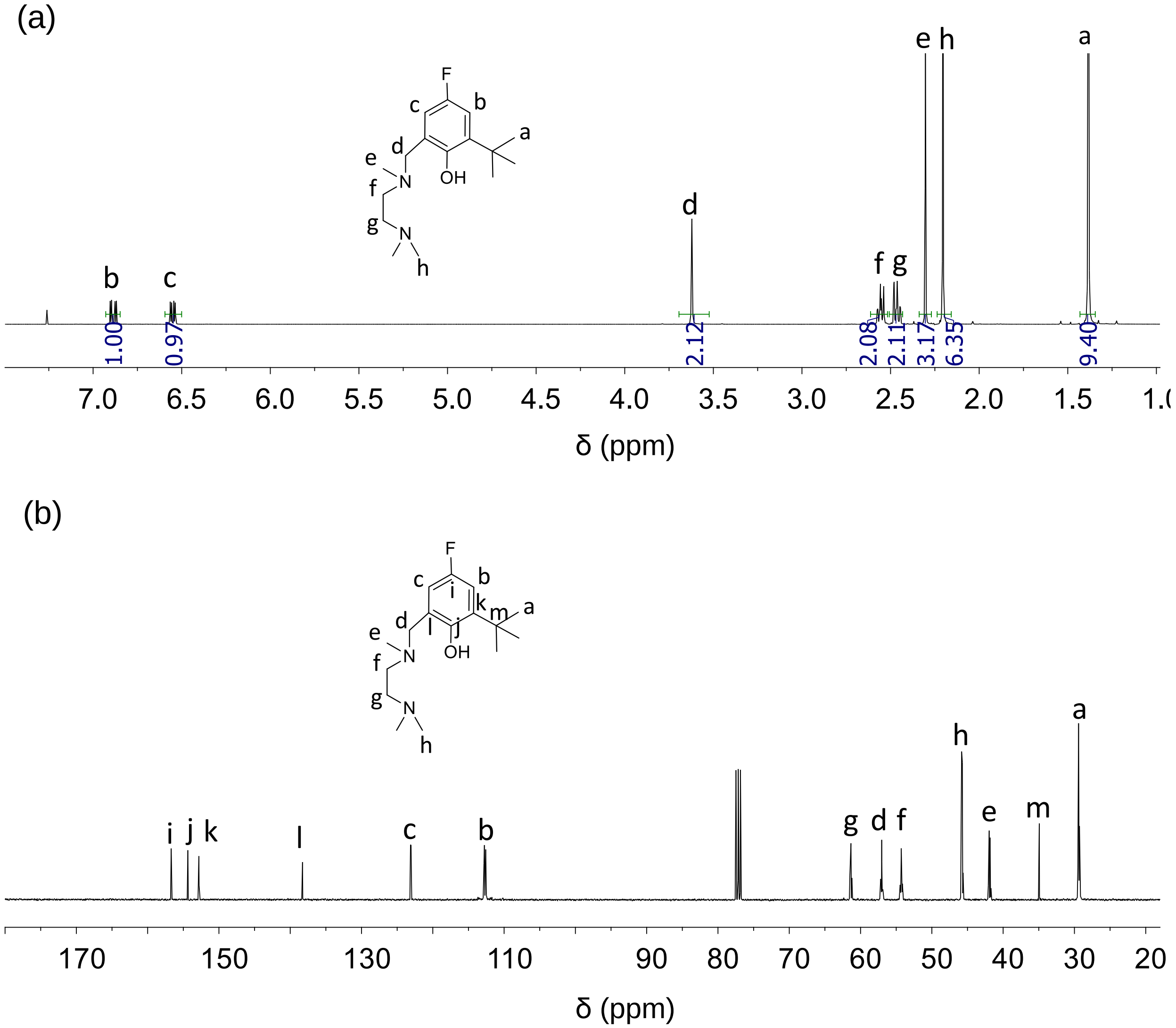

Supplementary Figure 28. NMR spectra of **NNO-4** ligand in $CDCl_3$. (a) ^{1}H NMR spectrum; (b) ^{13}C NMR spectrum.

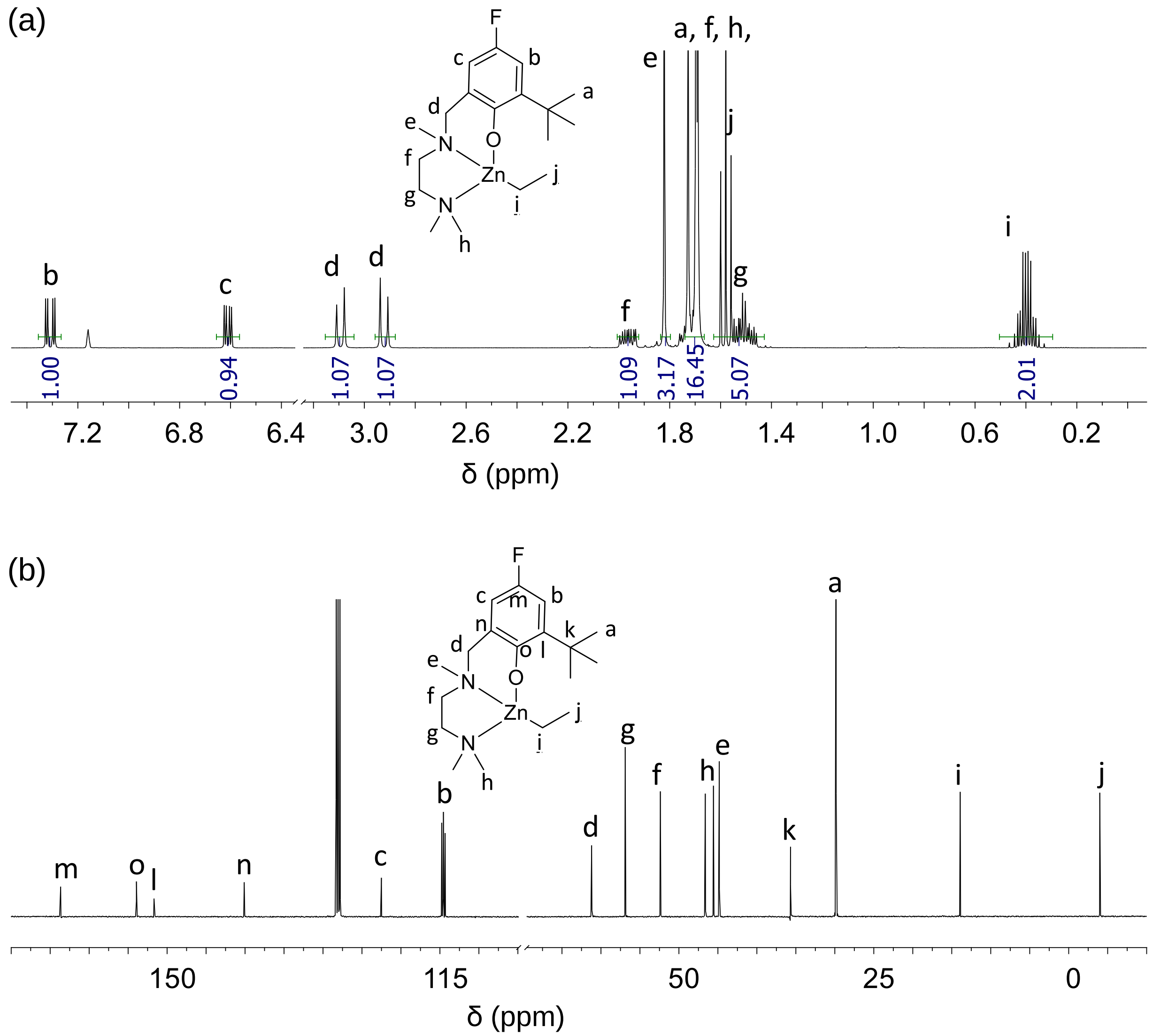

Supplementary Figure 29. NMR spectra of (**NNO-4**)ZnEt in C_6D_6. (a) ^{1}H NMR spectrum; (b) ^{13}C NMR spectrum.

Computational Methods

All quantum chemical calculations were carried out with the Gaussian 09 program. The geometries of all stationary points including reactants, transition states, products, and intermediates are optimized by Becke's three-parameter nonlocal-exchange gradient-corrected functional[11] with the nonlocal correlational of Lee, Yang, and Parr (B3LYP) method[12] with 3-21G basis sets for C, H, O, N atoms, and Los Alamos effective core potential basis set (LANL2DZ) for Zn atom. The harmonic vibrational frequency analyses were performed on all structures, including intermediate (all real frequencies) and transition states (one imaginary frequency) at the same level of theory. The minimum energy path (MEP) is calculated by intrinsic reaction coordinate (IRC) theory to confirm that transition states connect to the designated local minima. Thermodynamic corrections were also applied to determine the energies of the species at a temperature of 298.15 K.

Supplementary References

1. Yin Q, *et al.* Drug-Initiated Ring-Opening Polymerization of *O*-Carboxyanhydrides for the Preparation of Anticancer Drug–Poly (*O*-carboxyanhydride) Nanoconjugates. *Biomacromolecules* **14**, 920-929 (2013).
2. Lu Y, *et al.* Synthesis of Water-Soluble Poly(α-hydroxy acids) from Living Ring-Opening Polymerization of O-Benzyl-l-serine Carboxyanhydrides. *ACS Macro. Lett.* **1**, 441-444 (2012).
3. Thillaye du Boullay O, Bonduelle C, Martin-Vaca B, Bourissou D. Functionalized polyesters from organocatalyzed ROP of gluOCA, the *O*-carboxyanhydride derived from glutamic acid. *Chem. Commun.*, 1786-1788 (2008).
4. Thillaye du Boullay O, Marchal E, Martin-Vaca B, Cossío FP, Bourissou D. An activated equivalent of lactide toward organocatalytic ring-opening polymerization. *J. Am. Chem. Soc.* **128**, 16442-16443 (2006).
5. Williams CK, *et al.* A Highly Active Zinc Catalyst for the Controlled Polymerization of Lactide. *J. Am. Chem. Soc.* **125**, 11350-11359 (2003).
6. Brown NJ, *et al.* Mononuclear Phenolate Diamine Zinc Hydride Complexes and Their Reactions With CO_2. *Organometallics* **33**, 1112-1119 (2014).
7. Lee D-Y, Hartwig JF. Zinc Trimethylsilylamide as a Mild Ammonia Equivalent and Base for the Amination of Aryl Halides and Triflates. *Org. Lett.* **7**, 1169-1172 (2005).
8. DiCiccio AM, Longo JM, Rodríguez-Calero GG, Coates GW. Development of Highly Active and Regioselective Catalysts for the Copolymerization of Epoxides with Cyclic Anhydrides: An Unanticipated Effect of Electronic Variation. *J. Am. Chem. Soc.* **138**, 7107-7113 (2016).
9. Feng Q, Tong R. Controlled Photoredox Ring-Opening Polymerization of *O*-Carboxyanhydrides. *J. Am. Chem. Soc.* **139**, 6177-6182 (2017).
10. Zell MT, *et al.* Unambiguous Determination of the 13C and 1H NMR Stereosequence Assignments of Polylactide Using High-Resolution Solution NMR Spectroscopy. *Macromolecules* **35**, 7700-7707 (2002).
11. Becke AD. A New Mixing of Hartree-Fock and Local Density-Functional Theories. *J Chem. Phys.* **98**, 1372-1377 (1993).
12. Lee C, Yang W, Parr RG. Development of the Colle-Salvetti correlation-energy formula into a functional of the electron density. *Phys. Rev. B* **37**, 785-789 (1988).
