# Supplementary material for: Stereoselective photoredox ring-opening polymerization of O-carboxyanhydrides
Source: Nat Commun. 2018 Apr 19;9:1559. doi: 10.1038/s41467-018-03879-5 (PMC5908805; doi:10.1038/s41467-018-03879-5)
Supplement: Supplementary file 2 — Description of Additional Supplementary Files [file 41467_2018_3879_MOESM2_ESM.pdf]

Description of Additional Supplementary Files

File Name: Supplementary Data 1
Description: XYZ coordinates and Gibbs free energy (in Hartree) for all reported structures.
