# Supplementary material for: Stereoselective photoredox ring-opening polymerization of O-carboxyanhydrides
Source: Nat Commun. 2018 Apr 19;9:1559. doi: 10.1038/s41467-018-03879-5 (PMC5908805; doi:10.1038/s41467-018-03879-5)
Supplement: Supplementary file 3 — Supplementary Data 1 [file 41467_2018_3879_MOESM3_ESM.docx]

**Supplementary Data 1**

**XYZ coordinates and Gibbs free energy (in Hartree) for all reported structures**

(NNO-1)Zn/(*S*)-**5**

-1637.764774

C 3.20071300 -0.38940500 1.22335500

C 2.99054400 0.74423700 0.21770900

O 2.03304000 0.37085200 -0.74457200

O 4.12875000 -0.13425100 2.17445000

C 2.59458000 2.02470500 1.03159000

C 2.60295900 3.27486900 0.17624600

C 3.45637800 4.34379200 0.47842200

C 3.45707800 5.50076600 -0.30560800

C 2.60274700 5.60019000 -1.40496900

C 1.75287700 4.53448700 -1.71753100

C 1.75219300 3.38049300 -0.93490900

C 0.93410500 -3.40791800 -2.53527100

C 0.56999800 -2.09780400 -3.25695000

N -0.14723600 -1.15981800 -2.33330200

N 1.75426100 -3.12839400 -1.30343000

C 3.21506600 -3.05382400 -1.62939100

C 1.49770200 -4.15309700 -0.23092000

C -1.56371200 -1.62679800 -2.04352600

C -0.14318900 0.23617900 -2.88042100

C -2.21792700 -0.87312200 -0.91255300

C -1.68209700 -1.03433900 0.39467900

C -2.38161500 -0.43005100 1.47737000

C -3.52898200 0.32315900 1.20416200

C -4.04415600 0.51092200 -0.08729800

C -3.36517800 -0.11419500 -1.13824600

C -5.30528900 1.35079100 -0.37318100

C -6.38785200 0.45346900 -1.03204900

C -4.94460000 2.51460800 -1.33570400

C -5.90782300 1.96230500 0.91378000

C -1.87961400 -0.62816600 2.92222300

C -0.44368300 -0.05312200 3.06340200

C -1.85445500 -2.14761900 3.24625100

C -2.77877100 0.07326500 3.96680500

O -0.54898900 -1.75757400 0.57823300

O 2.56814500 -1.46769400 1.18466000

C 4.32620300 -1.18269900 3.20908200

H 3.98704400 0.95051900 -0.21390600

H 3.29539600 2.13549400 1.86388400

H 1.59085800 1.84741600 1.43670700

H 4.11844500 4.27132400 1.33547500

H 4.12078400 6.32136400 -0.05635900

H 2.59802500 6.49851600 -2.01209000

H 1.08967900 4.60549300 -2.57286600

H 1.12765000 2.53272600 -1.17792300

H 1.47828200 -4.07679700 -3.21678800

H 0.02496000 -3.92024100 -2.21391200

H -0.03831600 -2.32224000 -4.14732900

H 1.48184600 -1.58854000 -3.58590600

H 3.74743000 -2.75822800 -0.72439400

H 3.37780900 -2.29151100 -2.39523000

H 3.59083800 -4.02336400 -1.98688800

H 2.13605800 -3.91078100 0.62011600

H 1.70976500 -5.17074700 -0.58655400

H 0.45685300 -4.04944100 0.08511600

H -1.49439500 -2.68184700 -1.76335900

H -2.16064300 -1.54092800 -2.96391600

H -0.78384900 0.84375700 -2.23868600

H -0.52875800 0.26218400 -3.91040900

H 0.87672800 0.61884800 -2.82317600

H -4.04284400 0.78570900 2.03300100

H -3.73728000 -0.02553200 -2.15521800

H -7.28705500 1.04311900 -1.25074400

H -6.65826300 -0.36658900 -0.35799300

H -6.02089500 0.02201000 -1.96912400

H -5.83608700 3.11586600 -1.55405700

H -4.54616300 2.13158300 -2.28097800

H -4.18679800 3.15990500 -0.87828400

H -6.79518100 2.55058100 0.65274900

H -5.18990400 2.62543100 1.40907900

H -6.21153600 1.17986700 1.61824500

H -0.09329500 -0.17375000 4.09683700

H -0.43714500 1.01467400 2.81290600

H 0.22103400 -0.59082700 2.38756800

H -1.46078500 -2.30924500 4.25824800

H -1.21774600 -2.64883800 2.51567500

H -2.86887900 -2.55995300 3.19441400

H -2.37109300 -0.11773300 4.96650200

H -3.80340400 -0.31515100 3.93727800

H -2.80465800 1.15824700 3.81080900

H 5.08804100 -0.77163500 3.86793100

H 4.66310100 -2.11182900 2.74520000

H 3.39108200 -1.36010000 3.74335700

Zn 1.01871100 -1.26261500 -0.48931200

(S)-**1**

-682.880977

C 1.55450200 1.09610700 -0.03039200

C 0.95132000 -0.26457400 -0.37528500

O 2.12831700 -1.16138300 -0.38332800

O 1.01307400 2.16383900 0.15299400

C -0.07013200 -0.77420100 0.66524600

C -1.50783400 -0.41031600 0.31876600

C -2.46432600 -1.42461200 0.18197600

C -3.79098600 -1.11452000 -0.12377600

C -4.17471800 0.21592700 -0.30075300

C -3.22526100 1.23242200 -0.17068000

C -1.89979300 0.92484100 0.13934100

O 2.94623600 0.92129000 0.08843400

C 3.28427600 -0.45023900 -0.11412500

O 4.39712100 -0.89017100 -0.05497700

H 0.51508600 -0.25900700 -1.37716200

H 0.20036900 -0.40025600 1.65960000

H 0.03713400 -1.86321700 0.67713900

H -2.17089400 -2.46036300 0.31758600

H -4.52040700 -1.90976300 -0.22505800

H -5.20366600 0.45862600 -0.53919800

H -3.51579700 2.26750900 -0.30894600

H -1.16210900 1.71286000 0.22913900

CO_2_

-187.533473

C 0.00000000 0.00000000 0.00000000

O 0.00000000 0.00000000 1.18278100

O 0.00000000 0.00000000 -1.18278100

SS1

-2320.658022

C 0.41706700 -2.03573400 2.06518600

C 0.90005000 -0.60580600 1.87659900

O 0.91347700 -0.27587900 0.49398100

O 0.46311900 -2.46295400 3.34694500

C 0.03560400 0.37222500 2.74018800

C 0.62313300 1.76210400 2.65263400

C 0.29894700 2.59945200 1.57499000

C 0.90521100 3.85018300 1.43692900

C 1.85414500 4.27889100 2.37029900

C 2.18888500 3.44921400 3.44423100

C 1.57850300 2.19945400 3.58101000

C -0.00645300 -2.13758000 -3.44022500

C 0.13489600 -0.60388300 -3.40496000

N -0.76012200 -0.00362600 -2.35253900

N 0.23400800 -2.74935900 -2.07756000

C 1.66178800 -3.20411400 -1.94903500

C -0.69637900 -3.91129200 -1.82953200

C -2.22767600 -0.16632000 -2.71909800

C -0.47260100 1.46023600 -2.19044100

C -3.17531800 0.21922100 -1.61344000

C -3.21893000 -0.63149000 -0.47611800

C -4.27530300 -0.42998300 0.45909000

C -5.14055800 0.65489600 0.27420800

C -5.05138000 1.54415700 -0.80791000

C -4.06269400 1.28256200 -1.76323000

C -6.00747100 2.73939800 -0.99355600

C -6.82667300 2.54913700 -2.29897400

C -5.18503100 4.05284800 -1.08833400

C -7.00323300 2.88300700 0.18184100

C -4.49164600 -1.43857200 1.60621000

C -3.23991100 -1.48591200 2.51932300

C -4.72191100 -2.84684400 0.98924100

C -5.71460800 -1.09132000 2.48541700

O -2.30355700 -1.61806600 -0.34301900

O 0.01068800 -2.75042400 1.12726000

C -0.01774800 -3.84676600 3.59811300

H 1.91124500 -0.56778600 2.30508200

H 0.03306300 0.01052100 3.77280600

H -0.98876600 0.36160600 2.35717300

H -0.42837700 2.26107300 0.84826800

H 0.63637800 4.49043500 0.60408000

H 2.31974200 5.25245500 2.26748100

H 2.91762600 3.77717500 4.17717100

H 1.84163400 1.55786900 4.41577000

H 0.70018100 -2.55586400 -4.16886300

H -1.01387700 -2.41351500 -3.75834100

H -0.10849200 -0.19676100 -4.39930800

H 1.15879200 -0.31011100 -3.15411400

H 1.84942500 -3.49266500 -0.91446700

H 2.32539700 -2.37426300 -2.18501400

H 1.85368500 -4.05067600 -2.62347300

H -0.43914200 -4.33622600 -0.85805700

H -0.59585300 -4.67254600 -2.61469300

H -1.71597800 -3.52555600 -1.76904700

H -2.38730400 -1.22312900 -2.94657300

H -2.42334000 0.42842800 -3.62435500

H -1.11465000 1.84180300 -1.39547600

H -0.69947100 2.00114100 -3.12250500

H 0.57581300 1.60081900 -1.93851800

H -5.92327100 0.81091600 1.00135200

H -3.98755000 1.90045000 -2.65379000

H -7.50372600 3.39948000 -2.44993400

H -7.42027900 1.63053100 -2.23869600

H -6.16508700 2.47505700 -3.16831700

H -5.85544400 4.90951600 -1.23220400

H -4.48536800 4.01676500 -1.92979400

H -4.61074800 4.20518800 -0.16790000

H -7.64359000 3.75549800 0.00752900

H -6.47395400 3.02837200 1.13008900

H -7.64559100 1.99947300 0.26673100

H -3.38514000 -2.22550500 3.31823200

H -3.07019100 -0.50567700 2.97939500

H -2.38102400 -1.76389700 1.90911200

H -4.84437700 -3.59110800 1.78678300

H -3.85888700 -3.10500800 0.37315100

H -5.62647400 -2.84327600 0.37031500

H -5.81644300 -1.85475400 3.26584300

H -6.63945800 -1.08012100 1.89742100

H -5.59244400 -0.11810600 2.97547200

H 0.10969400 -3.98624200 4.66955000

H 0.58286400 -4.56010100 3.03066000

H -1.06604500 -3.93206100 3.30661700

Zn -0.36293500 -1.28775100 -0.61296800

C 3.07174300 0.72290000 -0.99731800

C 3.39694600 1.20503200 0.40175500

O 3.72671900 -0.05016600 1.11843400

O 2.72886100 1.34803100 -1.97894500

C 4.59952100 2.15694400 0.42108200

C 5.82340000 1.54547400 -0.23355100

C 6.17915700 1.89007400 -1.54399900

C 7.29124200 1.30525500 -2.15412500

C 8.05683400 0.36642600 -1.46015300

C 7.70331900 0.01115800 -0.15560000

C 6.59224600 0.59506100 0.45400100

O 3.26725400 -0.66956100 -1.02871000

C 3.45973000 -1.12951500 0.30017500

O 3.44219300 -2.28686700 0.62967600

H 2.50621700 1.63517500 0.85709700

H 4.79868700 2.42045200 1.46427300

H 4.29271000 3.06745000 -0.10471400

H 5.57909300 2.61211300 -2.08745700

H 7.55782100 1.58232500 -3.16753400

H 8.92131300 -0.08628500 -1.93171300

H 8.29164700 -0.72089300 0.38558400

H 6.30705000 0.30949000 1.45903400

SS2

-2320.661809

C 0.79680700 -0.33722900 2.56940100

C 0.66010900 0.97238800 1.81220900

O 0.84196300 0.65617400 0.40729200

O 0.80417500 -0.15435200 3.90166500

C -0.73866900 1.60671900 1.99231700

C -0.85583200 2.88627300 1.18573700

C -1.60056900 2.90748600 -0.00125300

C -1.68639000 4.07845100 -0.75868600

C -1.02905600 5.23797000 -0.34047800

C -0.28725200 5.22413700 0.84340500

C -0.20364200 4.05485700 1.60182400

C 0.66142800 -3.63580500 -2.09098600

C 0.64097900 -2.32105500 -2.89576600

N -0.28043400 -1.30500100 -2.27328200

N 1.07057300 -3.41448300 -0.65302900

C 2.56128300 -3.51390400 -0.49025800

C 0.36975100 -4.38961700 0.25399200

C -1.73702300 -1.69309300 -2.40948400

C -0.05809900 0.04509100 -2.88232900

C -2.66740900 -0.94292600 -1.48724400

C -2.58288800 -1.24901700 -0.09891100

C -3.68274900 -0.86473000 0.72636800

C -4.67484300 -0.04007700 0.17857900

C -4.68806800 0.37663700 -1.16161600

C -3.68446600 -0.13753000 -1.99370600

C -5.78614600 1.28798300 -1.74649400

C -6.59095900 0.50536000 -2.81951100

C -5.13341700 2.53442800 -2.40314600

C -6.77692300 1.78150100 -0.66602600

C -3.85965900 -1.49801900 2.12646700

C -2.65732700 -1.21124700 3.06212700

C -3.96563500 -3.03701800 1.92308500

C -5.14441100 -1.01677500 2.84029800

O -1.51540400 -1.93038300 0.36955900

O 0.85588600 -1.45363200 2.02207100

C 0.90504300 -1.37766900 4.74347500

H 1.41491100 1.67502200 2.16724700

H -0.87961600 1.79598700 3.06223200

H -1.49445600 0.89061700 1.66303800

H -2.12061700 2.01153000 -0.32353900

H -2.27193600 4.08355900 -1.67103500

H -1.09817700 6.14606700 -0.92836600

H 0.22093400 6.12143100 1.17753600

H 0.36889500 4.05248500 2.52437200

H 1.34781500 -4.34706000 -2.57138000

H -0.33372700 -4.08546000 -2.08234500

H 0.34483000 -2.53388700 -3.93470300

H 1.63572400 -1.87122200 -2.88946200

H 2.79970200 -3.35781000 0.56440200

H 3.03389900 -2.71310000 -1.05493000

H 2.92198300 -4.50136200 -0.81153100

H 0.72702100 -4.21739200 1.27141400

H 0.57126800 -5.42866400 -0.04074600

H -0.69615000 -4.15992500 0.22413000

H -1.80659700 -2.75426700 -2.15505100

H -2.04199400 -1.56127600 -3.45867100

H -0.73193800 0.75209900 -2.39497700

H -0.26020400 0.02886600 -3.96383100

H 0.97439900 0.32612200 -2.68526700

H -5.48271100 0.27723500 0.82047800

H -3.70573400 0.06336900 -3.06133200

H -7.36692600 1.14783800 -3.25425000

H -7.06960200 -0.36955500 -2.36645000

H -5.93548300 0.16017600 -3.62585400

H -5.90602600 3.18247900 -2.83524600

H -4.44403100 2.23925900 -3.20131200

H -4.57408700 3.10488100 -1.65342300

H -7.52023300 2.43883000 -1.13195600

H -6.25697700 2.34768900 0.11474600

H -7.30785600 0.94288700 -0.20229200

H -2.77748400 -1.77897500 3.99453100

H -2.61065700 -0.14665200 3.31443300

H -1.74469800 -1.52173300 2.55366900

H -4.08829800 -3.53758900 2.89207600

H -3.05231000 -3.39396500 1.44235300

H -4.82736800 -3.27504900 1.28981600

H -5.21199200 -1.51259700 3.81580400

H -6.04237000 -1.27150800 2.26641600

H -5.12500100 0.06656000 3.00906700

H 0.92599900 -0.99973600 5.76298800

H 1.82107100 -1.91825000 4.49971500

H 0.03548800 -2.01303800 4.56897300

Zn 0.35956800 -1.42769600 -0.19734000

C 2.31154800 0.45488900 -0.11687300

C 2.82122200 1.83966200 -0.58097200

O 3.55700300 2.38456300 0.57876300

O 2.22988500 -0.55558600 -0.92569600

C 3.74943200 1.74810600 -1.79874400

C 5.05399400 1.02284000 -1.52256800

C 5.23874600 -0.30484800 -1.92910800

C 6.45264300 -0.95447300 -1.70017200

C 7.49329600 -0.28753100 -1.04931700

C 7.31164500 1.03069800 -0.62552500

C 6.10025700 1.68288400 -0.86253300

O 3.12626000 0.21376000 1.13897100

C 3.83057500 1.36221700 1.48973800

O 4.53593300 1.48410000 2.46094600

H 1.97325000 2.49730200 -0.76990000

H 3.96396300 2.76797700 -2.13647400

H 3.19427600 1.23771000 -2.59094500

H 4.41762300 -0.82470300 -2.40540400

H 6.58575600 -1.98033300 -2.02521600

H 8.43574300 -0.79205100 -0.86973000

H 8.11047600 1.55066500 -0.10949200

H 5.95263700 2.70057700 -0.52241700

SS3

-2320.664062

C 0.26962300 -1.20152600 2.44805800

C 0.87949000 0.11811700 2.02311200

O 0.79225400 0.20660700 0.55987000

O 0.60696800 -1.51293900 3.70788500

C 0.12399500 1.33409900 2.60146800

C 0.87643100 2.61685400 2.30872800

C 0.70436500 3.26682200 1.07882000

C 1.42430300 4.42841900 0.79298500

C 2.32446400 4.94867400 1.72611100

C 2.50236400 4.30304900 2.95182100

C 1.78205900 3.14187700 3.23998400

C -2.31238000 -3.81817500 -1.98616700

C -1.43567200 -3.08510000 -3.01855700

N -1.05566400 -1.70718300 -2.52366900

N -1.55796300 -4.01856500 -0.69738300

C -0.52222500 -5.10135500 -0.80553400

C -2.51000100 -4.32059700 0.42694200

C -2.20253600 -0.72984700 -2.78347400

C 0.18709500 -1.22675100 -3.21771600

C -2.35642300 0.39342800 -1.78750200

C -2.77803700 0.02364300 -0.48129700

C -3.34057000 1.03253400 0.34927900

C -3.25986700 2.36583100 -0.07877500

C -2.71247300 2.75292600 -1.31363200

C -2.31181300 1.72713300 -2.18148200

C -2.66742000 4.22340200 -1.77891100

C -3.75287600 4.44003600 -2.86888800

C -1.27418900 4.55706800 -2.37534900

C -2.93305100 5.21220800 -0.61806000

C -4.16405800 0.62753400 1.59286200

C -3.31509800 -0.16522800 2.61988500

C -5.32249800 -0.28676600 1.09836100

C -4.78670800 1.84343900 2.31679900

O -2.67339900 -1.27774200 -0.11308500

O -0.45499100 -1.92871400 1.74446600

C 0.30944200 -2.92052000 4.12654800

H 1.91062100 0.12641000 2.37069400

H 0.02401500 1.17835000 3.68123300

H -0.87000900 1.35764100 2.14843600

H 0.00697300 2.86195400 0.35473200

H 1.28051100 4.92500000 -0.15966300

H 2.88144400 5.85087700 1.50107100

H 3.19627500 4.70367300 3.68197100

H 1.91611400 2.64610800 4.19657000

H -2.65230400 -4.78249800 -2.39058500

H -3.17630800 -3.19821400 -1.73554700

H -1.96948700 -3.01272700 -3.97707500

H -0.50899600 -3.63848000 -3.18907800

H 0.11956600 -5.05831700 0.07604600

H 0.11130300 -4.91091800 -1.67304900

H -1.00458700 -6.08380100 -0.90710400

H -1.92266600 -4.44624000 1.33941800

H -3.08112300 -5.23795300 0.22593800

H -3.17211400 -3.46074100 0.54088100

H -3.12152900 -1.32393600 -2.75445400

H -2.08343100 -0.33300000 -3.80096000

H 0.43037600 -0.23292600 -2.84575900

H 0.03896200 -1.20225500 -4.30701000

H 1.00721300 -1.89583900 -2.95515800

H -3.65893100 3.13258800 0.56899400

H -1.98883200 1.96227600 -3.19138800

H -3.73787600 5.47887700 -3.22236800

H -4.74484300 4.21997100 -2.45957600

H -3.57814500 3.77733300 -3.72338100

H -1.25266600 5.60108200 -2.71134900

H -1.04145600 3.91641200 -3.23146600

H -0.49496300 4.41545200 -1.61929700

H -2.83590400 6.23931700 -0.98894000

H -2.20960200 5.06510700 0.19168300

H -3.94509100 5.09479900 -0.21572600

H -3.96677500 -0.56445300 3.40792700

H -2.57593200 0.49116300 3.09190000

H -2.81127200 -0.98215100 2.10541300

H -5.93177900 -0.61608300 1.94975000

H -4.89648900 -1.15732300 0.59486300

H -5.96131000 0.26184900 0.39725200

H -5.36658600 1.48430800 3.17517300

H -5.46276700 2.39924900 1.65730600

H -4.01332800 2.52598700 2.68886300

H 0.76451900 -3.00423100 5.11046200

H 0.77329500 -3.58736800 3.39604700

H -0.76953600 -3.07476200 4.16734100

Zn -0.81978000 -2.03205100 -0.38731700

C 1.92570600 -0.01410300 -0.24938000

C 3.10863100 -0.77252200 0.36137300

O 2.66599000 -1.88571400 1.20419300

O 1.91180100 0.45593000 -1.37508500

C 4.09761500 -1.24266200 -0.73191500

C 5.17525700 -0.21672900 -1.04899000

C 6.46825000 -0.38267900 -0.53296200

C 7.47200500 0.54890100 -0.80593700

C 7.19389900 1.66206500 -1.60152800

C 5.90743300 1.83704700 -2.11762200

C 4.90358100 0.90738500 -1.84300700

O 1.20786900 -2.40684700 -0.50279500

C 1.80118300 -2.85345700 0.57264700

O 1.68193400 -3.93004700 1.16768000

H 3.63567900 -0.10861300 1.05465800

H 3.52995800 -1.51531700 -1.62417100

H 4.57183400 -2.14324900 -0.32934300

H 6.69069300 -1.25120600 0.07879000

H 8.46751900 0.40349800 -0.40185500

H 7.97173200 2.38557200 -1.81745200

H 5.68400000 2.70055800 -2.73415300

H 3.89965300 1.04784500 -2.22022200

SS4

-2320.654971

C 1.37880100 0.28559500 2.29523600

C 2.60898600 0.20793200 1.39908300

O 2.18417400 0.40344500 0.00840300

O 1.76036400 0.09592100 3.58546400

C 3.60499100 1.33853500 1.72500100

C 4.93796300 1.17859700 1.01535800

C 5.03696700 1.34570600 -0.37407900

C 6.26921700 1.20914400 -1.01385800

C 7.41652600 0.89872600 -0.27835700

C 7.32439300 0.72300000 1.10320600

C 6.09129300 0.86258500 1.74456500

C -5.29048300 -2.02843100 -1.33150600

C -4.13783500 -2.15670600 -2.34510900

N -2.98854600 -1.24382600 -1.98862200

N -4.79596700 -2.28148000 0.06391300

C -4.60344100 -3.73701900 0.36127400

C -5.70257300 -1.64454200 1.08289500

C -3.29468100 0.20412000 -2.41522400

C -1.73298200 -1.70445800 -2.67228100

C -2.49476400 1.24209000 -1.67244600

C -2.87436400 1.50983300 -0.33412800

C -2.32109600 2.64576400 0.31385400

C -1.29866000 3.33600100 -0.34036200

C -0.80156100 2.97815500 -1.60759200

C -1.46074300 1.95219900 -2.28543500

C 0.43323100 3.69353000 -2.18969700

C 0.04617200 5.12301100 -2.64704200

C 1.01939200 2.90665600 -3.38873400

C 1.54382400 3.77178000 -1.10586600

C -2.85590000 3.08531200 1.69089200

C -2.64233400 1.96092800 2.74077100

C -4.37714900 3.37383100 1.55610900

C -2.16282800 4.36787800 2.20708600

O -3.74235800 0.64678500 0.28192100

O 0.22823600 0.46574800 1.91033500

C 0.64994000 -0.13240100 4.55653600

H 3.09667700 -0.76140600 1.53088800

H 3.74769800 1.32143100 2.80900400

H 3.12847800 2.28502100 1.44848900

H 4.14604600 1.57266800 -0.94401600

H 6.33500400 1.34919200 -2.08709600

H 8.37355600 0.79796600 -0.77751800

H 8.20916400 0.48266800 1.68177900

H 6.02473300 0.73338600 2.82004800

H -6.10331200 -2.72081100 -1.59447000

H -5.68157700 -1.00832100 -1.34273700

H -4.50455000 -1.93459700 -3.35706500

H -3.74987500 -3.17974900 -2.34279700

H -4.06648500 -3.81387300 1.30854200

H -3.98978900 -4.19245900 -0.42030200

H -5.56743700 -4.26158400 0.41781300

H -5.30773100 -1.86968300 2.07676200

H -6.72831900 -2.02908900 0.99850700

H -5.66776100 -0.56401800 0.92773100

H -4.35462300 0.36583300 -2.20313000

H -3.12684300 0.27219100 -3.49824300

H -0.91019800 -1.04320100 -2.40184100

H -1.87161000 -1.70386800 -3.76313200

H -1.50002700 -2.71685800 -2.33332400

H -0.84246200 4.17508100 0.16714100

H -1.16535200 1.67736400 -3.29051700

H 0.92444500 5.64985700 -3.04151600

H -0.35886500 5.69886500 -1.80742400

H -0.71764700 5.07450400 -3.43136200

H 1.95049400 3.38451000 -3.71606000

H 0.32871500 2.90144500 -4.24021900

H 1.22717100 1.87567300 -3.08381100

H 2.44784500 4.22228500 -1.53470700

H 1.77647700 2.76514800 -0.74473600

H 1.22941200 4.38711200 -0.25650800

H -2.97832500 2.30604000 3.72745700

H -1.58718200 1.67934800 2.78516100

H -3.21876300 1.08557100 2.44126500

H -4.79355700 3.65275700 2.53251400

H -4.87684900 2.47387700 1.19128200

H -4.54801600 4.19678200 0.85231300

H -2.61190900 4.64950800 3.16665800

H -2.29508600 5.20333000 1.50957400

H -1.09083700 4.20332900 2.36734600

H 1.15171300 -0.25264300 5.51459000

H 0.12497200 -1.03796600 4.24824000

H -0.02933000 0.72145600 4.56379800

Zn -2.97032300 -1.15068700 0.13988500

C 1.31625900 -0.46764200 -0.61985500

C 0.92038600 -1.78622900 0.06096700

O -0.53653800 -1.78688500 0.28286900

O 0.95704600 -0.19405400 -1.76207900

C 1.28916100 -2.96197100 -0.86494700

C 2.79549800 -3.08888900 -1.00154900

C 3.51897900 -3.92683800 -0.14189700

C 4.90985100 -4.00757300 -0.23257000

C 5.59490100 -3.24333700 -1.17988200

C 4.88255300 -2.40166400 -2.03687300

C 3.49165000 -2.32609600 -1.95052700

O -2.21732300 -2.04686700 1.73798400

C -0.93209900 -2.14292400 1.66802700

O -0.07093000 -2.45898800 2.48843000

H 1.37813400 -1.93542600 1.03558400

H 0.82283300 -2.79221300 -1.83800300

H 0.86801600 -3.86951300 -0.42068300

H 2.98858000 -4.52071100 0.59577200

H 5.45689100 -4.66305100 0.43543600

H 6.67504900 -3.29874400 -1.24593800

H 5.40986500 -1.79883200 -2.76662300

H 2.93764100 -1.66379500 -2.60638200

SS5

-2133.120180

C 0.10364900 -1.55480900 2.68408100

C 0.01837100 -0.03936100 2.65528500

O 0.87006200 0.35555600 1.52305600

O -0.51510900 -2.06025100 3.77085500

C -1.39886200 0.52453100 2.46140700

C -1.36644800 2.03967700 2.37160800

C -1.13653500 2.81880400 3.51314900

C -1.08489300 4.21076800 3.42470200

C -1.26507300 4.83950400 2.19006000

C -1.50057000 4.06937500 1.04895100

C -1.55224400 2.67576300 1.13733500

C 1.58225500 -2.58347000 -2.76343800

C 1.78949100 -1.07122200 -2.96783100

N 0.74043000 -0.26553900 -2.24821000

N 1.63430500 -2.97503100 -1.30480400

C 3.04386600 -3.27011400 -0.87430900

C 0.73060600 -4.14532000 -1.04206800

C -0.62225400 -0.40498700 -2.87151600

C 1.13906400 1.17636000 -2.20110100

C -1.77924900 -0.04017900 -1.97040900

C -2.03539000 -0.89596500 -0.85844300

C -3.36765200 -0.89069700 -0.33674300

C -4.24381700 0.12578700 -0.74303400

C -3.92163900 1.09174900 -1.70722900

C -2.69021600 0.93944400 -2.35852200

C -4.88831700 2.21774300 -2.12521400

C -5.30213200 2.02362300 -3.60953000

C -4.18438800 3.59219600 -1.96333500

C -6.17374900 2.23631300 -1.26531100

C -3.89946600 -2.10702600 0.46192400

C -3.06569600 -2.42553400 1.72619400

C -3.81461000 -3.33251400 -0.49419900

C -5.37476000 -1.93971700 0.89586000

O -1.06718200 -1.70910200 -0.38970800

O 0.69221600 -2.24354600 1.83450500

C -0.48865200 -3.54010200 3.92504500

H 0.47727300 0.34976700 3.56750300

H -1.99542900 0.19585700 3.31937600

H -1.83636700 0.10349000 1.55505200

H -1.00076000 2.33524500 4.47588500

H -0.90763100 4.80249200 4.31554100

H -1.22595400 5.92043200 2.11955700

H -1.64906300 4.55064200 0.08908600

H -1.74850400 2.08072500 0.25176000

H 2.34882700 -3.13516600 -3.32650900

H 0.60517200 -2.88571200 -3.14633500

H 1.79503200 -0.84183100 -4.04491500

H 2.73683200 -0.77132000 -2.51972700

H 3.01075400 -3.65730500 0.14729700

H 3.59461600 -2.33148600 -0.85509100

H 3.51023400 -4.01493000 -1.53505100

H 0.83261800 -4.41849000 0.01023000

H 0.98540500 -5.00654600 -1.67586400

H -0.29370200 -3.81105600 -1.20828000

H -0.74187700 -1.45812800 -3.14217200

H -0.65746600 0.19099800 -3.79685900

H 0.37817100 1.72366200 -1.63943200

H 1.22134700 1.59973400 -3.21361800

H 2.09395000 1.22719700 -1.68307400

H -5.22804200 0.15355300 -0.30049500

H -2.44222900 1.56914000 -3.20849300

H -5.98296700 2.82575500 -3.92112400

H -5.80988800 1.06105900 -3.73460200

H -4.42608400 2.03951700 -4.26634600

H -4.85790300 4.40068100 -2.27427400

H -3.27878100 3.64138900 -2.57732400

H -3.90383200 3.75021600 -0.91626700

H -6.80733600 3.07332600 -1.58091100

H -5.93330600 2.36638300 -0.20434100

H -6.74671200 1.31062800 -1.38911100

H -3.41880400 -3.36810400 2.16514700

H -3.17392100 -1.63733100 2.47693800

H -2.02275500 -2.51763500 1.42676800

H -4.18274600 -4.23440900 0.01187900

H -2.77318700 -3.48430600 -0.78863200

H -4.42078200 -3.15507700 -1.38899200

H -5.68474200 -2.83870300 1.44179100

H -6.03826700 -1.82195500 0.03231900

H -5.49888900 -1.07631700 1.56028700

H -1.01075800 -3.72038000 4.86192200

H 0.54432100 -3.88976100 3.97003400

H -1.00684700 -4.00243200 3.08460400

Zn 0.91356000 -1.19390600 -0.27008900

C 2.22745700 0.64008700 1.90286200

C 3.10085900 0.67664000 0.65995600

O 2.77293900 -0.42201600 -0.18039000

O 2.53467600 0.75842600 3.07644100

C 4.58618800 0.67298500 1.10851400

C 5.53493500 1.14369900 0.02152500

C 6.49028100 2.13459900 0.28276900

C 7.37139400 2.55927900 -0.71521200

C 7.30612500 1.99916500 -1.99233200

C 6.35190700 1.01421900 -2.26450100

C 5.47556700 0.58915600 -1.26655900

H 2.90566800 1.65803400 0.18034200

H 4.81943600 -0.35995100 1.39550100

H 4.69511200 1.30355200 1.99603400

H 6.54406600 2.57422300 1.27349700

H 8.10494400 3.32712500 -0.49564100

H 7.98957900 2.32718100 -2.76736300

H 6.29334800 0.57792400 -3.25604900

H 4.71567800 -0.15565300 -1.45808800

SR1
-2320.651018

C -1.43507600 0.02333100 -2.31126000

C -1.75024900 0.32498400 -0.85492100

O -1.19257700 -0.71526500 -0.04097000

O -2.19534600 0.68685800 -3.19972800

C -1.18271100 1.72632900 -0.44337000

C -1.89402500 2.89704100 -1.09047300

C -3.08600500 3.38982600 -0.53947300

C -3.75678000 4.46138400 -1.13175000

C -3.24290000 5.06171400 -2.28360500

C -2.05385000 4.58227900 -2.83768900

C -1.38790400 3.50779600 -2.24611600

C 1.35174800 -4.36562000 -0.67599100

C 1.07816700 -3.92991400 0.77627300

N 1.40190400 -2.47127400 0.97164300

N 0.64128100 -3.47335000 -1.66711400

C -0.67632700 -4.07053900 -2.07563100

C 1.49065400 -3.23479700 -2.89151900

C 2.90345300 -2.23889000 0.88439400

C 0.92517800 -1.99422500 2.31115400

C 3.31281200 -0.79097600 0.82143400

C 3.01922100 -0.08892900 -0.37895600

C 3.66969000 1.16196500 -0.58484800

C 4.44119300 1.69808200 0.45304700

C 4.64909900 1.05192500 1.68131800

C 4.09262600 -0.22369100 1.82684000

C 5.47223000 1.67409500 2.82707100

C 6.66841700 0.74953000 3.17992200

C 4.56415700 1.84079700 4.07567300

C 6.03731700 3.06522400 2.45480500

C 3.58482500 1.85815600 -1.95852800

C 2.11254400 2.20609900 -2.29344400

C 4.13363900 0.88600500 -3.03984500

C 4.41120100 3.16338700 -2.01983000

O 2.19382600 -0.64698500 -1.29424500

O -0.52660100 -0.76568000 -2.65610000

C -1.91193200 0.44089200 -4.63724600

H -2.84081300 0.37555400 -0.76300700

H -0.10982900 1.74401100 -0.65479100

H -1.30736100 1.77116700 0.64379300

H -3.48445600 2.93601700 0.36220900

H -4.67452200 4.83262300 -0.68911800

H -3.75936000 5.89854700 -2.74002000

H -1.64221500 5.04874700 -3.72599900

H -0.46112700 3.14579100 -2.67611600

H 1.03617100 -5.40836500 -0.81272100

H 2.42152200 -4.31277100 -0.88814400

H 1.67785900 -4.55326200 1.45852400

H 0.02333800 -4.06402100 1.03213200

H -1.23122300 -3.32460500 -2.64503500

H -1.25312200 -4.30623800 -1.18374600

H -0.51669600 -4.97586100 -2.67853800

H 0.90344300 -2.62841700 -3.58265100

H 1.77545300 -4.18361100 -3.36584800

H 2.36347100 -2.65271200 -2.59059900

H 3.24676900 -2.72274700 -0.03345200

H 3.37534600 -2.73663200 1.74538100

H 1.10699600 -0.92054900 2.37365600

H 1.47426000 -2.50210200 3.11905400

H -0.13553600 -2.20811100 2.41598800

H 4.90748500 2.65887600 0.29439400

H 4.28209200 -0.80325300 2.72615300

H 7.25018900 1.18334800 4.00278700

H 7.32189800 0.63109100 2.30887500

H 6.32127800 -0.24180000 3.48872900

H 5.13604900 2.27272700 4.90681900

H 4.16180900 0.87402000 4.39624000

H 3.72341600 2.50359500 3.84317200

H 6.60851700 3.46143500 3.30248100

H 5.23109000 3.77044500 2.22478900

H 6.70763900 2.99952700 1.59049100

H 2.05549100 2.67117900 -3.28703400

H 1.71670300 2.91157800 -1.55393200

H 1.52666100 1.28639900 -2.28203400

H 4.05811500 1.34756400 -4.03282400

H 3.54568300 -0.03294500 -3.01594200

H 5.18578000 0.65411300 -2.83852100

H 4.32132600 3.59053200 -3.02564200

H 5.47275100 2.97390000 -1.82362200

H 4.04321500 3.90523600 -1.30120700

H -2.60582400 1.09433200 -5.16088900

H -2.09454100 -0.60843100 -4.87617700

H -0.87520800 0.69556000 -4.86466400

Zn 0.47784400 -1.50587900 -0.77331000

C -2.69320700 -2.96323100 1.61132900

C -3.65213100 -1.79686900 1.79561400

O -4.02279000 -1.45048600 0.39944700

O -2.03195900 -3.55926800 2.43562100

O -2.62253300 -3.24602500 0.24034000

C -3.29938500 -2.22018100 -0.48801900

O -3.31742000 -2.14168600 -1.68763400

C -2.93943900 -0.62855500 2.48870000

H -2.14383500 -0.32976800 1.79004500

H -2.47071800 -1.04167800 3.38983400

C -3.85655400 0.51929700 2.87097100

C -3.73264600 1.11739600 4.13250800

C -4.81997400 1.01646500 1.97818300

C -4.54643900 2.19181700 4.49651000

H -2.99358900 0.73987200 4.83160700

C -5.63878100 2.08639500 2.34665800

H -4.92803700 0.55695700 1.00416100

C -5.50351200 2.67913800 3.60431300

H -4.43552300 2.64375500 5.47548400

H -6.38219900 2.45594500 1.64929900

H -6.13811300 3.51106200 3.88653300

H -4.56243400 -2.10017100 2.31816500

SR2

-2320.656447

C -0.20238800 1.01264300 2.38209200

C -0.65670400 -0.29046900 1.73878800

O -1.20051000 0.08131700 0.42827100

O -0.04701000 0.91926800 3.71173400

C 0.48517400 -1.27095400 1.39695400

C 1.10110500 -2.09262100 2.52265500

C 0.69418100 -2.04003000 3.86080000

C 1.28116100 -2.87976600 4.81390200

C 2.28377400 -3.77550900 4.44706400

C 2.70652300 -3.82587700 3.11489300

C 2.11975500 -2.99400600 2.16393000

C 0.04421300 4.21407100 -2.36397500

C -0.67782000 3.06738400 -3.09736500

N -0.20092700 1.72082800 -2.61582400

N -0.20041000 4.16284100 -0.87680500

C -1.50419700 4.81096800 -0.51494900

C 0.94035200 4.80259700 -0.13273600

C 1.18496500 1.39491100 -3.14572700

C -1.16103900 0.64863800 -3.02872500

C 1.93713700 0.36747300 -2.33507500

C 2.38324000 0.78443500 -1.04902100

C 3.40251100 0.01261100 -0.41929200

C 3.76140200 -1.21399300 -0.99705600

C 3.21387900 -1.69875000 -2.19550200

C 2.33054300 -0.85137600 -2.87918800

C 3.59339000 -3.06648500 -2.79802100

C 4.29173300 -2.85749200 -4.16887600

C 2.30829900 -3.91483200 -2.99799700

C 4.55424900 -3.86281700 -1.88435700

C 4.19273900 0.59918300 0.77375700

C 3.27366100 0.96032500 1.96991300

C 4.87775000 1.90214300 0.27015000

C 5.29107700 -0.35936200 1.28976100

O 1.85711400 1.90380500 -0.50507700

O 0.01599300 2.06206900 1.74689300

C 0.41220900 2.14322500 4.42506400

H -1.40528800 -0.76580400 2.37379700

H 1.26698700 -0.72598900 0.85907900

H 0.03985300 -1.96422800 0.67229700

H -0.05057000 -1.32311400 4.17551800

H 0.95179300 -2.82630400 5.84553500

H 2.73553300 -4.42485100 5.18798200

H 3.48925400 -4.51507500 2.81861700

H 2.44867700 -3.03280400 1.13008400

H -0.28663800 5.18078900 -2.77041600

H 1.12259600 4.13025700 -2.51222400

H -0.52379000 3.16943700 -4.18246200

H -1.75045800 3.11427300 -2.89205700

H -1.64184000 4.72448900 0.56478500

H -2.32041700 4.27388000 -0.99501700

H -1.50450300 5.87051100 -0.80863000

H 0.71103300 4.76199000 0.93452500

H 1.08611200 5.84694500 -0.44200400

H 1.82926900 4.19780600 -0.31895600

H 1.75891400 2.32489200 -3.11525400

H 1.09196400 1.07568300 -4.19422400

H -0.76103300 -0.30780200 -2.68420500

H -1.28223400 0.62261800 -4.12181300

H -2.11067200 0.84945600 -2.53483300

H 4.51239700 -1.80808400 -0.49789700

H 1.96051200 -1.12919600 -3.86222900

H 4.55319500 -3.82621300 -4.61285000

H 5.20660100 -2.26954200 -4.03823300

H 3.63571200 -2.32455200 -4.86504300

H 2.56142900 -4.88939700 -3.43378200

H 1.60565300 -3.40843600 -3.66798700

H 1.81011300 -4.07828500 -2.03588000

H 4.76639500 -4.83558400 -2.34272000

H 4.10491900 -4.03797100 -0.90007000

H 5.50418500 -3.33371000 -1.74997900

H 3.86905300 1.46588000 2.74198900

H 2.83870700 0.05726500 2.41180200

H 2.48778900 1.62437100 1.61300100

H 5.44805200 2.36641400 1.08481300

H 4.10813100 2.59598200 -0.07581400

H 5.56098500 1.67551000 -0.55586500

H 5.82401500 0.12945100 2.11374800

H 6.02072900 -0.59583900 0.50682100

H 4.85660400 -1.29061200 1.67181600

H 0.48227300 1.83325500 5.46476600

H -0.32293300 2.93844500 4.29291800

H 1.38107000 2.45258300 4.03126400

Zn -0.16401100 2.05023100 -0.45443500

C -2.49851200 0.90197000 0.38234200

C -3.71916700 -0.01987800 0.11397400

O -4.31262600 -0.28383300 1.44840600

O -2.26995900 1.89580900 -0.42886100

O -2.73638400 1.31242800 1.84401000

C -3.77451900 0.57697700 2.40145300

O -4.15142900 0.65034900 3.54630900

C -3.39086900 -1.35950800 -0.54337900

H -2.71880200 -1.17305500 -1.38484300

H -2.84123100 -1.96051600 0.18790900

C -4.62196400 -2.10990500 -1.03280500

C -5.70293800 -2.36393900 -0.17417900

C -4.68330700 -2.57581100 -2.35226100

C -6.81647900 -3.06904900 -0.63121400

H -5.66418300 -1.98800300 0.83964000

C -5.79731300 -3.28412000 -2.80904600

H -3.85304300 -2.38586800 -3.02516500

C -6.86759000 -3.53319600 -1.94846600

H -7.64536400 -3.25660400 0.04200700

H -5.82852200 -3.63848600 -3.83320500

H -7.73337400 -4.08240900 -2.30021200

H -4.43872000 0.55497400 -0.47250800

SR3

-2320.663504

C 0.91460900 -1.34481400 2.31814200

C 1.21747900 0.07157500 1.87203200

O 0.55455400 0.24452300 0.56861800

O 1.58497800 -1.64040200 3.44372500

C 0.69484100 1.12591600 2.87153400

C 0.98867300 2.52870600 2.37862400

C 0.14146200 3.12924500 1.43536000

C 0.43028500 4.40237000 0.94147400

C 1.56428200 5.08936400 1.38289200

C 2.41116200 4.49770500 2.32347800

C 2.12531600 3.22201700 2.81651400

C -1.64934000 -4.19636200 -1.81835800

C -1.11807300 -3.25219100 -2.91284400

N -1.13584700 -1.81466900 -2.45156700

N -0.78325100 -4.12895700 -0.58959300

C 0.49999700 -4.88878600 -0.76482600

C -1.53024800 -4.62675600 0.61561400

C -2.54924500 -1.25007000 -2.54163500

C -0.21066700 -0.98958300 -3.30001700

C -2.85664400 -0.12256300 -1.58790800

C -2.93728100 -0.45924800 -0.20962600

C -3.60405500 0.44932500 0.65685900

C -3.95400400 1.71223800 0.15852200

C -3.73849500 2.10586400 -1.17151900

C -3.22887400 1.13735900 -2.04655400

C -4.07400900 3.51764700 -1.69259800

C -5.09739400 3.42286200 -2.85653900

C -2.77202200 4.19146700 -2.20478700

C -4.68270500 4.42043500 -0.59390900

C -4.04135700 -0.01306800 2.06248000

C -2.82205700 -0.42293100 2.92380400

C -4.96703200 -1.25116300 1.88556600

C -4.83211200 1.07225900 2.82889500

O -2.42310300 -1.64499200 0.20816300

O 0.17486000 -2.15017400 1.72831300

C 1.65747500 -3.09152200 3.79404500

H 2.29733200 0.14352500 1.76369900

H 1.18554200 0.92973000 3.83026700

H -0.38034600 0.98466000 2.99116900

H -0.73421700 2.59622900 1.08361000

H -0.23020300 4.85107700 0.20827400

H 1.78513900 6.07889000 0.99957500

H 3.28933300 5.02763400 2.67423900

H 2.78047900 2.76788200 3.55361200

H -1.70235500 -5.22644000 -2.20015600

H -2.64803400 -3.87871200 -1.51011100

H -1.71656600 -3.36957500 -3.82844000

H -0.08031200 -3.49972000 -3.14886100

H 1.18828900 -4.61839500 0.03735600

H 0.97325900 -4.58632900 -1.69936600

H 0.30593800 -5.97066400 -0.77884900

H -0.86460900 -4.54586800 1.47659800

H -1.84930200 -5.67024300 0.48391200

H -2.38615200 -3.96711000 0.77012000

H -3.22825300 -2.07655300 -2.31038100

H -2.72718400 -0.93960200 -3.58112000

H -0.26896700 0.04473600 -2.96357800

H -0.49048000 -1.05814500 -4.36144900

H 0.80512500 -1.35239500 -3.14245800

H -4.42523600 2.41166800 0.83308900

H -3.13327700 1.35645500 -3.10612800

H -5.33058600 4.42528400 -3.23679100

H -6.02332200 2.95398800 -2.50607700

H -4.69949400 2.82561500 -3.68330500

H -2.98992000 5.19328300 -2.59656500

H -2.31163500 3.59586900 -2.99949400

H -2.04864900 4.28070900 -1.38649100

H -4.89409700 5.41002500 -1.01565900

H -3.98709500 4.54608400 0.24332900

H -5.62156900 4.00231600 -0.21393800

H -3.16090900 -0.83443700 3.88332700

H -2.20208700 0.45648000 3.12690400

H -2.23955600 -1.16657400 2.38017300

H -5.28737000 -1.62572000 2.86634100

H -4.41577500 -2.03237100 1.35804300

H -5.85534400 -0.97732600 1.30535800

H -5.13163100 0.66919200 3.80352900

H -5.73874100 1.36366000 2.28677700

H -4.22022800 1.96561500 3.00267800

H 2.28815500 -3.12049500 4.67932400

H 2.11475100 -3.60337800 2.94495200

H 0.65902100 -3.47991900 3.99805800

Zn -0.53683700 -2.00776800 -0.34862400

C 1.29283000 0.66885600 -0.56404200

C 2.77515400 0.29927800 -0.70990300

O 3.19197900 -0.82689100 0.11042900

O 0.70793900 1.30982300 -1.41722700

O 1.44754100 -1.92072000 -0.91774000

C 2.43311300 -2.04896900 -0.07777200

O 2.78311400 -3.00749000 0.62016500

C 3.67597700 1.50742700 -0.35885100

H 3.22274100 2.39251700 -0.81796500

H 3.65950600 1.65535700 0.72620900

C 5.10388200 1.34806200 -0.85658800

C 5.90082200 0.27184300 -0.43561600

C 5.64880400 2.29157300 -1.73703900

C 7.21499100 0.15009800 -0.88743200

H 5.46923700 -0.47177900 0.22022900

C 6.96541500 2.16923600 -2.18774800

H 5.04035500 3.12693100 -2.06809900

C 7.75285400 1.09807500 -1.76246800

H 7.81975100 -0.68711500 -0.55742100

H 7.37290200 2.90792200 -2.86869200

H 8.77493300 1.00138700 -2.11037800

H 2.89275400 0.06647900 -1.77124700

SR4

-2320.656706

C -1.48561100 -2.58223200 -0.71751400

C -2.49512500 -1.48699900 -0.39395000

O -2.02712600 -0.19088400 -0.89768800

O -2.02932000 -3.77812600 -0.38065600

C -3.85336700 -1.77268400 -1.07891400

C -4.97436300 -0.89929200 -0.54356300

C -5.01637900 0.47677000 -0.81405800

C -6.05342700 1.26087500 -0.30588400

C -7.06067700 0.68478600 0.47211300

C -7.02383900 -0.68353400 0.74724000

C -5.98514500 -1.46866400 0.24305500

C 4.21358700 -2.37062900 -0.61055400

C 4.26489800 -1.64215800 0.74241600

N 3.15300300 -0.62841800 0.83551300

N 2.92909800 -3.14870100 -0.75745500

C 2.93345100 -4.38410100 0.08828900

C 2.71701600 -3.49844600 -2.20619300

C 3.54172500 0.65615100 0.09832500

C 2.83881300 -0.32264500 2.26999100

C 2.41109800 1.40645300 -0.56900300

C 1.79984000 0.77139700 -1.68500800

C 1.00492900 1.56547200 -2.55590400

C 0.72435500 2.88251300 -2.17898300

C 1.22200800 3.48559200 -1.01174100

C 2.10753700 2.72780200 -0.23135400

C 0.78096100 4.90176400 -0.58700000

C 1.98445700 5.73189500 -0.07030500

C -0.27816300 4.76019200 0.54078300

C 0.13686600 5.68655600 -1.75719900

C 0.54892900 1.00433100 -3.91897400

C -0.35073900 -0.24467800 -3.73602100

C 1.82151900 0.60370400 -4.71786500

C -0.23549600 2.04387100 -4.75265100

O 2.01731400 -0.55510400 -1.87407400

O -0.33168700 -2.47803900 -1.15654800

C -1.09819500 -4.93771600 -0.33656300

H -2.60866100 -1.48402500 0.69444300

H -4.08849800 -2.82518600 -0.90712400

H -3.71367200 -1.61217100 -2.15340700

H -4.22303900 0.93844900 -1.38598500

H -6.07096500 2.32404400 -0.51624200

H -7.86572800 1.29702300 0.86206600

H -7.80001100 -1.13875200 1.35188100

H -5.95923800 -2.53220900 0.45755000

H 5.07950900 -3.03983300 -0.72056700

H 4.20544800 -1.63651900 -1.41843600

H 5.24439500 -1.15804000 0.86761900

H 4.13272000 -2.35232700 1.56336000

H 2.00985700 -4.93406900 -0.09957800

H 2.95148000 -4.11066300 1.14317500

H 3.79421500 -5.02341200 -0.15652000

H 1.78129000 -4.05494800 -2.29092400

H 3.54725600 -4.10580000 -2.59384900

H 2.62233700 -2.56112200 -2.75594800

H 4.24774300 0.35841800 -0.68321400

H 4.07284700 1.30597000 0.80726300

H 2.06785900 0.44418400 2.30597800

H 3.73152900 0.03407600 2.80281300

H 2.45178900 -1.22729600 2.74094300

H 0.08730900 3.46699000 -2.82414800

H 2.59126400 3.16695700 0.63587100

H 1.64342900 6.73100800 0.22666500

H 2.74133100 5.83748400 -0.85542400

H 2.45172500 5.26334700 0.80200100

H -0.70534900 5.73809300 0.79687400

H 0.18998300 4.34537500 1.43987700

H -1.07489000 4.07805600 0.22682700

H -0.09546300 6.70376500 -1.42124400

H -0.79778400 5.21965500 -2.08431600

H 0.82189300 5.74949400 -2.61006300

H -0.64236100 -0.63439100 -4.72049200

H -1.24898600 0.01001800 -3.16735300

H 0.20170400 -1.00300500 -3.18383300

H 1.53558900 0.16291500 -5.68139200

H 2.39073700 -0.12541900 -4.13760100

H 2.44471000 1.48596000 -4.90463500

H -0.50557800 1.59099800 -5.71378900

H 0.36711500 2.93735300 -4.95329900

H -1.16049100 2.34271800 -4.24577300

H -1.70714000 -5.75595000 0.04078600

H -0.28388000 -4.68816200 0.34644400

H -0.71638500 -5.15286500 -1.33625000

Zn 1.50359600 -1.59552500 -0.23229600

C -1.70632700 0.87285100 -0.04698300

C -0.68200000 0.66920400 1.07345400

O 0.00444100 -0.61244500 0.97226000

O -2.19121800 1.97272600 -0.26628300

O 0.66646400 -2.66482600 1.49449200

C -0.27239300 -1.84428800 1.79187200

O -1.29348400 -1.90180600 2.48157100

C -1.31781400 1.01184600 2.45231300

H -1.81823900 1.97369200 2.29903000

H -2.05858400 0.25622000 2.70660300

C -0.29310700 1.13017300 3.57028500

C -0.10811800 0.08847300 4.49194000

C 0.47944300 2.29334400 3.70285700

C 0.82995100 0.21160900 5.51979800

H -0.68942700 -0.81747100 4.38077700

C 1.41743500 2.41598400 4.72930900

H 0.33681900 3.11001700 3.00447900

C 1.59612500 1.37305700 5.64214500

H 0.95873500 -0.59993000 6.22734900

H 2.00038200 3.32561900 4.82124100

H 2.31952200 1.46852100 6.44390500

H 0.09825000 1.40917600 0.86347100

SR5

-2133.116201

C 0.80227300 -1.39665100 2.50449900

C 1.14766800 0.02932600 2.10492000

O 1.60912200 -0.07681100 0.71741700

O 0.55393000 -1.49653900 3.82538200

C -0.03705800 1.00784300 2.15187400

C 0.35234800 2.36390300 1.59024500

C 1.27688400 3.17972700 2.25617500

C 1.64070000 4.41968100 1.72689200

C 1.07737300 4.86059000 0.52571400

C 0.14775700 4.05692800 -0.13792500

C -0.21364000 2.81471100 0.39038700

C -0.30292500 -3.72881600 -2.61094700

C 0.10576500 -2.41488200 -3.30401900

N -0.39693300 -1.21058400 -2.55115000

N 0.21609300 -3.80916200 -1.19425200

C 1.58592400 -4.42672900 -1.15892800

C -0.74063700 -4.55802200 -0.31384000

C -1.89398300 -1.07453500 -2.59339100

C 0.24148900 0.03130500 -3.08759100

C -2.47047000 -0.16445400 -1.53356500

C -2.40984400 -0.61251000 -0.18098300

C -3.38256300 -0.06996700 0.71738700

C -4.10453200 1.06362500 0.31759100

C -4.00135100 1.64141800 -0.95629800

C -3.22253900 0.95108100 -1.89466300

C -4.74405000 2.92965900 -1.36052900

C -5.71145200 2.63882700 -2.53900800

C -3.70425100 3.99679400 -1.80027000

C -5.57073900 3.52041700 -0.19412100

C -3.78967600 -0.84787200 1.99327700

C -2.61148800 -1.08457900 2.96671200

C -4.31403200 -2.23497800 1.51942100

C -4.92497400 -0.15189300 2.77934700

O -1.51557200 -1.55414100 0.18860300

O 0.77318100 -2.35321200 1.71341100

C 0.21213500 -2.85133600 4.33652100

H 1.97579000 0.36397200 2.73207300

H -0.35036400 1.09078500 3.19816400

H -0.86583700 0.59237000 1.57778100

H 1.71263200 2.84660100 3.19329500

H 2.35482200 5.04304500 2.25314600

H 1.35796100 5.82345900 0.11483100

H -0.30130800 4.39549000 -1.06464700

H -0.94336800 2.19651000 -0.12222900

H 0.07289200 -4.57987000 -3.19661400

H -1.39125400 -3.81117500 -2.56587700

H -0.26777800 -2.41554400 -4.34032600

H 1.19355100 -2.32019600 -3.29635000

H 1.87979400 -4.53567800 -0.11164000

H 2.27299200 -3.72430600 -1.62952500

H 1.58745100 -5.41137600 -1.64800100

H -0.30716600 -4.60166100 0.68703800

H -0.92740800 -5.57527100 -0.68632600

H -1.66236100 -3.97817700 -0.25693000

H -2.31380000 -2.07188200 -2.43301000

H -2.19852400 -0.73205200 -3.59487900

H -0.14192800 0.88307700 -2.52066400

H 0.00923900 0.16901100 -4.15488100

H 1.31099900 -0.07363800 -2.92511400

H -4.79038600 1.50443300 1.02555900

H -3.21808500 1.26359500 -2.93530500

H -6.23503600 3.55717500 -2.83291300

H -6.45245700 1.88909000 -2.24154200

H -5.16732700 2.25992900 -3.41027400

H -4.21200300 4.92237700 -2.09981300

H -3.11302500 3.63499200 -2.64849600

H -3.02226600 4.22064800 -0.97233800

H -6.05538200 4.44531800 -0.52770100

H -4.92923300 3.75757900 0.66179400

H -6.35193100 2.82389800 0.13069600

H -2.93691100 -1.74378400 3.78230900

H -2.27264600 -0.14072900 3.40400000

H -1.80180700 -1.55102000 2.40833000

H -4.62623500 -2.83649200 2.38282600

H -3.51480200 -2.75588500 0.98659600

H -5.17083100 -2.10558600 0.84940900

H -5.17982500 -0.76743400 3.65033500

H -5.82639900 -0.03927100 2.16724400

H -4.61302400 0.83560200 3.13962800

H 0.08376900 -2.70881200 5.40705600

H 1.02683200 -3.54483300 4.12087700

H -0.70991900 -3.19495900 3.86678600

Zn 0.37531500 -1.72460400 -0.56545900

C 2.97555600 -0.54026900 0.62368700

C 3.27545300 -0.85745700 -0.82545000

O 2.19434300 -1.56136700 -1.39825600

O 3.64757400 -0.67706500 1.62850100

C 3.57208600 0.49150900 -1.57073700

H 2.67930800 1.11799800 -1.49000300

H 3.71570900 0.22163600 -2.62312900

C 4.78421800 1.21499500 -1.02940800

C 6.07702900 0.81053100 -1.39450300

C 4.64349200 2.27801900 -0.12547800

C 7.20121600 1.44816800 -0.86897600

H 6.19922600 -0.00564400 -2.09976300

C 5.76762700 2.91812100 0.40085600

H 3.65085800 2.60980500 0.16210100

C 7.04947400 2.50526800 0.03218100

H 8.19320400 1.12452900 -1.16387000

H 5.64111800 3.74042200 1.09622900

H 7.92183100 3.00325800 0.43971700

H 4.21887100 -1.42690000 -0.80689500

TS_SS1_

-2320.656653

C 3.35026646 -2.17864796 -1.38607896

C 3.14637922 -1.00116494 -0.44836863

O 1.74380880 -0.96489308 -0.05829151

O 4.59118857 -2.10217943 -1.94452489

C 4.04824134 -1.10394806 0.80461901

C 5.39853917 -0.44915277 0.57969241

C 5.61258834 0.89113452 0.93645059

C 6.84754621 1.49717016 0.69956381

C 7.88320449 0.77273409 0.10312537

C 7.67753107 -0.56042315 -0.25569436

C 6.44139265 -1.16646270 -0.01976380

C -0.75788328 -4.54495133 0.99798690

C -0.94308706 -4.36446699 -0.52027900

N -1.43669818 -2.97428986 -0.81032311

N 0.29305749 -3.59730982 1.54492688

C 1.64594798 -4.23780626 1.42893252

C 0.00516101 -3.31058683 3.00245186

C -2.90156523 -2.81009930 -0.40638487

C -1.25209886 -2.59274090 -2.25153681

C -3.32749266 -1.39268430 -0.10687292

C -2.77188126 -0.75000396 1.03230352

C -3.31561033 0.50247533 1.42412311

C -4.31980740 1.07582412 0.63400854

C -4.83426340 0.47273358 -0.52283498

C -4.32573252 -0.78500476 -0.86462118

C -5.92230181 1.12737996 -1.39808768

C -7.18292291 0.22106847 -1.42561757

C -5.38168327 1.30455253 -2.84282374

C -6.34206807 2.51927061 -0.87049331

C -2.82441207 1.19198584 2.71428821

C -1.29776893 1.45933670 2.63470717

C -3.11656399 0.25621846 3.92000357

C -3.53097068 2.54295287 2.97494724

O -1.74147253 -1.34967686 1.69611026

O 2.56050306 -3.10037948 -1.60404789

C 4.92693575 -3.15807432 -2.93029874

H 3.41370574 -0.08235997 -0.97200447

H 4.17371619 -2.16907058 1.02281197

H 3.52857398 -0.63518958 1.64434868

H 4.80651165 1.45833637 1.38437331

H 6.99930608 2.53375108 0.97718248

H 8.84046837 1.24527287 -0.08268357

H 8.47558267 -1.12747375 -0.72079723

H 6.28528359 -2.20252932 -0.30207120

H -0.47757017 -5.58204414 1.22821670

H -1.68835310 -4.31480900 1.51814215

H -1.64481035 -5.12097170 -0.90119855

H 0.01456573 -4.47128134 -1.03445983

H 2.39006029 -3.55233827 1.83680938

H 1.87346480 -4.42097806 0.38158020

H 1.67256530 -5.17602943 2.00113591

H 0.81225699 -2.68469369 3.39362315

H -0.04239587 -4.24269059 3.58184201

H -0.93184970 -2.75411075 3.04515657

H -3.04955388 -3.41084640 0.49437299

H -3.51961395 -3.23508335 -1.20947242

H -1.59695626 -1.56462408 -2.36447327

H -1.82398347 -3.26261319 -2.90923641

H -0.18826724 -2.63301863 -2.48941789

H -4.71469722 2.03583685 0.92942367

H -4.71739201 -1.31297683 -1.72946606

H -7.96008131 0.67250804 -2.05514281

H -7.57835254 0.09557225 -0.41170891

H -6.94474151 -0.76919709 -1.82783101

H -6.14733096 1.76461590 -3.48015388

H -5.10401642 0.33893624 -3.27788272

H -4.49420031 1.94635198 -2.83610281

H -7.10674613 2.94082162 -1.53315657

H -5.48846318 3.20595509 -0.85102923

H -6.76402379 2.44997285 0.13838928

H -0.95555741 1.95412259 3.55285840

H -1.07100020 2.10406031 1.77841034

H -0.78463171 0.50603465 2.51015804

H -2.74404914 0.70893414 4.84801186

H -2.61966568 -0.70093574 3.75370906

H -4.19604239 0.09301136 4.01697824

H -3.14507886 2.96762024 3.90915993

H -4.61427081 2.41468524 3.08119678

H -3.33552236 3.25877408 2.16758900

H 4.92678809 -4.13927883 -2.45014879

H 5.91986970 -2.89291377 -3.28640416

H 4.19966817 -3.15020480 -3.74440206

Zn -0.24866220 -1.75325906 0.44024476

C 1.21717352 2.26231411 0.09679347

C 0.86985829 0.83756236 0.36172473

O -0.20369519 0.17666904 -0.33419406

O 1.44115876 0.43207534 1.57481739

C 2.11845759 1.54908408 2.07836262

O 2.82568016 1.46496986 3.16746442

O 1.87847464 2.60545674 1.25586247

H 0.39772669 2.89857196 0.35867672

C 1.58426956 2.37000006 -1.39493178

H 0.69071912 2.36339288 -1.98350968

H 2.19976022 1.53962956 -1.67161947

C 2.35156369 3.68230800 -1.64132089

C 3.72767946 3.65345963 -1.86923445

C 1.67080952 4.89972370 -1.63674695

C 4.42291522 4.84186769 -2.09184457

H 4.26420933 2.69357955 -1.87201354

C 2.36594624 6.08849619 -1.86038682

H 0.58618683 4.92262718 -1.45730877

C 3.74181087 6.05977446 -2.08778207

H 5.50768916 4.81926441 -2.27087278

H 1.82886894 7.04818968 -1.85713090

H 4.29018223 6.99665711 -2.26338419

TS_SS2_

-2320.646894

C 1.05917600 -1.02889100 1.98361300

C 1.10697600 0.33347100 1.30912900

O 1.00446800 0.10599900 -0.13526500

O 1.58813600 -1.03593900 3.21034600

C -0.05942500 1.23485100 1.77562200

C -0.00977500 2.60355500 1.12282500

C -1.01714500 2.99720900 0.23318000

C -0.97568800 4.25877300 -0.36615300

C 0.07204600 5.13758000 -0.08428200

C 1.07727500 4.75355000 0.80741300

C 1.03307700 3.49528600 1.40982800

C -0.57810800 -3.30025900 -2.91560300

C -0.42465300 -1.84494500 -3.39417800

N -1.07347900 -0.87741600 -2.43489100

N 0.00034500 -3.49470700 -1.52419900

C 1.38516500 -4.08486200 -1.62496800

C -0.86918700 -4.42635600 -0.70831300

C -2.58294600 -1.05837400 -2.37791700

C -0.78433400 0.53793000 -2.86081400

C -3.23836700 -0.31429500 -1.24154900

C -3.01009300 -0.81791100 0.06750300

C -3.85078000 -0.34488800 1.11676100

C -4.73336500 0.70812200 0.84203000

C -4.87713800 1.29048500 -0.42693500

C -4.13676500 0.72453500 -1.47323900

C -5.83167200 2.46909300 -0.70493400

C -6.90233500 2.04174400 -1.74498600

C -5.01806000 3.66688900 -1.26720900

C -6.56507200 2.94480800 0.57105100

C -3.86890600 -1.06929500 2.48214700

C -2.48193000 -1.01796900 3.17235500

C -4.24366500 -2.55723100 2.22830100

C -4.90933900 -0.47140800 3.45753700

O -2.05027300 -1.75198500 0.26137200

O 0.58050600 -2.03814700 1.42529800

C 1.81530200 -2.38625400 3.82088500

H 2.05470800 0.79299200 1.57623600

H 0.01164400 1.32068700 2.86731200

H -1.00495200 0.74278300 1.53605200

H -1.83817900 2.32042600 0.01992800

H -1.76453400 4.55228200 -1.04927200

H 0.10371700 6.11556300 -0.55043100

H 1.88839900 5.43472600 1.03820700

H 1.80663200 3.21048300 2.11711000

H -0.07414000 -3.97259100 -3.62094100

H -1.63242400 -3.58140800 -2.88452100

H -0.85957100 -1.74385300 -4.40042800

H 0.63230600 -1.56147800 -3.43206600

H 1.83835400 -4.11220000 -0.63390900

H 2.00200500 -3.44664900 -2.25341400

H 1.32029300 -5.09723500 -2.04769400

H -0.37024600 -4.59137200 0.24938400

H -1.00639200 -5.38556100 -1.22445600

H -1.82001500 -3.92732900 -0.51648200

H -2.78217300 -2.12320900 -2.23578900

H -3.00353100 -0.74288100 -3.34390300

H -1.26025100 1.20903300 -2.14606800

H -1.19957500 0.71747300 -3.86408200

H 0.29228000 0.68812500 -2.84497200

H -5.34307200 1.08267700 1.65010200

H -4.27136700 1.07972900 -2.49097300

H -7.57908300 2.87902700 -1.95657500

H -7.48939900 1.20229500 -1.35710300

H -6.43475200 1.73074100 -2.68492100

H -5.68351600 4.51447900 -1.47359400

H -4.51082000 3.39026700 -2.19766600

H -4.26189100 3.98260900 -0.53949400

H -7.21772500 3.78829500 0.31824400

H -5.85337300 3.27752400 1.33475400

H -7.18658300 2.14568900 0.99022800

H -2.51023500 -1.60067500 4.10228600

H -2.21915700 0.01615300 3.42226300

H -1.73974100 -1.44335300 2.49746200

H -4.25304900 -3.10843000 3.17713500

H -3.50429500 -2.99800500 1.55718600

H -5.23751900 -2.62343000 1.77158500

H -4.88518400 -1.04375900 4.39222700

H -5.92357300 -0.53196700 3.04702200

H -4.68234000 0.57511300 3.69338900

H 2.26290900 -2.16650900 4.78700700

H 2.49719100 -2.92604700 3.16107700

H 0.86587800 -2.91259200 3.92338700

Zn -0.23679000 -1.57479800 -0.55699200

C 2.34081700 -0.00526300 -1.05168000

C 3.57720600 0.46642700 -0.27232700

O 3.81122200 -0.55377100 0.77424800

O 2.12181700 0.25012900 -2.23668300

C 4.77219000 0.55681400 -1.22988400

C 6.00385000 1.19952100 -0.61532200

C 6.54973700 0.71751300 0.58501400

C 7.69205600 1.30661700 1.12747300

C 8.30691800 2.38248500 0.48129100

C 7.76814700 2.87039800 -0.71047200

C 6.62309900 2.28235400 -1.25308600

O 2.56759900 -1.65914100 -0.77889600

C 3.28954700 -1.80477500 0.34269900

O 3.47447600 -2.82698800 0.98654600

H 3.40786900 1.41756900 0.23203900

H 4.43565400 1.12803600 -2.10026900

H 4.99164200 -0.46052600 -1.57706300

H 6.05759900 -0.10115700 1.09305100

H 8.10317800 0.92536000 2.05552600

H 9.19603200 2.83662700 0.90350900

H 8.23632100 3.70685800 -1.21697800

H 6.20705700 2.66395000 -2.17980500

TS_SS3_

-2320.654706

C 2.30706000 -1.71682000 -1.95430500

C 2.55310700 -1.71293200 -0.45902300

O 1.33328700 -1.18082800 0.15272300

O 3.24717900 -2.46044300 -2.58599600

C 2.80256300 -3.14937600 0.06261600

C 3.05012200 -3.15194200 1.55717200

C 1.97019500 -3.17157000 2.45139300

C 2.19229300 -3.09982300 3.82855100

C 3.49430200 -3.00365200 4.32684600

C 4.57556800 -2.98371000 3.44183900

C 4.35347400 -3.05566200 2.06475200

C -0.45888500 2.76779200 -3.47940000

C -0.49476500 3.27231400 -2.02910200

N -1.03967600 2.22439400 -1.09801400

N 0.33014000 1.48521400 -3.57962900

C 1.77408000 1.78408800 -3.87412500

C -0.24590600 0.57390900 -4.62676200

C -2.50235400 1.93018200 -1.37793500

C -0.86833200 2.67658400 0.32107600

C -3.05392400 0.75290400 -0.61291700

C -2.53096200 -0.53502100 -0.89151700

C -3.20263500 -1.65973200 -0.31984100

C -4.28518100 -1.43282100 0.53014700

C -4.77199800 -0.15335000 0.85273500

C -4.13960800 0.93252600 0.24884800

C -5.96175200 -0.00638800 1.82101500

C -5.58645400 -0.62047000 3.19697300

C -7.19887000 -0.74711700 1.24521900

C -6.34987100 1.47368600 2.04765500

C -2.73525300 -3.09208400 -0.64958300

C -1.30253600 -3.29941200 -0.09198600

C -2.73024400 -3.29605200 -2.19006100

C -3.64995300 -4.17640900 -0.03266300

O -1.45216500 -0.67724900 -1.69865300

O 1.40155200 -1.13005200 -2.56195700

C 3.14132200 -2.55747600 -4.06446700

H 3.40037200 -1.04926400 -0.25784200

H 3.66098400 -3.55251800 -0.47939500

H 1.91970900 -3.75094100 -0.17658000

H 0.95969200 -3.23454100 2.06445400

H 1.34964000 -3.11799700 4.50992100

H 3.66547900 -2.94817500 5.39550200

H 5.58805600 -2.91333700 3.82224200

H 5.19539500 -3.03980000 1.37980600

H -0.01798900 3.53702900 -4.12827000

H -1.47074600 2.56936200 -3.83882300

H -1.09742700 4.19195100 -1.97198700

H 0.52047100 3.49146800 -1.68727700

H 2.31531100 0.83729800 -3.87901100

H 2.19894200 2.40879300 -3.08702900

H 1.87204600 2.27473600 -4.85338200

H 0.38162000 -0.31671200 -4.67250800

H -0.28698700 1.05955100 -5.61180800

H -1.24237400 0.26610200 -4.30031800

H -2.58463300 1.71325600 -2.44697600

H -3.08729400 2.83623800 -1.15793200

H -1.25681900 1.90187800 0.97945000

H -1.40281900 3.62147000 0.49473100

H 0.19390600 2.81185300 0.51754100

H -4.77890800 -2.28890200 0.97054500

H -4.49133500 1.94222600 0.42731700

H -6.42849400 -0.53000800 3.89499100

H -4.72017100 -0.09837700 3.61758400

H -5.33290600 -1.68053900 3.09538500

H -8.04769400 -0.66066200 1.93534300

H -6.98292400 -1.80950300 1.09354100

H -7.48197600 -0.31247000 0.28027700

H -7.19310800 1.52419000 2.74630700

H -6.65428100 1.95060900 1.10900900

H -5.51388000 2.03752200 2.47680900

H -0.92933100 -4.29307700 -0.37800200

H -1.31615200 -3.22952700 1.00285300

H -0.64433400 -2.52569900 -0.48222800

H -2.35766900 -4.30007400 -2.43194300

H -2.08887700 -2.53888900 -2.64191700

H -3.74862000 -3.19574200 -2.58379000

H -3.27262500 -5.16309300 -0.32665200

H -4.68024500 -4.08204400 -0.39519700

H -3.65203800 -4.12713200 1.06251400

H 3.23066100 -1.56543600 -4.51087400

H 2.18333200 -3.00207900 -4.34045700

H 3.97370600 -3.19571300 -4.35166800

Zn 0.12002300 0.47696400 -1.62713400

C 1.39636800 -0.19019100 1.16047600

C 2.78163800 0.28206100 1.60474100

O 3.59492700 0.88902800 0.54791800

O 0.34282500 0.14112200 1.67529300

C 2.71525500 1.17790600 2.85575300

C 2.07454300 2.54541900 2.69175200

C 0.85802700 2.83473700 3.32530600

C 0.30518100 4.11454700 3.25672400

C 0.95518200 5.11960000 2.53536500

C 2.15631100 4.83368300 1.88248600

C 2.72005900 3.55753700 1.96498000

O 1.84276200 1.20871700 -0.94560000

C 2.95824000 1.68303000 -0.50419200

O 3.60288900 2.65081200 -0.90763600

H 3.33136000 -0.62124800 1.88921700

H 3.75253400 1.31100200 3.18346100

H 2.19157900 0.61296700 3.63351100

H 0.34217700 2.04763000 3.86262100

H -0.63077900 4.32521800 3.76194700

H 0.52912300 6.11523700 2.48287200

H 2.66282100 5.60376700 1.31185800

H 3.64209100 3.34414200 1.43979400

TS_SR1_

-2320.644266

C 3.35026646 -2.17864796 -1.38607896

C 3.14637922 -1.00116494 -0.44836863

O 1.74380880 -0.96489308 -0.05829151

O 4.59118857 -2.10217943 -1.94452489

C 4.04824134 -1.10394806 0.80461901

C 5.39853917 -0.44915277 0.57969241

C 5.61258834 0.89113452 0.93645059

C 6.84754621 1.49717016 0.69956381

C 7.88320449 0.77273409 0.10312537

C 7.67753107 -0.56042315 -0.25569436

C 6.44139265 -1.16646270 -0.01976380

C -0.75788328 -4.54495133 0.99798690

C -0.94308706 -4.36446699 -0.52027900

N -1.43669818 -2.97428986 -0.81032311

N 0.29305749 -3.59730982 1.54492688

C 1.64594798 -4.23780626 1.42893252

C 0.00516101 -3.31058683 3.00245186

C -2.90156523 -2.81009930 -0.40638487

C -1.25209886 -2.59274090 -2.25153681

C -3.32749266 -1.39268430 -0.10687292

C -2.77188126 -0.75000396 1.03230352

C -3.31561033 0.50247533 1.42412311

C -4.31980740 1.07582412 0.63400854

C -4.83426340 0.47273358 -0.52283498

C -4.32573252 -0.78500476 -0.86462118

C -5.92230181 1.12737996 -1.39808768

C -7.18292291 0.22106847 -1.42561757

C -5.38168327 1.30455253 -2.84282374

C -6.34206807 2.51927061 -0.87049331

C -2.82441207 1.19198584 2.71428821

C -1.29776893 1.45933670 2.63470717

C -3.11656399 0.25621846 3.92000357

C -3.53097068 2.54295287 2.97494724

O -1.74147253 -1.34967686 1.69611026

O 2.56050306 -3.10037948 -1.60404789

C 4.92693575 -3.15807432 -2.93029874

H 3.41370574 -0.08235997 -0.97200447

H 4.17371619 -2.16907058 1.02281197

H 3.52857398 -0.63518958 1.64434868

H 4.80651165 1.45833637 1.38437331

H 6.99930608 2.53375108 0.97718248

H 8.84046837 1.24527287 -0.08268357

H 8.47558267 -1.12747375 -0.72079723

H 6.28528359 -2.20252932 -0.30207120

H -0.47757017 -5.58204414 1.22821670

H -1.68835310 -4.31480900 1.51814215

H -1.64481035 -5.12097170 -0.90119855

H 0.01456573 -4.47128134 -1.03445983

H 2.39006029 -3.55233827 1.83680938

H 1.87346480 -4.42097806 0.38158020

H 1.67256530 -5.17602943 2.00113591

H 0.81225699 -2.68469369 3.39362315

H -0.04239587 -4.24269059 3.58184201

H -0.93184970 -2.75411075 3.04515657

H -3.04955388 -3.41084640 0.49437299

H -3.51961395 -3.23508335 -1.20947242

H -1.59695626 -1.56462408 -2.36447327

H -1.82398347 -3.26261319 -2.90923641

H -0.18826724 -2.63301863 -2.48941789

H -4.71469722 2.03583685 0.92942367

H -4.71739201 -1.31297683 -1.72946606

H -7.96008131 0.67250804 -2.05514281

H -7.57835254 0.09557225 -0.41170891

H -6.94474151 -0.76919709 -1.82783101

H -6.14733096 1.76461590 -3.48015388

H -5.10401642 0.33893624 -3.27788272

H -4.49420031 1.94635198 -2.83610281

H -7.10674613 2.94082162 -1.53315657

H -5.48846318 3.20595509 -0.85102923

H -6.76402379 2.44997285 0.13838928

H -0.95555741 1.95412259 3.55285840

H -1.07100020 2.10406031 1.77841034

H -0.78463171 0.50603465 2.51015804

H -2.74404914 0.70893414 4.84801186

H -2.61966568 -0.70093574 3.75370906

H -4.19604239 0.09301136 4.01697824

H -3.14507886 2.96762024 3.90915993

H -4.61427081 2.41468524 3.08119678

H -3.33552236 3.25877408 2.16758900

H 4.92678809 -4.13927883 -2.45014879

H 5.91986970 -2.89291377 -3.28640416

H 4.19966817 -3.15020480 -3.74440206

Zn -0.24866220 -1.75325906 0.44024476

C 1.21717352 2.26231411 0.09679347

C 0.86985829 0.83756236 0.36172473

O -0.20369519 0.17666904 -0.33419406

O 1.44115876 0.43207534 1.57481739

C 2.11845759 1.54908408 2.07836262

O 2.82568016 1.46496986 3.16746442

O 1.87847464 2.60545674 1.25586247

H 1.47223376 2.33713487 -0.93966499

C 0.03778276 3.17804971 0.47370955

H -0.69114011 3.16140297 -0.30942156

H -0.40723810 2.83059912 1.38262898

C 0.54799371 4.61781282 0.66958441

C 0.40618216 5.24575710 1.90734323

C 1.15196988 5.29404797 -0.39035282

C 0.86766375 6.54988542 2.08484197

H -0.07071837 4.71263115 2.74255842

C 1.61453390 6.59822518 -0.21268385

H 1.26398074 4.79921613 -1.36591628

C 1.47236359 7.22625215 1.02461431

H 0.75535621 7.04512778 3.06024882

H 2.09107172 7.13101268 -1.04846097

H 1.83620065 8.25448426 1.16482002

TS_SR2_

-2320.637101

C -0.65872100 0.59425100 -1.43440000

C -1.15638300 0.94372600 -0.04588400

O -1.30130100 -0.33333100 0.68554000

O -0.96046700 1.50746900 -2.34866200

C -0.11051700 1.76116000 0.74986500

C 0.28712600 3.04774400 0.05003900

C -0.59107600 4.13874800 0.00607000

C -0.22431000 5.31884300 -0.64110700

C 1.03001900 5.42310000 -1.24881300

C 1.91335400 4.34307300 -1.20262900

C 1.54586500 3.15967800 -0.55602000

C 0.59958600 -4.50384600 0.89607100

C 0.30853600 -3.69843800 2.17380100

N 0.75436400 -2.26355300 2.00479700

N -0.22028600 -3.98032600 -0.26102900

C -1.63015700 -4.50542200 -0.18642400

C 0.40761100 -4.40298500 -1.56890900

C 2.27338900 -2.16592300 2.12221400

C 0.14283700 -1.40870300 3.08122300

C 2.88615900 -0.99926700 1.39460700

C 2.89982700 -1.09643700 -0.02596200

C 3.78607800 -0.23365000 -0.73411000

C 4.44149700 0.78076900 -0.01967300

C 4.31890400 0.96468200 1.36720000

C 3.56299900 0.01541600 2.06691200

C 5.00428700 2.11894500 2.12516600

C 6.01144100 1.54454900 3.15747100

C 3.92522000 2.95574500 2.86527200

C 5.77448300 3.06829800 1.17670900

C 4.08966800 -0.48239900 -2.22619900

C 2.80955900 -0.31591400 -3.08116900

C 4.61648900 -1.93751500 -2.37563400

C 5.16882800 0.47513500 -2.78275100

O 2.10852400 -2.02835000 -0.61096300

O -0.03665100 -0.46475000 -1.66006200

C -0.72153800 1.15096100 -3.78779700

H -2.10181400 1.46331800 -0.14746600

H 0.77025600 1.12927600 0.90394400

H -0.54967200 1.97168600 1.73167400

H -1.56413700 4.06644600 0.48236700

H -0.91231600 6.15601500 -0.66796500

H 1.31670900 6.34100200 -1.74882900

H 2.89095300 4.41947100 -1.66512500

H 2.24129200 2.32721000 -0.51113300

H 0.38046800 -5.56828900 1.05689500

H 1.64644200 -4.39123800 0.60913700

H 0.81635400 -4.15969500 3.03325100

H -0.76525500 -3.66821500 2.36638400

H -2.18532700 -4.10199800 -1.02921600

H -2.10638600 -4.13652300 0.72089900

H -1.61666100 -5.60383700 -0.20650200

H -0.22486600 -4.03575600 -2.38078700

H 0.48249400 -5.49723000 -1.62847800

H 1.39161600 -3.93622700 -1.63365600

H 2.68678600 -3.08026200 1.69009500

H 2.52917500 -2.14177800 3.19048900

H 0.47512800 -0.38093200 2.93040800

H 0.47302900 -1.75547500 4.07122600

H -0.93838300 -1.46851300 2.99174800

H 5.08691600 1.45066200 -0.56831800

H 3.51687700 0.04661700 3.15188900

H 6.49414100 2.36140300 3.70821200

H 6.78308400 0.95862300 2.64641100

H 5.50600900 0.89398200 3.87897300

H 4.39670300 3.78278800 3.41071300

H 3.37681600 2.33696200 3.58347200

H 3.20975000 3.36968900 2.14640100

H 6.22294100 3.87804700 1.76374000

H 5.10095000 3.51491600 0.43662600

H 6.57935200 2.54099400 0.65251800

H 3.03033900 -0.54872300 -4.13118100

H 2.45167300 0.71966400 -3.01881900

H 2.03627000 -0.98208300 -2.70520600

H 4.80777000 -2.15987900 -3.43290400

H 3.86790100 -2.62926000 -1.98540400

H 5.55051400 -2.05872200 -1.81513500

H 5.35199700 0.22442600 -3.83404200

H 6.11397300 0.37469000 -2.23714600

H 4.84004900 1.52043200 -2.73654200

H -0.93971700 2.07216800 -4.32229500

H -1.42812200 0.35373000 -4.02233600

H 0.31109900 0.83328000 -3.92459500

Zn 0.14510000 -1.88535300 -0.08270600

C -2.58125300 -1.11042800 0.78533000

C -3.86661800 -0.42045900 0.33786000

O -3.69629300 0.03519700 -1.03968300

O -2.53573700 -2.00829000 1.62935600

O -2.40029300 -1.84995100 -0.99619200

C -2.96821800 -0.97375800 -1.79931800

O -2.93369500 -0.87513300 -3.01923100

C -4.30753600 0.74286500 1.24175300

H -4.18369300 0.41383600 2.28014800

H -3.64543000 1.60001100 1.08032100

C -5.75449300 1.16237300 1.01673200

C -6.23265500 1.42966500 -0.27541300

C -6.62193100 1.30469600 2.10692300

C -7.55474500 1.83299000 -0.46520500

H -5.56522600 1.29261300 -1.11622000

C -7.94435300 1.71159800 1.91515600

H -6.26200600 1.09501600 3.10912100

C -8.41365900 1.97786600 0.62770100

H -7.91514400 2.03212800 -1.46807700

H -8.60546100 1.81600000 2.76796600

H -9.44011400 2.29169800 0.47668800

H -4.62394000 -1.20970500 0.37970800

TS_SR3_

-2320.642813

C -0.32447200 -0.58153500 -2.41875900

C -1.48381100 0.30154100 -1.98713800

O -1.30332700 0.86455000 -0.64916700

O -0.75411000 -1.37495100 -3.42850000

C -1.62172000 1.50833200 -2.94673300

C -2.93078000 2.25010000 -2.74229000

C -3.16524800 2.98445300 -1.56885100

C -4.37761500 3.65193000 -1.38931300

C -5.36840100 3.59642700 -2.37342200

C -5.14277700 2.86566600 -3.54134800

C -3.93075300 2.19547300 -3.72227500

C 4.13769200 -3.12818900 0.56152600

C 2.94454200 -3.14838600 1.53584800

N 2.21664300 -1.82464300 1.56141900

N 3.72852400 -2.80286700 -0.86228600

C 3.38069600 -4.06171400 -1.60611000

C 4.84510300 -2.06162300 -1.55446200

C 3.07417700 -0.70418400 2.14787800

C 0.97469600 -1.93781300 2.40346000

C 2.54471300 0.66028600 1.80251500

C 2.76289400 1.09513200 0.46782800

C 2.52845800 2.46603100 0.17294900

C 1.90117700 3.25510100 1.14600500

C 1.54753100 2.78564000 2.42025300

C 1.94256200 1.48419800 2.74959700

C 0.75405300 3.62922700 3.43648200

C 1.64057700 3.93829100 4.67213700

C -0.50147300 2.82749600 3.88236100

C 0.27584800 4.97030000 2.83193400

C 3.00786200 3.06976700 -1.16256000

C 2.28038900 2.40282500 -2.35590500

C 4.53513700 2.80946100 -1.29389800

C 2.77449900 4.59640600 -1.24361200

O 3.20696500 0.18805000 -0.44601500

O 0.85914500 -0.55883300 -2.04666200

C 0.21699300 -2.38665000 -3.93389900

H -2.38144400 -0.31059500 -2.04952200

H -1.56422600 1.12440100 -3.97070200

H -0.76334500 2.16190800 -2.76697700

H -2.40697800 3.01015900 -0.79772500

H -4.54816300 4.21582600 -0.47939700

H -6.30717600 4.11935000 -2.23138000

H -5.90562100 2.81843200 -4.31014300

H -3.75616400 1.63199900 -4.63333300

H 4.64137600 -4.10450800 0.58636100

H 4.86053700 -2.37168000 0.87069400

H 3.30706800 -3.40825000 2.54170600

H 2.21335600 -3.89254700 1.21243800

H 3.14511800 -3.79543900 -2.63995400

H 2.49088300 -4.49772600 -1.15659400

H 4.22560300 -4.76422900 -1.59632200

H 4.56357300 -1.91445300 -2.60068000

H 5.78723100 -2.62452700 -1.50839600

H 4.92541700 -1.08408000 -1.07721000

H 4.07062700 -0.79899600 1.71303200

H 3.13406900 -0.85031800 3.23531200

H 0.44160800 -0.98644300 2.34561400

H 1.24665300 -2.12833300 3.45209400

H 0.34385200 -2.75234300 2.04784900

H 1.66815600 4.27914900 0.89564500

H 1.77747100 1.10049600 3.75212200

H 1.06914800 4.50797000 5.41594900

H 2.51618400 4.52563800 4.37382800

H 1.98993200 3.01181100 5.14041600

H -1.13180300 3.44809600 4.53183500

H -0.20932000 1.93536800 4.44775300

H -1.08241600 2.50046600 3.01420200

H -0.30852300 5.51511600 3.58243400

H -0.36027300 4.79746100 1.95681200

H 1.12248100 5.60056500 2.53692100

H 2.66957000 2.80094400 -3.30225200

H 1.20635700 2.61275300 -2.30277500

H 2.43381400 1.32620800 -2.30880300

H 4.90229100 3.20350600 -2.25015700

H 4.71545800 1.73398300 -1.24707300

H 5.07355500 3.30541000 -0.47820700

H 3.17224300 4.96374700 -2.19705100

H 3.28979200 5.12226900 -0.43171500

H 1.70673000 4.84252000 -1.20433700

H 1.07336800 -1.88573000 -4.39036500

H -0.34726000 -2.95406600 -4.67055800

H 0.53241100 -3.00498200 -3.09194500

Zn 2.04943100 -1.45097000 -0.56970800

C -1.83648500 0.25196900 0.49655700

C -2.02144400 -1.26381500 0.45230000

O -0.93056500 -1.72874000 -0.39446800

O -2.08756900 0.94207800 1.46762400

O 0.76606400 -3.13271500 -0.75833700

C -0.39213000 -3.05639500 -0.18187000

O -1.02087100 -3.86353200 0.51191800

C -3.42462200 -1.71301100 -0.06782300

H -3.89826100 -0.91975800 -0.65533100

H -3.24211900 -2.56950600 -0.72492100

C -4.33201500 -2.13163300 1.07443400

C -4.00865100 -3.28420400 1.80878900

C -5.47187300 -1.39745400 1.41330700

C -4.82256400 -3.69161200 2.86459800

H -3.11049200 -3.83271200 1.54203300

C -6.28654300 -1.80812100 2.47262800

H -5.72218800 -0.50314000 0.85221300

C -5.96444700 -2.95553000 3.19859600

H -4.56926300 -4.58368100 3.42637600

H -7.16756600 -1.23126400 2.72981000

H -6.59631000 -3.27429200 4.01969800

H -1.89263000 -1.64265000 1.46656100
